# Proteins from shrews’ venom glands play a role in gland functioning and venom production

**DOI:** 10.1186/s40851-024-00236-x

**Published:** 2024-07-15

**Authors:** Krzysztof Kowalski, Paweł Marciniak, K. Anne-Isola Nekaris, Leszek Rychlik

**Affiliations:** 1 Department of Vertebrate Zoology and Ecology, Institute of Biology, Faculty of Biological and Veterinary Sciences, Nicolaus Copernicus University in Toruń, Lwowska 1, Toruń, 87-100 Poland; 2 Department of Animal Physiology and Developmental Biology, Institute of Experimental Biology, Faculty of Biology, Adam Mickiewicz University in Poznań, Uniwersytetu Poznańskiego 6, Poznań, 61-614 Poland; 3 Centre for Functional Genomics, Department of Health and Life Sciences, Oxford Brookes University, Oxford, OX3 0BP UK; 4 Department of Systematic Zoology, Institute of Environmental Biology, Faculty of Biology, Adam Mickiewicz University in Poznań, Uniwersytetu Poznańskiego 6, Poznań, 61-614 Poland

**Keywords:** Eulipotyphlans, Immune response, Metabolism, *Neomys fodiens*, Oral venom system, *Sorex araneus*, Stress response, Venom evolution

## Abstract

Venom production has evolved independently many times in the animal kingdom, although it is rare among mammals. Venomous shrews produce venom in their submandibular salivary glands and use it for food acquisition. Only a few toxins have been identified in shrew venoms thus far, and their modes of action require investigation. The biological and molecular processes relating to venom production and gland functioning also remain unknown. To address this gap, we investigated protein content in extracts from venom glands of two shrew species, *Neomys fodiens* and *Sorex araneus*, and interpreted their biological functions. Applying a proteomic approach coupled with Gene Ontology enrichment analysis, we identified 313 and 187 putative proteins in venom glands of *N. fodiens* and *S. araneus*, respectively. A search of the UniProt database revealed that most of the proteins found in both shrew species were involved in metabolic processes and stress response, while GO enrichment analysis revealed more stress-related proteins in the glands of *S. araneus*. Molecules that regulate molecule synthesis, cell cycles, and cell divisions are necessary to enable venom regeneration and ensure its effectiveness in predation and food hoarding. The presence of proteins involved in stress response may be the result of shrews’ high metabolic rate and the costs of venom replenishment. Some proteins are likely to promote toxin spreading during envenomation and, due to their proteolytic action, reinforce venom toxicity. Finally, finding numerous proteins involved in immune response suggests a potential role of shrew venom gland secretions in protection against pathogens. These findings open up new perspectives for studying biological functions of molecules from shrew venom glands and extend our knowledge on the functioning of eulipotyphlan venom systems. Because the majority of existing and putative venomous mammals use oral venom systems to inject venom into target species, the methods presented here provide a promising avenue for confirming or discovering new taxa of venomous mammals.

**Supplementary Information:**

The online version contains supplementary material available at 10.1186/s40851-024-00236-x.

## Background

Venom production is a complex functional trait that has evolved independently on many occasions in the animal kingdom, although it is rare among mammals [1–3]. Venomous mammals have been found in only four orders to date : monotremes (platypus), eulipotyphlans (solenodons and shrews), chiropterans (vampire bats), and primates (lorises) [3–6]. Most confirmed and putatively venomous mammal species are eulipotyphlans [3, 7].

Intriguingly, despite eulipotyphlans occupying broad trophic niches, their venoms seem to have relatively simple composition, with only a few toxins participating in food acquisition and storage [3, 7–9]. *Blarina* toxin (BLTX), *Blarina* paralytic peptides (BPPs) 1 and 2, soricidin (SOR), kallikrein 1 serine proteases (KLK1s), phospholipase A_2_ (PLA_2_), antileukoproteinase (SLPI), tissue factor pathway inhibitor 2 protein, proenkephalin A, lysozyme C, disintegrin and metalloproteinase domain-containing proteins (ADAMs), β-defensins, BQTX and non-toxic blarinasin and hyaluronidase, have been recently found in eulipotyphlan venoms [7–15]. These components are linked to the toxic activities of these venoms, such as paralysis and convulsions, irregular respiration, inhibition of heart rate, increase in vascular permeability, decrease in blood pressure, haemolysis, and death [7, 9, 10, 14]. Hyaluronidase commonly occurs in venoms of various animal taxa, but is devoid of toxic activity [16, 17]. Instead, due to its ability to hydrolyse connective tissue, it promotes the spreading of toxins after envenomation [8, 18]. It is noteworthy that SOR and BPP2 from the venom of the short-tailed shrew, *Blarina brevicauda*, are the only eulipotyphlan toxins for which the molecular mechanisms of action have been established. SOR inhibits the transient receptor potential of vanilloid type 6 (TRPV6) calcium channels [12], while BPP2 causes a hyperpolarisation of human T-type Ca channel hCa_v_3.2 activation [15]. Modes of action of other eulipotyphlan toxins still wait to be revealed.

**Fig. 1:**
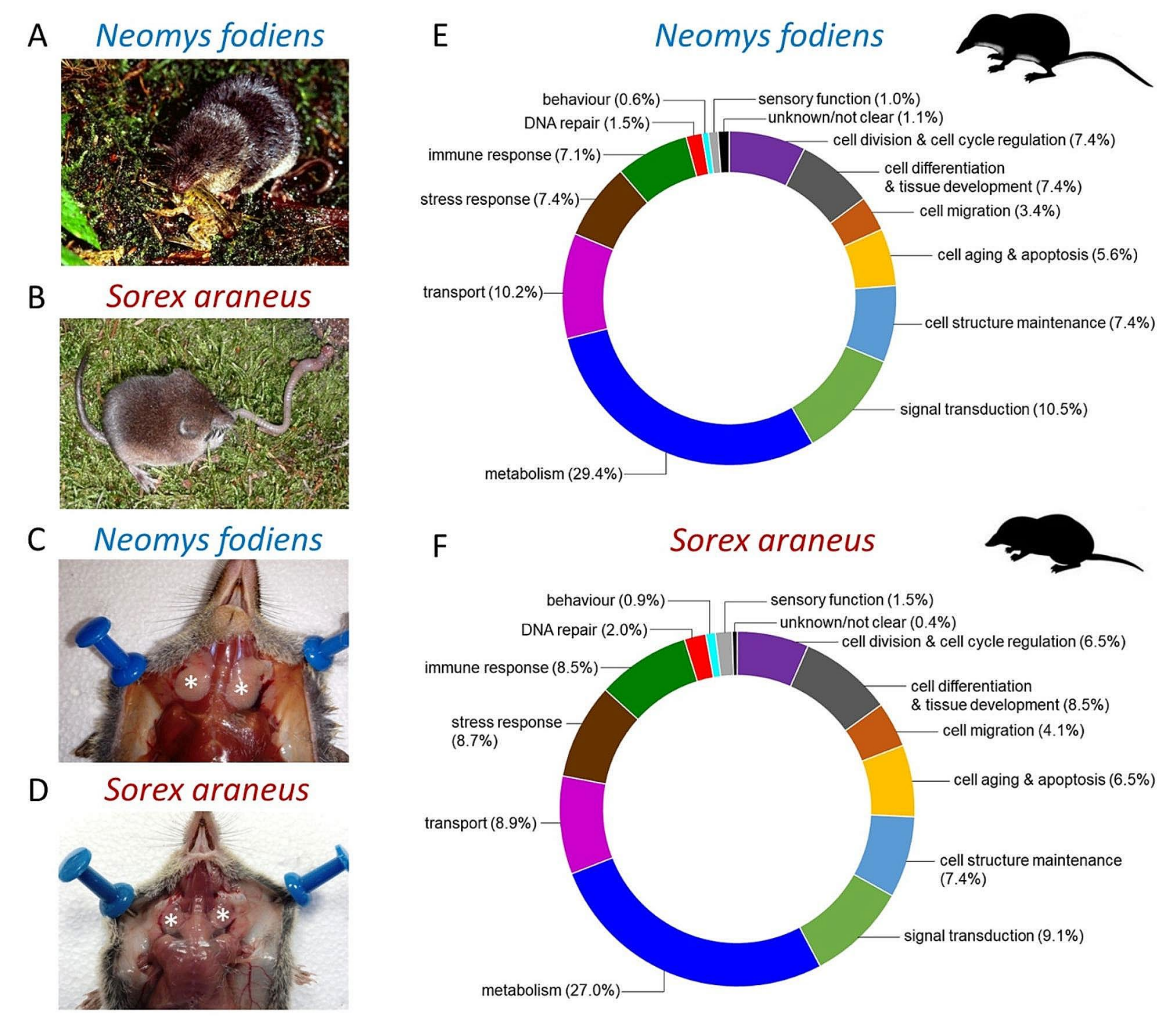
Functional assessments of extracts from shrews’ venom glands reveal putative proteins involved in cell metabolism, epithelial cell turnover, stress response, immune response and venom gland functioning. (**A**) The Eurasian water shrew (*Neomys fodiens*) hunting a frog, and (**B**) the common shrew (*Sorex araneus*) devouring an earthworm. Photos by Marc Mounier (**A**) and Sophie von Merten (**B**). Photos **A** and **B** were first published in Kowalski et al. [7]. (**C** and **D**) The submandibular (venom) glands (marked with asterisks). Photos by Krzysztof Kowalski. (**E** and **F**) Biological functions of proteins identified in the extracts from venom glands. Functions were determined through homology to other venomous animals (including eulipotyphlans) and nonvenomous mammals by searching the UniProt database. Values in parentheses show percentage of proteins classified into each function category. Detailed functions of particular proteins are provided in Additional file 3: Table A3 and Additional file 5: Table A5

Most studies dealing with eulipotyphlan venoms have focused on biochemical and pathophysiological aspects of venoms [3, 14, 19, 20]. Little is known about the functioning of the submandibular salivary glands in which toxic saliva is produced. The processes involved in venom production and excretion also remain unknown. Particularly, molecular mechanisms that regulate the function of venom glands and toxin synthesis remain to be ascertained. The first attempts to characterise components of mammalian saliva were made by Dufton [4]. Amylase, DNAase, peptidase, lysozyme and epidermal growth factor were found in the extracts from parotid and submaxillary glands of eulipotyphlans, mainly shrews. Recently, Kowalski et al. [7] identified 194 proteins in the extract from venom glands of the Eurasian water shrew (*Neomys fodiens*) (Fig. 1A) and 112 proteins in the common shrew (*Sorex araneus*) (Fig. 1B). However, no in-depth functional or molecular analyses have been performed to ascertain the biological or molecular functions of these proteins. To address this gap and better understand the role of molecules in gland functioning, toxin synthesis, and venom production and secretion, we aimed to investigate protein content in extracts from venom glands of two shrew species, *N. fodiens* and *S. araneus* (Fig. 1C and D), and describe their biological and molecular functions, applying a proteomic approach coupled with Gene Ontology enrichment analysis.

We report here 313 and 187 putative proteins in the venom glands of *N. fodiens* and *S. araneus*, respectively. Most of the proteins identified in *N. fodiens* are linked to metabolic processes and cellular stress response, while in *S. araneus* principally to the latter. Numerous proteins are also involved in immune response indicating a potential role of shrew venom gland secretions in protection against pathogens.

## Results

### Proteins from the venom glands of *N. fodiens* link to cell metabolism, stress, immune response and oxidation-reduction processes

We identified 313 proteins in the extract from venom glands of the water shrew (Additional file 1: Table A1), including four putative toxins: proenkephalin A, phospholipase A_2_ (PLA_2_), lysozyme C and disintegrin and metalloproteinase domain-containing protein (ADAM) (Table 1), and hyaluronidase, a toxin spreading factor that commonly occurs in the venoms of various animal taxa. For each identified protein, at least one biological function was assigned by searching UniProt database. All functions identified were assigned to one of 14 categories (Additional file 2: Table A2 and Additional file 3: Table A3). Searching UniProt database revealed that most proteins were involved in cell metabolism (29.4%), signal transduction (10.5%), transport (10.2%), stress response (7.4%), cell structure maintenance (7.4%), cell division and cell cycle regulation (7.4%), cell differentiation and tissue development (7.4%), and immune response (7.1%) (Fig. 1E). The most abundant (i.e., those with the highest emPAI) were proteins related to cell metabolism, stress response, immune response, signal transduction and transport (Additional file 1: Table A1).

**Table 1:** Toxins identified in extracts from venom glands of the Eurasian water shrew (*Neomys fodiens*) and the common shrew (*Sorex araneus*)

Shrew species	Accession code	Matched peptides	Protein sequence coverage [%]	Identified peptides	Protein name
***Neomys fodiens***	P01211	6	9	K.LPSLKTWETCK.E K.KYGGFMK.R K.YGGFMK.R K.RYGGFLK.R	Proenkephalin-A
	P14422	6	6	FAKFLSYK	Phospholipase A_2_
	Q9Z0F8	12	9	SEDIKDFSR	Disintegrin and metalloproteinase domain-containing protein 17
	P12067	6	8	YWCNDGK	Lysozyme C
	gi|521,028,001	14	11	KDIEFYIPK	Hyaluronidase PH-20^a^
***Sorex araneus***	P01211	6	8	YGGFMK + Oxidation (M)	Proenkephalin-A
	Q61754	11	7	DKSNDLMLLR	Kallikrein 1-related peptidase b24
	Q91V70	9	13	FQIPEK	Beta-defensin 7
	Q10741	9	11	LYSDGKK	Disintegrin and metalloproteinase domain-containing protein 10
	P12067	10	19	AWVAWR	Lysozyme C

^a^Hyaluronidase is devoid of toxic activity and acts as a toxin spreading factor commonly found in animal venoms

**Fig. 2:**
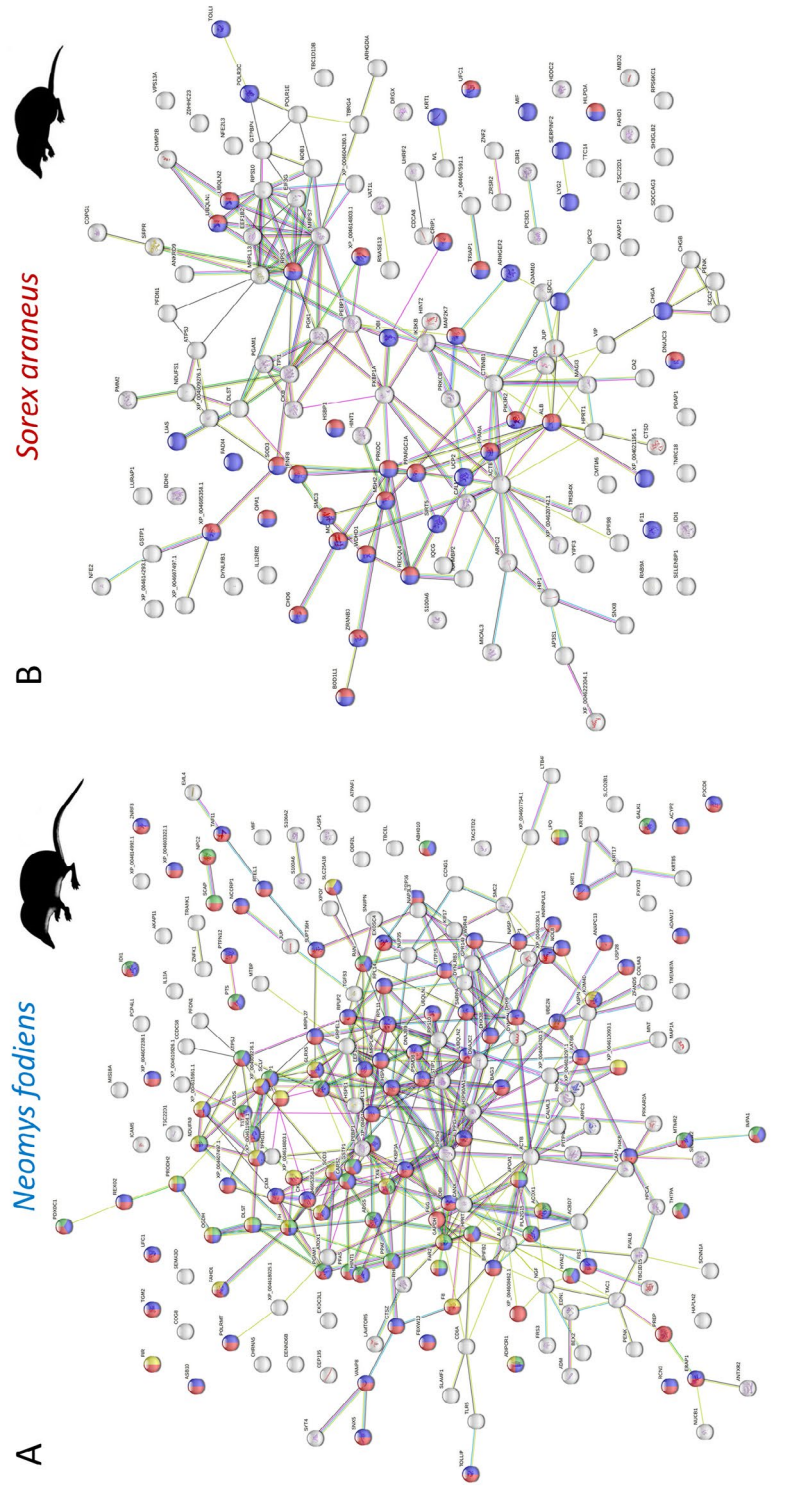
Assessment of protein association in shrews’ venom glands reveals proteins involved in metabolism processes and stress response. (**A**) STRING protein-protein interaction network predicted for proteins identified in the extract from venom glands of the water shrew (*Neomys fodiens*) based on homology to the common shrew (*Sorex araneus*). Red balls represent proteins involved in metabolic processes, blue in cellular metabolic processes, green in small molecule metabolic processes and yellow in oxidation-reduction processes. Lines show interactions between proteins. Abbreviations next to the balls represent protein labels. Full protein names and their labels are provided in Additional file 1: Table A1. (**B**) STRING protein-protein interaction network predicted for proteins identified in the extract from venom glands of the common shrew (*Sorex araneus*). Red and blue balls represent proteins involved in stress response. Lines show interactions between proteins. Abbreviations next to the balls represent protein labels. Full protein names and their labels are provided in Additional file 4: Table A4

**Fig. 3:**
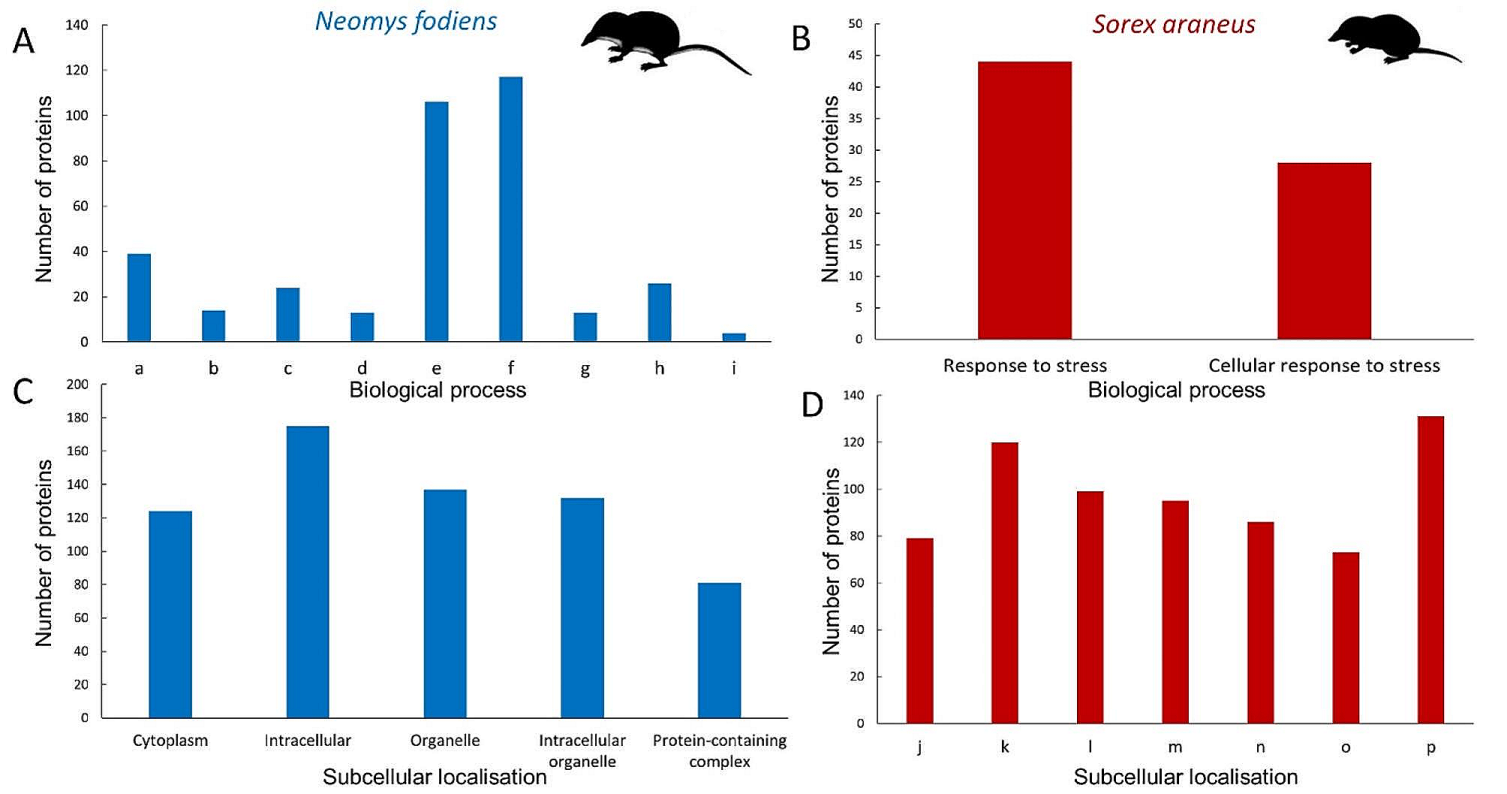
Gene Ontology (GO) enrichment analysis of proteins from shrews’ venom glands reveals proteins involved in metabolism processes and stress response. (**A** and **B**) Number of proteins involved in biological processes predicted based on the GO enrichment analysis of proteins identified in the extracts from venom glands of the Eurasian water shrew (*Neomys fodiens*) (**A**) and the common shrew (*Sorex araneus*) (**B**). Abbreviations on the panel **A**: a – Small molecule metabolic process; b – Purine nucleotide metabolic process; c – Organophosphate metabolic process; d – Purine ribonucleotide metabolic process; e – Cellular metabolic process; f – Metabolic process; g – Generation of precursor metabolites and energy; h – Oxidation-reduction process; i – IMP metabolic process. (**C** and **D**) Localisation of proteins identified in the extracts from venom glands of the water shrew (**C**) and the common shrew (**D**) within the cell based on the Gene Ontology component analysis. Abbreviations on the panel **D**: j – Cytoplasm; k – Intracellular; l – Organelle; m – Intracellular organelle; n – Membrane-bounded organelle; o – Intracellular membrane-bounded organelle; p – Cellular anatomical entity

STRING protein-protein interaction network predicted for proteins identified in the extract from venom glands of *N. fodiens* confirmed the results obtained by searching the UniProt database. Most proteins were related to metabolic and oxidation-reduction processes (Fig. 2A). Similarly, the results of GO enrichment and KEGG analyses are consistent with previous findings showing that identified proteins were involved in metabolic pathways and cell stress response (PPI enrichment *p*-value: 0.0007; Fig. 3A). All identified proteins were localised within the cell (Fig. 3C).

### Proteins from the venom glands of *S. araneus* are involved in stress response

We identified 187 proteins in the extract from venom glands of the common shrew (Additional file 4: Table A4), including five toxins: proenkephalin A, disintegrin and metalloproteinase domain-containing protein (ADAM), kallikrein 1-related peptidase (KLK1), lysozyme C and beta-defensin-7 (Table 1). As in the case of *N. fodiens*, for each identified protein at least one biological function was assigned by searching UniProt database. Next, all functions were assigned to one of 14 categories (Additional file 2: Table A2 and Additional file 5: Table A5). Searching UniProt database revealed that most proteins were involved in cell metabolism (27.0%), signal transduction (9.1%), transport (8.9%), immune response (8.5%) and cell differentiation and tissue development (8.5%) (Fig. 1F). The most abundant (with the highest emPAI) were proteins related to cell metabolism, signal transduction, transport, stress response and immune response (Additional file 4: Table A4).

The STRING protein-protein interaction network predicted for proteins identified in the extracts from venom glands of *S. araneus* revealed that most proteins were related to stress response (Fig. 2B). Moreover, the results of GO enrichment and KEGG analyses are consistent with this finding (PPI enrichment *p*-value: 0.04; Fig. 3B). Similar to the case in the venom glands of *N. fodiens*, all identified proteins were localised within the cell (Fig. 3D).

## Discussion

Eulipotyphlan venoms have largely gone unstudied, with only a few toxins being characterised thus far [7–10, 12, 14, 15]; their modes of action also remain unexplored. Similarly, the mechanisms that drive processes related to venom production and secretion still require investigation. Our research is the first comprehensive work providing a list of putative molecules that control functioning of shrew venom glands. Applying a proteomic approach coupled with GO enrichment analysis, we found 313 and 187 proteins in venom glands of *N. fodiens* and *S. araneus*, respectively. Some of these compounds have been previously reported in extracts from venom glands of these two shrew species [7].

### Proteins from the venom glands of *N. fodiens* are involved in venom gland functioning and epithelial cell turnover

Searching UniProt database alongside GO enrichment analysis showed that most proteins in the venom glands of *N. fodiens* are related to metabolism, especially molecules synthesis and glycolysis. It must be emphasized that proteins identified by us have more interactions among themselves than would be expected for a random set of proteins of the same size and degree of distribution drawn from the genome (observed number of edges: 364 vs. expected number of edges: 296). Such an enrichment indicates that our proteins are at least partially biologically connected, as a group [21]. We found the same for proteins identified in the venom glands of *S. araneus* (observed number of edges: 187 vs. expected number of edges: 164).

Proteins extracted from submandibular glands of *N. fodiens* being involved in molecule synthesis, cell division and differentiation may be indicative of a high epithelial cell turnover. Such an intense cell turnover in venom glands should require well-developed protein machinery that regulates cell cycles and cell divisions to enable quick replenishment of toxins and thus increase the effectiveness of venom in prey hunting and food storing [22–26]. Additionally, two proteins, transforming growth factor beta-3 and protein-glutamine gamma-glutamyltransferase 2, are noteworthy, as they are involved in salivary gland morphogenesis (Additional file 3: Table A3).

### Proteins from the venom glands of *S. araneus* participate in stress response

In *S. araneus*, according to searches in UniProt database, most proteins also participate in metabolic processes. Contrary, GO enrichment and STRING protein-protein interaction network analyses revealed that the most frequent are proteins involved in stress response. Shrews are known to have high metabolic rates due to their very high energy demands [27, 28], which creates the need for constant feeding and hoarding prey items for later consumption [3, 23, 26]. Venom that paralyses prey and enables gathering it in a comatose state is a very important evolutionary trait that helps shrews to maximise foraging gain, meet their high energy requirements and thus maintain body mass and eventually survive [3, 9, 23]. However, according to the venom optimisation hypothesis [29, 30], venom production is metabolically costly [31, 32]. Therefore, the need to regenerate venom rapidly, together with the fast pace of life [33], may generate chronic stress in shrews [34–37]. Molecular mechanisms that regulate stress responses are thus expected to be well-developed in such tiny and fast-lived mammal species as shrews.

Interestingly, we confirmed the presence of more proteins related to stress response in the glands of *S. araneus* than in the water shrew. Smaller shrews have higher basic metabolic rates than larger ones [28], and therefore must consume and store larger amounts of food [26, 27]. Indeed, common shrews are known for gathering many, usually smaller, prey items [23]. Frequent hunting and gathering prey requires more energy for food foraging and multiple transport of prey items to the shelter. Chewing and food digestion also affect the energy budgets of shrews, and chewing may induce stress response in salivary duct cells [38]. Frequent hunting creates a need to leave the shelter more often. Outside the shelter, shrews are at higher risk of predation, and competition for food resources between shrews increases [3, 23]. Common shrews usually occur in higher population densities than do water shrews [27, 39], and in near-water areas they often coexist with larger *N. fodiens* (this was the case with the animals used in this study), which dominate common shrews behaviourally [40]. Therefore, social and interspecies interactions may be more frequent and intense in populations of *S. araneus* than in *N. fodiens*, which in turn may provoke more frequent conflicts and thus may induce higher stress levels.

Water shrews are semiaquatic and well-adapted for swimming and diving under water [27]. They hunt both terrestrial and aquatic prey, which they eat in approximately equal proportions, making their diet more diverse than that of *S. araneus*, which feeds almost exclusively on terrestrial prey [41–43]. Thus, eating aquatic prey may require an extra set of proteins and enzymes in their saliva to enable them to effectively digest aquatic prey items. It has been also shown that semiaquatic shrews have larger brains than terrestrial ones, because during diving and hunting under water they must coordinate their movements in three dimensions, while terrestrial shrews move only in two dimensions [44–46]. Therefore, the larger number of proteins related to metabolism in *N. fodiens* may result from the greater energy expenditure during swimming and diving. Moreover, frequent hunting and exposure to (usually cold) water expose water shrews to greater heat loss [27, 47, 48]. Therefore, many metabolism-related proteins may help to increase the metabolic rate quickly and thus replenish heat loss.

Finally, costs of venom production, especially those related to toxin synthesis, epithelial cell turnover in the salivary glands, and toxins release, may be higher in smaller (*S. araneus*) than larger shrews (*N. fodiens*). Such a considerable impact on the energy budget may induce acute and/or chronic stress in shrews, and smaller shrews may experience higher stress levels than larger ones. All the factors mentioned above may explain why smaller shrews, such as *S. araneus*, have well-developed protein machinery that enables them to effectively cope with different stressors and helps them to maintain homeostasis, also in the salivary glands.

Here, we identified many molecules (e.g., superoxide dismutase, thioredoxin, peroxiredoxin-1, glutathione S-transferase P, glutathione peroxidase 1, carboxylesterase 3A, and heat shock proteins (HSPs)) that are involved in cellular stress response in shrew venom glands. Peroxidases are widely distributed within different families of mammals. For instance, they were confirmed in the submandibular glands of rats, mice, guinea pigs, goats, cows, pigs, cats and humans [49, 50]. Glutathione peroxidase, superoxide dismutase and glutathione S-transferase have been reported for rats and vampire bats [51]. Peroxidases catalyse many important reactions for the metabolic regulation of the cell as well as for the defence mechanism that they may be regarded as one of the most important members of the oxidoreductase system of the cell [49]. Salivary peroxidases protect the cells of the salivary glands and the mucosal cells of the oral epithelium from hydrogen peroxide toxicity. The most important part of the intracellular defence against oxygen toxicity is the action of the following enzymes: superoxide dismutase, catalase and glutathione peroxidase [50]. If these enzymes work efficiently, hydroxyl radicals will not be formed, which in turn prevents the cell from damage [50]. Finally, because salivary peroxidases are able to kill bacteria that might grow in mouth, their primary function in salivary glands is to maintain the oral hygiene [49].

Intriguingly, glutathione S-transferase P (here found in venom glands of *S. araenus*) that participates in cellular response to amino acids, reactive oxygen species and toxic substances (Additional file 4: Table A4), as well as phospatidylethanolamine-binding protein 1 identified in *N. fodiens*, may be likely to protect the cell against self-toxification. Further research is required to confirm if these molecules are involved in this process, what their mechanisms of action are and how they could prevent self-toxification in shrews.

Heat shock proteins (HSPs), for instance found in rodent saliva [52, 53], can modify functions and density of other proteins, and thus play an important role in numerous physiological processes. In general, they serve as house-keeping proteins in the cell, but are also involved in immunity. It has been shown that the immune system adopted certain HSPs for its own particular processes [54], and that HSPs may exhibit both pro- and anti-inflammatory properties [54, 55]. Finally, HSPs serve as important antigens of infectious agents, and perhaps, of transformed cells [54]. Indeed, mammalian cells express increased HSP levels after infection with a variety of viral pathogens, and it has been suggested that it adds to resistance at an early stage of infection. For instance, HSP70 and HSP60 represent major targets for antibodies in many infections with helminths, protozoa and bacteria, including *Plasmodium falciparum*, *Trypanosoma cruzi*, *Leishmania donovanii* or *Borrelia burgdorferi* [54]. Because shrews are known to be reservoir hosts for different parasites (e.g., *Trypanosoma* sp., *B. burgdorferi*, cestodes, nematodes, arthropods) [56–62], HSPs present in their salivary glands’ cells may serve as an important mechanism that helps to cope with pathogen infections, and consequently to reduce stress. Similar mechanisms have been found in the submandibular glands of rodents [52–54].

Nerve growth factors (NGFs), here identified in venom of *N. fodiens*, among others participate in cell growth and apoptosis, signalling pathways and nerve development. These proteins have been found in submandibular glands of other nonvenomous shrews such as *Suncus murinus* and *Crocidura horsfieldi* [63, 64], and in rodents (rats, mice, hamsters, and voles) [64–66]. NGFs are also important components of snake venoms [67]. NGF expression is increased in mice submandibular salivary glands in response to chronic stress. Such an elevated expression of NGF helps to cope with stress and maintain homeostasis in the salivary glands [38].

Our findings show that proteins linked to stress response are major components of shrews’ venom glands, especially in *S. araneus*, which may result from high energy demands of these tiny mammals. Alternatively, venom replenishment, toxin synthesis and secretion are supposed to induce cellular stress response. Thus, well-developed stress response mechanisms in venom glands are expected to prevent glands from cell damage [68]. A range of different stressors, such as heat, cold, viral infections, cytokines, oxidative stress, ionization, UV radiation, exposure to toxins and certain metals, may induce the expression of proteins in the salivary glands and thus affect their functioning [55]. Other factors, such as sex, age or dietary habits of studied animals, should be considered in research aiming to determine protein profiles of venom and salivary glands [69]. In this work, to successfully perform chromatographic separation of shrews’ venoms, we had to combine extracts from submandibular glands of 10 individuals, which made it difficult to determine the effects of shrew’s sex and age on the protein content in their venom glands. Therefore, we suggest using more animals in the future research to investigate the differences in protein profiles between males and females as well as juvenile and adult shrews, keeping in mind that sample sizes of captured and killed mammals (especially those under protection) for this research will be limited. Moreover, shrews do not hibernate and are active during winter. Due to seasonal differences in food availability, their diet composition might differ depending on the season [47, 70, 71]. Therefore, seasonal variation in toxin and protein content in the salivary glands should be taken into consideration as well.

### Proteins from shrews’ venom glands may contribute to protection against bacteria and viruses

Particularly noteworthy is the presence of well-developed protein networks related to immune response in venom glands of both shrew species. Shrew venom glands are modified submandibular salivary glands [4, 72–74], and saliva of many mammalian species is known to have antiseptic properties to prevent the growth of disease-causing microorganisms effectively [75]. In this work, we found many proteins that participate in defence response against bacteria and viruses. For instance, interleukin-12 (IL-12), lipopolysaccharide-binding protein, interferon alpha-B, and MHC class I antigen are known to display antimicrobial effects. Also, lysozyme is known to exert such activity [76, 77]. The latter has been already reported for shrew saliva [4, 7, 9]. Other proteins, such as macrophage migration inhibitory factor, peroxisome proliferator-activated receptor alpha, interferon-activable protein 202 or signalling lymphocytic activation molecule trigger inflammatory responses (Additional file 5: Table A5). Some venom components from saliva of *Blarina* and *Neomys* have been previously suggested to exhibit antimicrobial activity [7, 9], but their role in protection against pathogens and maintaining oral hygiene is unclear and still requires examination. These results indicate that some molecules produced in shrew venom glands are likely to contribute to the protection against bacteria and viruses. Further studies are required to investigate how they, and consequently the venom itself, may help shrews and other eulipotyphlans in maintenance of oral hygiene and development of immune response.

### Proteins from shrews’ venom glands link to toxin secretion and venom toxicity

In both shrew species we found many proteins related to protein secretion and transport. The latter account for 10.2 and 8.9% of the total proteins identified in venom glands of *N. fodiens* and *S. araneus*, respectively. Among molecules involved in transport, we found e.g., haemoglobin subunit alpha and kinesin-like protein KIF17 in venom glands of *N. fodiens*, while secretogranin-1, here reported for *S. araneus*, promotes secretion. Some proteins, like anthrax toxin receptor 2 and 78 kDa glucose-regulated protein, are known to transport toxins (Additional file 3: Table A3). Further research should clarify if these proteins may promote spreading toxins into prey species during envenomation.

Because salivary glands act as venom glands in shrews, their secretions (and toxic saliva itself) are expected to contain proteins with enzymatic activities. Previously, Dufton [4] reported amylase, peptidase and DNAase in eulipotyphlan saliva. Here, we found alpha-amylase 1 in venom glands of *S. araneus* that participates in carbohydrate metabolism and cell defence in response to bacteria. Cathepsin Z, prolyl endopeptidase (both found in venom glands of *N. fodiens*), and cathepsin D and mast cell protease 4 (both found in venom glands of *S. araneus*) cause proteolysis (Additional file 3: Table A3 and Additional file 5: Table A5). Thus, due to their proteolytic actions, these molecules are likely to reinforce the toxicity of venoms of both shrew species.

Apart from structural and functional proteins, we also identified a few putative toxins in the extracts from venom glands of both shrew species. In *N. fodiens* venom we found four toxins, including proenkephalin A, PLA_2_, lysozyme C and ADAM (Table 1), while in *S. araneus* venom we found five toxins: proenkephalin A, KLK1, ADAM, lysozyme C, and beta-defensin (Table 1). Proenkephalin, which contains the known toxin peptide soricidin, and KLK1 have recently been characterized in the venom of *B. brevicauda* [8]. PLA_2_, here found only in *N. fodiens* venom, is widely distributed among elapid and viperid snake venoms [78, 79], and is known for various toxic activities, including neuro- and cytotoxicity [78, 79]. Metalloproteinases (ADAMs) commonly occur in venoms of different animal taxa, including snakes, scorpions and jellyfish [80–83]. They display cytotoxicity [18, 84, 85] and have recently been reported in venoms of *N. fodiens* and *S. araneus* [7]. All these toxins are responsible for shrew venom toxicity, but the mechanisms of their action still require investigation. In the extract from venom glands of *N. fodiens*, we also found a hyaluronidase that is devoid of toxic activity but is able to hydrolyse connective tissue [18]. Therefore, hyaluronidase acts as toxin spreading factor facilitating the action of other venom components during envenomation [18].

### Structure and functions of the mammalian salivary glands

Salivary glands are complex because they are structurally diverse, evolve rapidly, and clearly have several biological roles. Additionally, they undergo phenotypic plasticity [86]. On the basis of size and their distance from the oral mucosa, salivary glands are classified as either major or minor. Nearly all mammals have three paired sets of major salivary glands (the parotid, submandibular, and sublingual glands) as well as hundreds of minor salivary glands that underlie all the oral mucosa with the exception of the gingiva, anterior part of the hard palate and the dorsum of the body of the tongue [86, 87]. Some carnivores (e.g., ferrets, minks, domestic dogs and cats), lagomorphs and some ruminants have two additional major salivary glands: molar and zygomatic [87, 88]. In some mammals (especially in many bat species), the submandibular glands exist in a tandem form comprising principal and accessory glands; in a few species, the parotid gland also may have a binary structure [86].

Histologically, salivary glands are composed of secretory cells (serous or mucous) grouped into larger units, the so-called lobules. The content from individual units moves to the intralobular ducts and flows successively through ducts of increasingly larger diameter, until the main exit duct ending in the oral cavity [87, 89]. Secretory endpieces may assume several different shapes, depending on the gland and the nature of the secretory product. The most common configurations are globular (also called acini) or they may be in the form of tubules. In some glands, the endpieces may be an amalgam of the two shapes. In many animals, the endpieces may be capped by a second type of secretory cell in the form of demilunes [86]. The cells that compose the endpieces are classified according to their secretory products. Serous cells, which produce a protein-rich secretion that may include enzymes, are basophilic and contain many dense secretory granules. Mucous cells produce a secretion rich in glycoconjugates, and their granules tending to fuse into a single mass. The third category of secretory endpiece cells, seromucous cells, share some of the features of each of the other two types [86].

Originally, the structure of the salivary gland complex in vertebrates was thought to be related primarily to the type of food consumed. For example, the proportion of serous secretion is larger where food is bulky, as in the plant-eaters [90]. However, in many mammals the phenotypic variation of salivary gland cannot be explained simply by diet [86]. The gland structure varies also with the way of life. For example, in aquatic mammals, where soaking the food is not needed, gland structures are vestigial. Their structure may also vary considerably with the age of individual [90].

Reptiles do not have the large salivary glands seen in mammals, but they possess glands of the serous and mucous types. Three pairs of salivary glands are present in the monotremes, echidnas, and at least some of the marsupials. The echidna and the platypus have no acid-secreting cells in the stomach, so salivary glands secreting digestive enzymes seem necessary to them [90]. The parotid of the echidna, *Tachyglossus aculeatus*, is a typical serous gland with tubulo-acinar secretory endpieces and a well-developed system of striated ducts. Histologically, the gland is lobulated. The mandibular gland, although resembling a mucous gland, secretes very little glycoprotein. Its cells are packed instead with serous granules, resembling those in the mandibular gland of the European hedgehog (*Erinaceus europaeus*). The sublingual glands secrete an extremely viscous mucous saliva. Expulsion of this saliva through the narrow ducts is probably aided by contraction of the extensive myoepithelial sheaths surrounding the secretory tubules [91].

In the Xenarthra, the submaxillary glands are usually large and the parotid glands are small. In the anteaters, which have very long tongues profusely covered with saliva for taking their prey, the submaxillary gland has an additional bladder-like receptacle functioning as “reservoir” to hold saliva in readiness individual [90].

Among the rodents, those species that are exclusively herbivorous (e.g. the beaver) have large parotid glands, but in rats, parotid and submaxillary glands are of nearly equal size. The ruminants, which require a large amount of watery fluid to mix with their often relatively dry food, have very well-developed glands. The ungulates have parotid glands about four times the size of the submaxillary glands. The secretion mixed with the food to form the “cud” is a highly watery one [90]. Certain breeds of sheep (and possibly cattle) have a unique type of salivary gland, situated at the base of the tongue, which has a very high concentration of lipase [90].

Bats (Chiroptera) have a larger parotid gland in the frugivorous forms and smaller ones in the insectivorous forms. Blood-sucking bats have anticoagulants in their saliva [90]. The luminal surface of endpiece cells usually bears a few stubby microvilli, but these are extremely abundant in submandibular glands of the common vampire bat (*Desmodus rotundus*). The basal surfaces of endpiece cells, especially those that are either serous or seromucous, exhibit considerable interspecific variation. In frugivorous bats, these surfaces are smooth, whereas in insectivorous and carnivorous bats they have an elaborate array of slender basal folds that intermesh with similar folds from adjacent cells [86]. The Neotropical fringe-lipped bat (*Trachops cirrhosus*) feeds on frogs, many of which have toxins in their skin. Probably for this reason, *T. cirrhosus* has a distinctly modified additional submandibular salivary gland, in which the secretory ends have a follicular architecture. The accessory submandibular glands of frog-eating bats from Asia and Africa have a similar structure, although they evolved independently of *Trachops* [86].

The main and most common functions of saliva and salivary glands include: (1) production of digestive enzymes that serve to pre-digest food; (2) saliva moisturizes (lubricates) the food and dissolves it, making it easier to swallow; (3) it moisturizes the inside of the mouth and throat; (4) it maintains appropriate pH in the oral cavity; (5) it has a bactericidal effect (thanks to such antimicrobial agents as immunoglobulin A, lysozyme and lactoperoxidase), which protects the oral mucosa against various potentially harmful factors; (6) protects teeth against bacterial attack which prevents tooth decay; (7) it cleans the oral cavity by flushing away food debris; (8) allows taste perception by solubilizing food chemicals, an essential step for the stimulation of receptor cells of the taste buds [87, 89, 90, 92, 93]. There are also more specific functions that may apply only to some mammal species. Some mammals use saliva as a territorial marker. In others, saliva affects social and sexual behaviour, and thus reproduction. Saliva helps in maintaining the integument of newborn and infant offspring, as well as ensures the therapeutic effects of wound licking [93]. Vampire bats secrete a strong plasminogen activator in their saliva, which breaks down fibrin during its formation, thanks to which blood continues to flow freely from bite wounds. Several species of mammals have toxic saliva, which they use to increase the virulence of the bite. Saliva may neutralize toxins (e.g., from the skin glands of some frogs) [92]. The echidna may use saliva to prevent ants from stinging (lending an additional protective function of saliva) [90].

In addition to these functions based on exocrine phenomena, the salivary glands secrete a number of growth factors and vasoactive substances that are released indirectly into the bloodstream rather than into the oral cavity. In addition, many hormones secreted by other glands (endocrine) may appear in saliva [86, 87, 92].

### Link between protein functions and morphology of shrew submandibular glands

All of the extant venomous eulipotyphlans produce venom in submandibular glands [3, 7, 9, 19, 20, 94]. Histological studies on glands of *B. brevicauda*, *N. fodiens* and *Solenodon paradoxus* revealed unusual and enlarged segments of granule-filled cells in the tubes between the intralobular ducts and the terminal acini [95]. A granular segment in the gland of *N. fodiens* is clearly similar to that described in *Blarina*, but the granule-filled cells are not as enlarged and the secretory granules are smaller. In *Solenodon* the submandibular glands are proportionally larger than in shrews, and their secretory ducts contain large cells with acidophilic granules [94], which are considered the source of venom [96]. Similar but less fully developed segments have been also found in other shrew species in which no venom is produced. For instance, submandibular glands of *Sorex fumeus*, *S. pacificus*, *S. palustris*, *S. sinuosus*, *S. trowbridgii*, *S. vagrans*, and *Cryptotis parva* contain a granular segment similar to that described in *Blarina* and *Neomys*. No granular segments were found in *Sorex alpinus*, *S. cinereus*, *S. bendirii* and *S. araneus* [95]. Intriguingly, the common shrew, despite the absence of this granular segment in its glands, has been recently proven to be venomous, as its saliva produces haemolysis in frogs’ erythrocytes [7]. Thus, it is likely that granule-filled cells are not the only source of venom that can lead to discovery of more venomous shrew species in the future. Also, the European mole (*Talpa europaea*) is known to have enlarged and granular submandibular glands [4], no toxicological studies on its saliva have been performed so far. In contrast, no granular segments were found in the eastern mole (*Scalopus aquaticus*) and the shrew mole (*Neurotrichus gibbsii*). Similarly, Pacific marten (*Martes caurina*), the white-footed mouse (*Peromyscus leucopus*), the eastern meadow vole (*Microtus pennsylvanicus*), the woodland vole (*Pitimys pinetorum*), the common muskrat (*Ondatra zibethicus*), and albino house mouse (*Mus musculus*) do not possess such enlarged and granular glands [95]. This indicates that the submandibular glands with a conspicuous segment containing granule-filled cells seem to be unique to the Eulipotyphla, a group of mammals where most venomous and putatively venomous species have been discovered thus far [3]. However, as shown in the common shrew example, possession of enlarged and granular glands is not obligatory to the production of toxic saliva.

While the morphology of submandibular glands of venomous eulipotyphlans is similar, the venom delivery apparatus (grooved teeth connected to the glands) varies considerably between species, as well as compared to those of other venomous animal taxa (e.g. snakes). Solenodons have enlarged caniniform second lower incisors with a deep tubular channel on the anterolingual surface that widens and opens at the base of tooth [3]. These channelled teeth enable administration of venom into the prey’s body. In contrast, in venomous shrews such as *Blarina* and *Neomys* the enlarged first lower incisors act as a venom delivery apparatus. However, they are not channelled but have concave inner surfaces along the lingual side [94]. These elongated and forward-facing incisors form a concave trough [3], which is used to administer venom into the body of prey. A similar shallow groove in the first lower incisors is also present in *S. araneus* and some presumably venomous shrews (e.g. *Crocidula canariensis*) [3].

As mentioned above, the submandibular glands are one of the few glands (apart from the parotids, sublingual glands, and hundreds of minor salivary glands that underlie all the oral mucosa) that form the salivary system in mammals [65]. Thus, in venomous eulipotyphlans they play two basic roles: they produce and secrete saliva and produce venom. Both, functions of saliva and venom, are realized by many proteins (recruited to act as toxins in venom) produced and stored in those glands [65]. Interestingly, Caswell et al. [13] observed distinct protein profiles between the collected saliva and venom of solenodon. The majority (10 proteins) of venom proteins detected were also found in saliva, although solenodon saliva contained additional 48 proteins with diverse functional annotations. This may be indicative of spatial separation of gland functions. Spatial differentiation of snake toxins has been recently revealed in the cobra venom system [67]. This requires further investigation, to determine whether a similar spatial separation of toxins and gland functions occurs in shrews’ submandibular glands.

Mammalian saliva has cleansing, lubricating, demulcent, and antimicrobial actions, and additionally mediates taste and facilitates swallowing. It is also essential for effective sucking and has digestive functions [65]. These particular functions are achieved by a large set of proteins produced in salivary glands. Many putative proteins identified by us in this work belong to the shrew salivary system and thus contribute to the saliva actions. For instance, lysozyme, due to its antimicrobial properties [76, 77], serves as protection of oral mucosa and teeth against bacteria. It has been found in saliva of many mammals such as shrews and mice [97], and was also confirmed in venom of common vampire bats (*D. rotundus*) [76]. Amylase, here found only in glands of *S. araneus*, is a major digestive enzyme. It commonly occurs in mammalian saliva including human, rodents (rats, mice, hamsters, guinea pigs, squirrels), rabbits, and other eulipotyphlans such as hedgehogs and moles [4, 65, 97, 98], where it begins the process of digestion.

Some of the proteins identified by us have been previously reported in venoms of other eulipotyphlans, vampire bats, and platypus [13, 76]. β-defensins, here detected only in *S. araneus*, have been identified in platypus venom [99]. Serum albumins, S100 proteins, Ig kappa chain, cathepsins, histones, annexins, cystatins, actin cytoplasmic were found in venom and saliva of *S. paradoxus* [13], while carbonic anhydrase 6, glutathione peroxidase, haemoglobin subunit beta, keratin, lysozyme c, and semaphorins were confirmed for venom of *D. rotundus* [76]. Kallikreins (KLKs) and bactericidal permeability-increasing (BPI) proteins were found in venoms of solenodon and vampire bats [13, 76]. KLKs were also detected in *B. brevicauda* venom [8, 10]. Glutathione peroxidase, S100 proteins and particularly KLKs are also present in submandibular glands of nonvenomous mammals (e.g., rats and mice) [51, 65, 66].

Kallikreins deserve special attention as they are serine proteases and can have diverse functions including cleaving kininogens to kinins, and thus increasing vascular permeability and lowering blood pressure [10]. As mentioned above, these proteins have been recently identified in venoms of *Blarina* and *Solenodon* [13], but are also diverse in nonvenomous mammals (e.g., rats, mice, hamsters, and even cats) [65, 66], consisting of up to 15 paralogs. In solenodon KLK1-like proteins are present in both, saliva and venom, but being much more abundant in venom. These findings indicate that KLKs are the major functional components of solenodon venom [13]. BLTX identified in *Blarina* venom also presents kallikrein-like activity [10]. Recently, more KLKs paralogs were detected in *Blarina* venom by Hanf and Chavez [8]. Thus, KLKs are also considered to be important components of shrews’ venoms that were recruited into their venoms to effectively hunt and store prey items. In turn, paralytic activity of *N. fodiens* venom is expected to be related to the presence of PLA_2_ in venom. PLA_2_s are common among mammals, but are predominantly produced in other tissues such as pancreas and kidney [100]. Thus, the presence of PLA_2_ in shrews’ submandibular glands may indicate the strong need to provide a paralytic action of venom for rapid immobilization of larger prey and gathering it in a comatose state [3, 23].

### Venom evolution in eulipotyphlans

Animal venoms have evolved at least 101 times [101]. Within the order Eulipotyphla, few shrew species and solenodons possess oral venom system, and most remain almost completely unexplored. Recent studies of solenodon genome revealed that its venom consists of multiple KLK1 paralogs which have been independently co-opted into the venoms of shrews and solenodons following their divergence during the late Cretaceous [13, 102]. Thus, eulipotyphlan venom systems and their toxins evolved at least twice via the process of convergent evolution. *KLKs* occur in many mammals, usually as single-copy orthologs, while the solenodon genome revealed the presence of at least 15 *KLK* paralogs. Few KLK copies were also independently recruited for a predatory role in *Blarina* venom [8, 10, 11] and one putative copy in venom of *S. araneus* (present study). However, in the common shrew, functional assays must be performed to confirm the pathophysiological effects of kallikreins. Similar to shrews and solenodons, hematophagous vampire bats (e.g., *D. rotundus*) also have an oral venom system consisting of the submandibular glands in which toxins are produced and sharp incisors that deliver venom in the target species [3–6]. Interestingly, KLKs have been previously identified in their venom/saliva, alongside other serine proteases activating plasminogen [76, 103]. These findings represent a fascinating example of molecular and functional venom convergence with their distant eulipotyphlan relatives [13].

Although many proteins related to stress response have been detected in saliva of nonvenomous mammals [51–53], it is intriguing that so many putative proteins linked to stress and cellular stress response are present in *S. araneus* venom, and to a lesser extent in *N. fodiens* venom. On the one hand, high energy demands and the fast pace of shrew life may lead to stress responses [34–37] and thus require efficient mechanisms to minimize stress. On the other hand, venom production and replenishment are metabolically costly [31, 32]. Therefore, allocation of energy in toxin synthesis may lead to homeostasis disruption and thus induce stress response. To cope with stress and prevent epithelial cells from damage, effective stress mechanisms should be developed in venom glands. Our findings show that some proteins can be co-opted into the shrew venom to act as anti-stress molecules. Future work is required to assess what molecular functions display these proteins in shrew venoms (especially in *N. fodiens* and *S. araneus*) and what are their evolutionary trajectories, as comparative molecular data are currently unavailable for those species.

Finally, it is intriguing to consider whether molecules present in saliva may be recruited into shrew venom to exert antimicrobial properties and thus provide protection against pathogens. This new venom function has been recently proposed by few authors [3, 101, 104], but no empirical data have been provided to confirm it. Our results show that some proteins are likely to contribute to defence against bacteria, viruses, and parasites, but they are rather the components of shrew salivary system which aims to ensure the protection of oral mucosa and teeth against microorganisms. Future molecular and microbiological research should clarify if any proteins from shrew venom glands evolved to serve antimicrobial functions or if they are simply one element of their salivary system.

## Conclusions

We report here structural and functional putative proteins in the extracts from venom glands of two shrew species: *N. fodiens* and *S. araneus*. Applying a proteomic approach coupled with GO enrichment analysis enabled us to obtain new findings on the homologous molecules that are involved in venom gland functioning. Most identified proteins participate in metabolic processes (in *N. fodiens*) and cellular stress response (*N. fodiens* and *S. araneus*). The presence of molecules involved in synthesis, cell division and differentiation may indicate high epithelial cell turnover in shrew venom glands. Well-developed protein machinery that regulates cell cycles and cell divisions is necessary to enable quick venom regeneration and thus ensure the effectiveness of venom in prey hunting and food hoarding. A large share of proteins responsible for stress reactions may be related to the high metabolic rate of shrews and costs of venom production. This result suggests that shrews require effective mechanisms to cope with various stressors (e.g., venom replenishment), also at the cell level. Some proteins may promote toxins spreading during envenomation, while others, due to their proteolytic action, are likely to reinforce venom toxicity. Finally, a vast array of proteins involved in immune response and defence against bacteria, viruses and parasites indicates a potential role of secretions from shrew venom glands in protection against pathogens. This work opens up new perspectives for studying biological activity and functions of molecules from shrews’ venom glands. Applying *de novo* peptide sequencing coupled with transcriptomic and genomic approaches and cross checking with various databases will provide new protein datasets in shrew venom/saliva and extend our knowledge on the functioning of eulipotyphlan venom systems. Although venom production is not common in mammals, the majority of existing and putative venomous mammals use oral venom systems to inject venom into target species. The methods presented here provide a promising avenue for confirming or discovering new taxa of venomous mammals.

## Materials and methods

### Shrew trapping and housing

Trapping sessions were performed in the suburbs of Poznań (western Poland) from July to September 2017. In total, we captured 10 water shrews and 10 common shrews. The captured animals were transported to laboratory and placed separately into large (39 × 21 × 28 cm; 23 l) terraria equipped with bedding (a mixture of peat, moss, and sand). Each terrarium contained a shelter (upturned clay flowerpot) and a bowl with water. Food (minced beef and live mealworms, earthworms and snails) and water were provided *ad libitum*. Shrews were kept in the animal room under controlled conditions (temperature: 21 ± 1 °C; humidity: 65–70%; artificial photoperiod: 12L:12D). After a week, they were killed using approved methods to obtain their submandibular salivary glands in which toxins are produced [7, 9].

### Extraction of shrews’ submandibular glands and sample preparation

Shrews were killed by cervical vertebrae dislocation, and their submandibular salivary (venom) glands were dissected (Fig. 1C and D) [9]. Then, 10 pairs of glands from each shrew species were transferred into 600 µl of methanol. Tissues were next homogenized, and samples were centrifuged at 10,000 × g and 4 °C for 30 min. The supernatants were collected, and the protein content was determined using a Direct Detect spectrometer (MERCK Millipore, Warsaw, Poland). Due to low protein concentration in the extracts from glands collected from a single specimen, particularly of the common shrew, all 10 pairs of glands collected from each shrew species were combined together [7, 9, 10]. Thus, two samples (one from *N. fodiens* and one from *S. araneus*) were used for the further processing.

### Protein identification

**Fig. 4:**
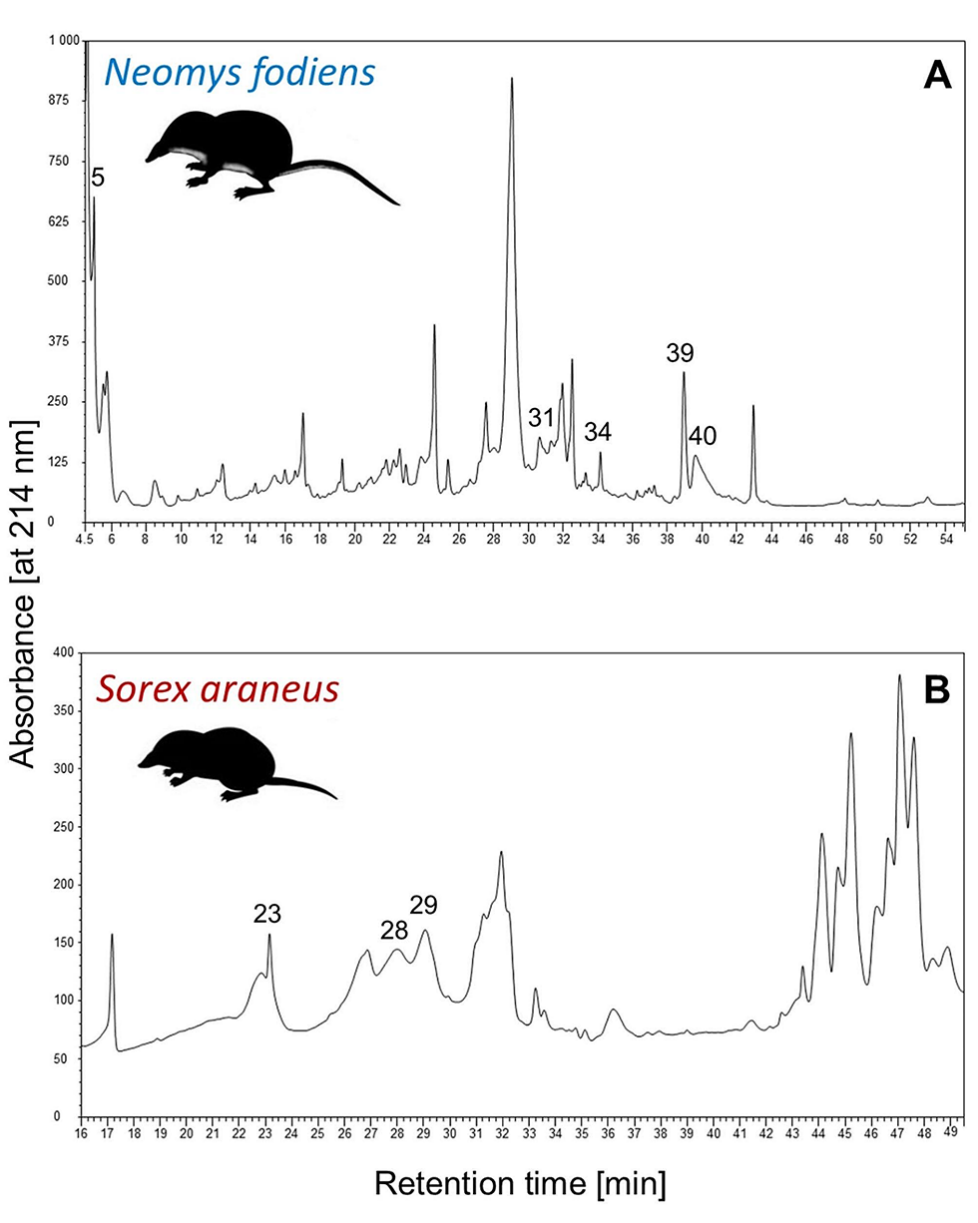
Chromatograms displaying separation of the methanolic extracts from venom glands of *Neomys fodiens* (**A**) and *Sorex araneus* (**B**). Numbers above the peaks indicate analysed fractions

Supernatants suspended in methanol were used for separation before peptide analysis by reverse phase high-performance liquid chromatography (RP-HPLC). Separation was performed using a Dionex Ultimate 3000 chromatographic system comprising a dual pump programmable solvent module. Supernatants were analysed using a BioBasic-18 analytical column (5 μm, 150 × 4.6 mm; Thermo Scientific). The samples were eluted with a gradient of 5–60% acetonitrile (ACN)/0.1% TFA with a flow rate of 0.5 ml/min for 55 min. The eluent was monitored at 214 nm, and fractions (Fig. 4) were collected into 1.5-ml tubes.

Peptides from the whole extracts and separated fractions (that were proved to be the most active [9] and with the highest protein content) of *N. fodiens* and *S. araneus* venom glands (Fig. 4) were analysed by liquid chromatography coupled to tandem mass spectrometry (LC-MS/MS) using a Nano-Acquity LC system (Waters, Milford, Massachusetts, USA) and an OrbitrapVelos mass spectrometer (Thermo Electron Corp., San Jose, CA). Before performing the analysis, the proteins were subjected to an ion-solution digestion procedure. Proteins were: (1) reduced with 50 mM TCEP for 30 min at 60 °C, (2) alkylated with 200 mM MMTA for 30 min at room temperature and (3) digested overnight with trypsin (sequencing Grade Modified Trypsin - Promega V5111). Next, the samples were applied to an RP-18 precolumn (nanoACQUITY Symmetry^®^ C18 - Waters 186,003,514) using water containing 0.1% TFA as a mobile phase and were transferred to a nano-HPLC RP-18 column (nanoACQUITY BEH C18 - Waters 186,003,545). The samples were eluted with a gradient of 0–35% acetonitrile in the presence of 0.05% formic acid with a flow rate of 250 nl/min for 180 min. The column was directly coupled to the ion source of the spectrometer working within data dependent on the MS to MS/MS switch. To ensure a lack of cross contamination from previous samples, each analysis was preceded by a blank run.

The proteins were identified by a Mascot Search (Matrix Science, London, UK) against the SwissProt and NCBInr databases. Because only a few toxins have been identified in shrew venoms so far, we also searched for homologous toxin/protein sequences detected in unrelated venomous animal taxa (toxins) and other nonvenomous mammals (nontoxic/housekeeping proteins) [7]. The search parameters were as follows: type of search: MS/MS Ion Search; enzyme specificity: trypsin; fixed methylthio modification of cysteine; variable modifications: methionine oxidation; mass values: monoisotopic; protein mass: unrestricted; peptide mass tolerance: 20 ppm; fragment mass tolerance: 0.1 D; number of missed cleavage sites allowed: 1; instrument type: HCD. Peptides with Mascot scores exceeding the threshold value of *p* < 0.05 were considered positively identified. The protein content was calculated based on the Exponentially Modified Protein Abundance Index (emPAI) [105].

### Analysis of biological functions of proteins from shrews’ venom glands

The biological functions of the identified proteins were determined by searching the UniProt database (https://www.uniprot.org/). They were compared through homology to other venomous animals (including eulipotyphlans) and nonvenomous mammals [7]. All functions were then classified into 14 categories (Additional file 2: Table A2). The percentage of proteins displaying particular functions from each category was calculated (each percentage represents here single data points). To understand the cellular processes in shrews’ venom glands, protein-protein interaction networks were built using STRING database (https://string-db.org/) [21, 106]. Predicted interactions between proteins identified in this work were analysed based on homology to the common shrew *Sorex araneus* (the only shrew species for which the genome has been sequenced). Then, Gene Ontology (GO) enrichment analysis and KEGG analysis were performed to predict in which biological processes the identified proteins are involved, and to determine their molecular functions (GO molecular function analysis). Finally, GO component analysis was performed to predict the localisation of particular proteins within the cell.

### Electronic supplementary material

Below is the link to the electronic supplementary material.

**Additional file 1: table A1**: Protein identification in the extract from venom glands of the Eurasian water shrew *Neomys fodiens* based on tandem mass spectrometry analysis. Toxins are shown in bold. Peptide sequences unique to a specific protein are marked in red

**Additional file 2: table A2**: Categories of biological functions and number of proteins identified in the extracts from venom glands of *Neomys fodiens* (NF) and *Sorex araneus* (SA) displaying particular functions. Note that because most proteins display more than one function, the total number of proteins do not sum up to 313 and 187 in NF and SA, respectively

**Additional file 3: table A3**: Biological functions of proteins identified in the extract from venom glands of the Eurasian water shrew *Neomys fodiens* based on tandem mass spectrometry analysis. Toxins are shown in bold. Function categories: 1 – Cell division & cell cycle regulation, 2 – Cell differentiation & tissue development, 3 – Cell migration, 4 – Cell structure maintenance, 5 – Cell aging & apoptosis, 6 – Signal transduction, 7 – Metabolism, 8 – Transport, 9 – Stress response, 10 – Immune response, 11 – DNA repair, 12 – Behaviour, 13 – Sensory function, 14 – unknown/not clear

**Additional file 4: table A4**: Protein identification in the extract from venom glands of the common shrew *Sorex araneus* based on tandem mass spectrometry analysis. Toxins are shown in bold. Peptide sequences unique to a specific protein are marked in red

**Additional file 5: table A5**: Biological functions of proteins identified in the extract from venom glands of the common shrew *Sorex araneus* based on tandem mass spectrometry analysis. Toxins are shown in bold. Function categories: 1 – Cell division & cell cycle regulation, 2 – Cell differentiation & tissue development, 3 – Cell migration, 4 – Cell structure maintenance, 5 – Cell aging & apoptosis, 6 – Signal transduction, 7 – Metabolism, 8 – Transport, 9 – Stress response, 10 – Immune response, 11 – DNA repair, 12 – Behaviour, 13 – Sensory function, 14 – unknown/not clear

## Data Availability

All data are available in the main text and the supplementary information files.

## Abbreviations

ACN: Acetonitrile
ADAM(s): Disintegrin and metalloproteinase domain-containing protein(s)
BLTX: *Blarina* toxin
BQTX: Toxin identified in venom of *Blarinella quadraticauda*
emPAI: The Expotentially Modified Protein Abundance Index
GO: Gene Ontology enrichment analysis
HSP(s): Heat shock protein(s)
IL-12: Interleukin-12
KEGG: Kyoto Encyclopedia of Genes and Genomes
KLK1(s): Kallikrein-1 serine protease(s)
LC-MS/MS: Liquid chromatography coupled to tandem mass spectrometry
PLA_2_: Phospholipase A_2_
RP-HPLC: Reverse phase high-performance liquid chromatography
STRING: Search Tool for the Retrieval of Interacting Genes/Proteins (database)
TRPV6: Transient receptor potential of vanilloid type 6 calcium channel
