# Supplementary material for: Proteins from shrews’ venom glands play a role in gland functioning and venom production
Source: Zoological Lett. 2024 Jul 15;10:12. doi: 10.1186/s40851-024-00236-x (PMC11251227; doi:10.1186/s40851-024-00236-x)
Supplement: Supplementary file 1 — Additional file 1: table A1: Protein identification in the extract from venom glands of the Eurasian water shrew Neomys fodiens based on tandem mass spectrometry analysis. Toxins are shown in bold. Peptide sequences unique to a specific protein are marked in red [file 40851_2024_236_MOESM1_ESM.pdf]

Table A1 Protein identification in the extract from venom glands of the Eurasian water shrew *Neomys fodiens* based on tandem mass spectrometry analysis. Toxins are shown in bold. Peptide sequences unique to a specific protein are marked in red.

Accession code	Matched peptides	Protein sequence coverage [%]	Ion score	m/z	emPAI	Identified peptides	Protein name	Protein label	Species
						**whole extract**			
Q9D6P8	25	37	64	955	5.37	K.EAFSLFDK.D	Calmodulin-like protein 3	calml3	*Mus musculus*
			37	4086		R.SLGQNPTEAELQGMVNEIDKDGNGTVDFPEFLTMMSR.K + Oxidation (M)			
			57	4102		R.SLGQNPTEAELQGMVNEIDKDGNGTVDFPEFLTMMSR.K + 2 Oxidation (M)			
			97	1351		K.MKDTDSEEEIR.E			
			81	1367		K.MKDTDSEEEIR.E + Oxidation (M)			
			65	1092		K.DTDSEEEIR.E			
P49064	241	4	59	907	0.20	K.LCTVASLR.D	Serum albumin	alb	*Felis catus*
			42	751		K.NYQEAK.D			
			128	1609		K.DVFLGTFLYEYSR.R			
Q6IMF3	25	9	103	1384	0.47	K.SLNDKFASFIDK.V	Keratin, type II cytoskeletal 1	krt1	*Rattus norvegicus*
			84	1475		R.FLEQQNQVLQTK.W			
			80	1475		K.WELLQQVDTSTR.T			
			93	1265		R.TNAENEFVTIK.K			
			88	1393		R.TNAENEFVTIKK.D			
			42	1523		R.LLRDYQELMNTK.L			
Q3T140	49	51	101	1636	1.98	K.GVQGIIVVNTEGIPIK.S	Dynein light chain roadblock-type 1	dynlrb1	*Bos taurus*
			100	2439		K.STMDNPTTTQYANLMHNFILK.A			
			79	2455		K.STMDNPTTTQYANLMHNFILK.A + Oxidation (M)			
			80	1460		R.EIDPQNDLTFLR.I			
Q6P7Q4	64	33	78	1264	2.28	K.DFLLQQTMLR.I	Lactoylglutathione lyase	glo1	*Rattus norvegicus*
			67	1280		K.DFLLQQTMLR.I + Oxidation (M)			
			60	1028		K.KSLDFYTR.V			
			48	900		K.SLDFYTR.V			
			76	1962		K.FSLYFLAYEDKNDIPK.D			
			49	976		K.RFEELGVK.F			

			114	2288		K.GLAFVQDPDGYWIEILNPNK.M			
Q2KJG2	54	68	54	901	12.04	K.ITLTSDPR.L	Ubiquitin-fold modifier 1	ufm1	*Bos taurus*
			75	1587		K.VLSVPESTPFTAVLK.F			
			59	840		K.FAAEEFK.V			
			63	3575		K.FAAEEFKVPAATSAIITNDGIGIN PAQTAGNVFLK.H			
			108	2752		K.VPAATSAIITNDGIGINPAQTAGN VFLK.H			
P31044	35	6	75	1742	2.83	K.FREWHHFLVVNMK.G	Phosphatidylethanolamine-binding protein 1	pebp1	*Rattus norvegicus*
			59	1758		K.FREWHHFLVVNMK.G + Oxidation (M)			
			96	1439		R.EWHHFLVVNMK.G			
			90	1455		R.EWHHFLVVNMK.G + Oxidation (M)			
A4Z6H1	28	16	105	2209	0.56	R.AHIVFDFHQAADGIQEQQR.Q	Adenylosuccinate synthetase isozyme 2	adss	*Sus scrofa*
			140	2286		R.VGIGAFPTEQDNEIGELLQTR.G			
			84	1444		K.TLPGWNTDISNAR.T			
			79	1302		K.ELPVNAQNYVR.F			
			52	1330		R.FIEDELQIPVK.W			
Q3SZ62	28	21	87	1058	1.66	R.HYGGLTGLNK.A	Phosphoglycerate mutase 1	pgam1	*Bos taurus*
			98	1683		R.ALPFWNEEIVPQIK.E			
			49	1306		K.RVLIAAHGNSLR.G			
			78	1150		R.VLIAAHGNSLR.G			
			75	2114		K.NLKPIKPMQFLGDEETVR.K			
			55	2242		K.NLKPIKPMQFLGDEETVRK.A			
O35586	24	8	107	2233	0.27	K.AQSDGIWGEHEIDYILFLK.K	Isopentenyl-diphosphate Delta-isomerase 1	idi1	*Mesocricetus auratus*
Q9N0F1	11	5	131	1478	0.19	K.TPAFAESVTEGDVR.W	Dihydrolipoyllysine-residue succinyltransferase component of 2-oxoglutarate dehydrogenase complex, mitochondrial	dlst	*Sus scrofa*
			96	1189		K.VEGGTPLFTLR.K			
Q1ZZU7	15	15	107	1273	1.06	M.PMFVVNTNVPR.A	Macrophage migration inhibitory factor	mif	*Ovis aries*
			99	1289		M.PMFVVNTNVPR.A + Oxidation (M)			
			45	714		K.IGGAQNR.S			
Q45FY6	28	22	65	1297	1.75	K.VFIPHGLIMDR.T	Hypoxanthine-guanine phosphoribosyltransferase	hprt1	*Sus scrofa*
			56	1313		K.VFIPHGLIMDR.T + Oxidation (M)			
			66	1178		R.SIPMTVDFIR.L			

			74	1428		K.NVLIVEDIIDTGK.T			
			48	1825		R.SVGYRPDFVGFEIPDK.F			
O89106	16	30	63	1612	2.48	R.FGQHLIKPSVVFLK.T	Bis(5’-adenosyl)-triphosphatase	fhit	*Mus musculus*
			47	2365		K.HFQGTSITFSMQDGPEAGQTVK.H			
			107	2381		K.HFQGTSITFSMQDGPEAGQTVK.H + Oxidation (M)			
			75	1093		K.HVHVHVLPR.K			
Q3T0F4	8	9	127	1569	0.64	K.KAEAGAGSATEFQFR.G	40S ribosomal protein S10	rps10	*Bos taurus*
			123	1440		K.AEAGAGSATEFQFR.G			
P07107	23	22	62	1260	2.30	K.AKWDAWNELK.G	Acyl-CoA-binding protein	dbi	*Bos taurus*
			54	1060		K.WDAWNELK.G			
			46	1207		K.AYIDKVEELK.K			
Q8WNN6	18	18	114	1167	1.60	R.HVGDLGNVTAGK.D	Superoxide dismutase [Cu-Zn]	sod1	*Canis lupus familiaris*
			62	842		R.TMVVHEK.R			
			31	858		R.TMVVHEK.R + Oxidation (M)			
			42	989		R.LACGVIGIAQ.-			
O97680	19	28	71	1463	6.51	K.MIKPFFHSLSEK.Y	Thioredoxin	txn	*Bos taurus*
			59	1479		K.MIKPFFHSLSEK.Y + Oxidation (M)			
			44	1193		K.CMPTFQFFK.K			
			55	907		K.VGEFSGANK.E			
Q8K0C9	16	9	81	1028	0.46	K.GYEVHGIVR.R	GDP-mannose 4,6 dehydratase	gmds	*Mus musculus*
			79	1393		K.FYQASTSELYGK.V			
			73	909		K.VHVTVDLK.Y			
			66	947		R.VAFDELVR.E			
P10462	6	16	128	1775	0.32	K.ELPSFVGEKVDEEGLK.K	Protein S100-A2	s100a2	*Bos taurus*
Q9CR86	8	10	117	1675	0.46	K.LQAVEVVITHLAPGTK.H	Calcium-regulated heat stable protein 1	carhsp1	*Mus musculus*
Q6B345	5	11	128	1849	0.44	K.TEFLSFMNTELAAFTK.N	Protein S100-A11	s100a11	*Rattus norvegicus*
B3EWE1	9	26	69	1793	1.27	K.TYFPHFDLSPGSAQVK.G	Haemoglobin subunit alpha	hba	*Blarina brevicauda*
			96	2186		K.AVGSLDDLPGALSALSDLHAHK.L			
P27213	8	15	81	1002	1.13	R.LVSFSASHR.L	6-pyruvoyl tetrahydrobiopterin synthase	pts	*Rattus norvegicus*
			102	1456		K.VYETDNNIVVYK.G			
			89	1642		K.VYETDNNIVVYKGE.-			
Q5EA61	6	12	50	3587	0.22	K.SMTEAEQQQLIDDHFLFDKPVSPLLLASGMAR.D	Creatine kinase B-type	ckb	*Bos taurus*

			122	1848		R.LGFSEVELVQMVVDGVK.L			
Q9JJP9	4	8	59	3833	0.15	R.SMMQSLSQNPDLAAQMMLNNPLFAGNPQLQEQMR.Q	Ubiquilin-1	ubqln1	*Rattus norvegicus*
			132	1909		R.FQQQLEQLSAMGFLNR.E			
Q2KIV2	5	16	109	1789	0.76	R.FQEYHIQQNEALAAK.A	Mitochondrial import inner membrane translocase subunit Tim9	timm9	*Bos taurus*
Q5S3G4	8	9	99	1328	0.24	R.KGLDPYNILAPK.A	Cytochrome c oxidase subunit 5B, mitochondrial	cox5b	*Sus scrofa*
Q5E983	3	6	127	1603	0.18	K.SPAGLQVLNDYLADK.S	Elongation factor 1-beta	eef1b	*Bos taurus*
Q60550	6	12	74	1351	0.30	M.PPYTIVYFPVR.G	Glutathione S-transferase P	gstp1	*Mesocricetus auratus*
			87	1718		K.EAALVDMANDGVEDLR.C			
Q9QZM0	4	2	117	1936	0.06	R.FQQQLEQLNAMGFLNR.E	Ubiquilin-2	ubqln2	*Mus musculus*
P12815	6	12	64	1441	0.53	K.AGVNFSEFTGVWK.Y	Programmed cell death protein 6	pdcd6	*Mus musculus*
			79	1356		R.LSDQFHDILIR.K			
Q9D6Y7	4	5	85	1616	0.17	K.VFWENHDPTQGMR.Q	Mitochondrial peptide methionine sulfoxide reductase	msra	*Mus musculus*
Q3T054	10	19	30	959	0.67	R.HLTGEFEK.K	GTP-binding nuclear protein Ran	ran	*Bos taurus*
			57	1293		K.FNVWDTAGQEK.F			
			53	1213		K.NLQYYDISAK.S			
			69	1784		K.SNYNFEKPFLWLAR.K			
Q8CGV7	7	3	56	903	0.19	K.LLVYLQR.F	Thiamine-triphosphatase	thtpa	*Rattus norvegicus*
Q3T035	3	6	93	1210	0.22	K.LIGNMALLPIR.S	Actin-related protein 2/3 complex subunit 3	arpc3	*Bos taurus*
Q2EN75	6	15	62	730	0.80	K.ELTIGAK.L	Protein S100-A6	s100a6	*Sus scrofa*
			61	876		K.LMDDLDR.N			
Q8R481	3	2	87	2262	0.05	R.WLPAEYEDGLSLPFGWTPGK.T	Lactoperoxidase	lpo	*Mesocricetus auratus*
P17563	6	10	83	1905	0.27	R.NTGTEAPDYLATVDVDPK.S	Selenium-binding protein 1	selenbp1	*Mus musculus*
			39	1050		K.QFYPDLIR.E			
			62	1263		K.LNPNFLVDFGK.E			
			27	2546		K.LNPNFLVDFGKEPLGPALAHELR.Y			
P01139	8	6	68	1153	0.41	K.LQHSLDTALR.R	Beta-nerve growth factor	ngf	*Mus musculus*
			37	765		R.RLHSPR.V			
Q9D0J8	4	10	76	1074	0.30	K.SVEAAAELSAK.D	Parathymosin	ptms	*Mus musculus*
P00819	4	9	69	1127	0.44	K.LEYSNFSIR.Y	Acylphosphatase-2	acyp2	*Sus scrofa*

P02049	3	8	81	1300	0.21	K.VNVDDVGGEALGR.L	Haemoglobin subunit beta	hbb	*Nycticebus coucang*
B0VYY2	4	30	89	1632	0.44	K.GMNTLVGYDLVPEPK.I	Cytochrome c oxidase subunit 5A, mitochondrial	cox5a	*Nycticebus coucang*
			50	3428		K.EIYPYVIQELRPTLNELGISTPEELGLDKV.-			
Q9JLV1	4	2	106	1915	0.07	K.IDPQTGWPFFVDHNSR.T	BAG family molecular chaperone regulator 3	bag3	*Mus musculus*
P20456	4	7	58	1161	0.32	K.SLLVTELGSSR.T	Inositol monophosphatase 1	impa1	*Bos taurus*
			53	1109		K.EIQIIPLQR.D			
Q5XLD3	1	5	127	1994	0.10	R.GTGGVDTAAVGSVFDVSNADR.L	Creatine kinase M-type	ckm	*Sus scrofa*
P01867	4	8	71	4012	0.07	K.VTCVVVDVSEDDPDVQISWFVNNVEVHTAQTQTHR.E	Ig gamma-2B chain C region	igh-3	*Mus musculus*
A4FUI1	2	11	75	1784	0.28	R.IVHELNTTVPTASFAGK.I	Coiled-coil domain-containing protein 58	ccdc58	*Bos taurus*
Q3SYV4	2	3	70	1927	0.09	R.SALFAQINQGESITHALK.H	Adenylyl cyclase-associated protein 1	cap1	*Bos taurus*
Q2TBK8	7	2	41	1112	0.11	R.QLLELQKLK.R	Snurportin-1	snupn	*Bos taurus*
Q3MHL6	5	5	63	975	0.69	K.SHLMYAVR.E	TSC22 domain family protein 1	tsc22d1	*Bos taurus*
			36	991		K.SHLMYAVR.E+ Oxidation (M)			
Q5SUR0	1	2	93	2815	0.03	R.HVVFTAETHNFPTGVAPFSGATTGTGGR.I	Phosphoribosylformylglycinamidine synthase	pfas	*Mus musculus*
P01289	3	8	71	1210	0.50	K.ALYGHGQLSHK.R	Protachykinin-1	tac1	*Bos taurus*
Q2HJ98	6	6	43	851	0.40	R.FWEWGK.N	Acylpyruvase FAHD1, mitochondrial	fahd1	*Bos taurus*
			48	884		K.GLPWTLAK.S			
Q9CZ44	2	6	64	1352	0.23	R.EFVAVTGTEEDR.A	NSFL1 cofactor p47	nsfl1c	*Mus musculus*
			52	1218		R.HSGQDVHVVLK.L			
Q3ZBN5	5	2	42	1170	0.10	R.IAEAKLTSIPK.E	Asporin	aspn	*Bos taurus*
**P01211**	**6**	**9**	**27**	**1351**	**0.37**	**K.LPSLKTWETCK.E**	**Proenkephalin-A**	**penk**	***Bos taurus***
			**37**	**829**		**K.KYGGFMK.R**			
			**49**	**701**		**K.YGGFMK.R**			
			**28**	**839**		**K.RYGGFLK.R**			
Q8R1Q8	1	3	85	1925	0.08	K.AGATSEGVLANFFNSLLSK.K	Cytoplasmic dynein 1 light intermediate chain 1	dync1li1	*Mus musculus*
Q0P569	1	5	75	2893	0.08	K.TFFILHDINSDGVLDEQELEALFTK.E	Nucleobindin-1	nucb1	*Bos taurus*
Q2KJ39	3	4	51	1982	0.12	R.EELTAFLHPEEFPHMR.D	Reticulocalbin-3	rcn3	*Bos taurus*
Q4PL64	1	4	72	1005	0.20	K.LSLEEFIR.G	Neuron-specific calcium-binding protein hippocalcin	hpca	*Bos taurus*

A2VE52	1	7	66	2178	0.12	K.ESTMTLQQAEYEFLSFVR.Q	Oligoribonuclease, mitochondrial	rexo2	*Bos taurus*
P21571	1	8	65	1061	0.28	K.FEVLDKPQS.-	ATP synthase-coupling factor 6, mitochondrial	atp5j	*Rattus norvegicus*
Q3B7M5	2	5	46	1551	0.15	R.TGDTGMLPANYVEAI.-	LIM and SH3 domain protein 1	lasp1	*Bos taurus*
Q6P0K8	2	3	42<br>40	2028<br>1236	0.11	K.SAIVHLINYQDDAELATR.A<br>R.VSVELTNSLFK.H	Junction plakoglobin	jup	*Rattus norvegicus*
Q148C4	1	16	61	1089	0.81	R.AAVAIQSQFR.K	Purkinje cell protein 4	pcp4	*Bos taurus*
Q80Y14	1	5	56	946	0.29	K.DKVVVFLK.G	Glutaredoxin-related protein 5, mitochondrial	glrx5	*Mus musculus*
A2RUW1	1	5	54	1603	0.15	R.GPVYIGELPQDFLR.I	Toll-interacting protein	tollip	*Rattus norvegicus*
O97797	1	13	54	1584	0.36	K.NSPFYYDWHSLR.V	FXYD domain-containing ion transport regulator 3	fxyd3	*Sus scrofa*
Q6B4U9	1	4	52	979	0.21	K.IGHPAPNFK.A	Peroxiredoxin-1	prdx1	*Myotis lucifugus*
D0VX08	2	6	42	901	0.30	K.AAVTGLWGK.V	Haemoglobin subunit beta	hbb	*Pteropus giganteus*
G3X9C2	6	3	37	1062	0.15	K.EQCVNLLAK.K	F-box only protein 50	nccrp1	*Mus musculus*
P62077	2	13	43	1207	0.37	R.FIDTTLAITGR.F	Mitochondrial import inner membrane translocase subunit Tim8 B	timm8b	*Mus musculus*
Q3SZF0	1	11	49	1273	0.50	K.AKWEAWNLQK.G	Acyl-CoA-binding domain-containing protein 7	acbd7	*Bos taurus*
Q3T0I5	1	7	45	1195	0.20	K.GLALLEELLPK.G	Mitochondrial fission 1 protein	fis1	*Bos taurus*
Q3SZ68	1	28	44	1994	0.36	R.GTLSDEHAGVISVLAQQAAK.L	Ragulator complex protein LAMTOR5	lamtor5	*Bos taurus*
Q9CXP8	1	10	41	772	0.48	K.LEAGVER.I	Guanine nucleotide-binding protein G(I)/G(S)/G(O) subunit gamma-10	gng10	*Mus musculus*
Q3SZE2	1	20	40	2859	0.24	K.KHAHLTDTEIMTLVDETNMYEGVGR.M	Prefoldin subunit 1	pfdn1	*Bos taurus*
**P00592**	**2**	**4**	**37**	**819**	**0.20**	**R.ALWQFR.S**	**Phospholipase A2, major isoenzyme**	**pla2g1b**	***Sus scrofa***
Q9JI38	5	2	39	989	0.08	R.ENSLGSGKAK.R	tRNA pseudouridine(38/39) synthase	pus3	*Mus musculus*
Q2HJ54	2	2	36	751	0.14	R.MEDETK.R	Phosphatidylinositol transfer protein alpha isoform	pitpna	*Bos taurus*
A5PK65	1	5	37	767	0.38	K.GTVMTFL.-	D-dopachrome decarboxylase	ddt	*Bos taurus*

Q07258	1	3	35	1821	0.09	R.IELFQILRPDEHIAK.Q	Transforming growth factor beta-3	tgfb3	*Rattus norvegicus*
P07934	2	3	32	1514	0.10	K.QMLMLRMIMDGK.Y+ 3 Oxidation (M)	Phosphorylase b kinase gamma catalytic chain, skeletal muscle/heart isoform	phkg1	*Mus musculus*
O46409	1	3	33	1406	0.10	K.GSLTPYAEELKAK.I	Apolipoprotein A-IV	apoa4	*Sus scrofa*
Q2MKA5	1	2	31	1312	0.08	R.GARGGLPELSSAAK.H	Neuronal acetylcholine receptor subunit alpha-5	chrna5	*Mus musculus*
P43023	2	11	30	1120	0.32	K.VLSRSMASAAK.G	Cytochrome c oxidase subunit 6A2, mitochondrial	cox6a2	*Mus musculus*
Q9ERH6	2	3	30	1313	0.11	K.GAIEKEVVNQAR.L	Modulator of apoptosis 1	moap1	*Mus musculus*
P05689	1	5	30	1899	0.13	R.TYPRPHEYLSPSDLPK.S	Cathepsin Z	ctsz	*Bos taurus*
Q99LP6	1	3	30	712	0.19	R.LDPIGAK.F	GrpE protein homolog 1, mitochondrial	grpel1	*Mus musculus*
D4A7N1	1	5	29	1638	0.15	R.DHLHEVLLCSDLAK.A	MICOS complex subunit Mic25	chchd6	*Rattus norvegicus*
Q5SSZ7	13	17	29	859	0.07	IQHLPPR	E3 ubiquitin-protein ligase ZNRF3	znrf3	*Mus musculus*
Q28103	2	12	28	2483	0.17	R.IKERPALNAQDGIFVINPEMGR.S + Oxidation (M)	Microfibril-associated glycoprotein 3 (Fragment)	mfap3	*Bos taurus*
A6QLT2	1	2	28	1621	0.06	K.YLQAIMDSNAQSHK.I+ Oxidation (M)	Myotubularin-related protein 2	mtmr2	*Bos taurus*
Q99PW8	1	3	27	3633	0.04	R.AELASGPEYSPPLQYETAVKPTILSMPDMPPSGK.V+ 2 Oxidation (M)	Kinesin-like protein KIF17	kif17	*Mus musculus*
Q5I0K5	1	3	27	1211	0.13	R.VVSPDVDVILR.K	Mycophenolic acid acyl-glucuronide esterase, mitochondrial	abhd10	*Rattus norvegicus*
Q6PIP5	1	2	27	1804	0.06	R.IMNLTVMLDTALGKPR.E+ 2 Oxidation (M)	NudC domain-containing protein 1	nudcd1	*Mus musculus*
Q8R1S0	1	3	27	1610	0.09	-.MAARIGSMAGLLCVR.W+ Oxidation (M)	Ubiquinone biosynthesis monooxygenase COQ6, mitochondrial	coq6	*Mus musculus*
Q58DC0	1	5	27	1931	0.13	K.GPFYFIQGADPQFGLMK.A+ Oxidation (M)	Serine/threonine-protein phosphatase CPPED1	cpped1	*Bos taurus*
Q9JKF0	1	3	26	1515	0.12	K.LRQATLSVLPCLR.C	Taste receptor type 2 member 123	tas2r123	*Rattus norvegicus*
Q2V057	1	2	26	1154	0.09	K.ERSVTQLHGK.E	Probable proline dehydrogenase 2	prodh2	*Rattus norvegicus*
P01637	12	11	28	841	0.07	LVDGVPSR	Ig kappa chain V-V region T1	igkv3	*Mus musculus*

Q8VI38	9	10	27	906	0.13	RMETINK + Oxidation (M)	Globoside alpha-1,3-N-acetylgalactosaminyltransferase 1	gbgt1	*Mus musculus*
Q6DFX2	31	14	27	1355	0.09	LANEQIQNAGGLK	Anthrax toxin receptor 2	antxr2	*Mus musculus*
Q9CX56	9	10	26	811	0.08	TPRGEPR	26S proteasome non-ATPase regulatory subunit 8	psmd8	*Mus musculus*
P21981	15	20	26	874	0.15	QEDGSVLK	Protein-glutamine gamma-glutamyltransferase 2	tgm2	*Mus musculus*
Q8BV79	14	35	26	712	0.08	LEQVPK	TPR and ankyrin repeat-containing protein 1	trank1	*Mus musculus*
Q3SZP5	16	7	26	1515	0.12	EVAWNLTSIDLVR	Peroxisomal acyl-coenzyme A oxidase 1	acox1	*Bos taurus*
P46633	17	18	25	844	0.19	EILQQSK	Heat shock protein HSP 90-alpha	hsp90aa1	*Cricetulus griseus*
Q17R09	27	10	25	1361	0.03	MEDSGEDASLHR + Oxidation (M)	Pre-mRNA-splicing factor ATP-dependent RNA helicase PRP16	dxh38	*Bos taurus*
Q62924	16	18	25	822	0.12	ELLFSSK	A-kinase anchor protein 11	akap11	*Rattus norvegicus*
Q6PFY1	25	12	25	1485	0.18	QQREADLLEDIR	F-BAR and double SH3 domains protein 1	fchsd1	*Mus musculus*
Q8MKF1	12	17	25	903	0.04	LIVYLQR	Thiamine-triphosphatase	thtpa	*Bos taurus*
Q794H2	18	7	25	1613	0.06	VEEEEAPKETPEVK	Nucleosome assembly protein 1-like 3	nap113	*Mus musculus*
**Q8SQG8**	**14**	**8**	**24**	**980**	**0.24**	**HKMPLDPK + Oxidation (M)**	**Hyaluronidase-2**[a]	**hyal2**	***Bos taurus***
Q68FY1	20	8	24	1541	0.12	GVLSSPSLAFTPPIR	Nucleoporin NUP53	nup35	*Rattus norvegicus*
Q28141	9	8	24	973	0.27	RLNMATLR	ATP-dependent RNA helicase A	dhx9	*Bos taurus*
Q8BKF1	11	7	23	1156	0.21	LQETLQSLPK	DNA-directed RNA polymerase, mitochondrial	polrmt	*Mus musculus*
A1A5Q5	20	28	23	726	0.16	RLPEGR	Lysine-specific demethylase 4D	kdm4d	*Rattus norvegicus*
Q5FVR0	13	13	23	760	0.49	KNSGSLR	T-cell immunoglobulin and mucin domain-containing protein 2	timd2	*Rattus norvegicus*
P35831	30	12	23	1329	0.05	TSKPQELSAGALK	Tyrosine-protein phosphatase non-receptor type 12	ptpn12	*Mus musculus*
Q8MIT6	11	21	23	771	0.33	IGDLQAR	Rho-associated protein kinase 1 (Fragment)	rock1	*Bos taurus*
P21752	11	11	22	862	0.29	KTETQEK	Thymosin β-10	tmsb10	*Bos taurus*

Q3ZBM5	10	13	22	748	0.39	ADRMTR	Sorting nexin-5	snx5	*Bos taurus*
Q2KIE4	11	7	22	937	0.14	MSAEDIEK + Oxidation (M)	Malignant T-cell-amplified sequence 1	mcts1	*Bos taurus*
Q9R103	30	14	22	1350	0.11	SLNHSGETLHQK	Interleukin-12 subunit alpha	il12a	*Rattus norvegicus*
O08789	17	17	22	827	0.24	LAPAEEAK	Max-binding protein MNT	mnt	*Mus musculus*
Q29466	17	10	22	1158	0.39	EINTNQEALK	V-type proton ATPase 116 kDa subunit a isoform 1	atp6v0a 1	*Bos taurus*
Q1LZH0	6	10	22	877	0.36	LKEVFSR	U11/U12 small nuclear ribonucleoprotein 35 kDa protein	snrnp3 5	*Bos taurus*
Q9JLI6	7	11	22	703	0.67	FPGVER	Selenocysteine lyase	scly	*Mus musculus*
P97807	17	10	22	1284	0.03	KPVHPNDHVNK	Fumarate hydratase, mitochondrial	fh	*Mus musculus*
Q3SZ22	12	8	21	1251	0.25	FLGNAPCGHYK	39S ribosomal protein L46, mitochondrial	mrpl46	*Bos taurus*
Q3UHX0	9	16	21	822	0.08	YCHNIK	Nucleolar protein 8	nol8	*Mus musculus*
Q2KIF8	17	18	21	775	0.25	QDMAALK	Cysteine--tRNA ligase, mitochondrial	cars2	*Bos taurus*
Q99LC8	22	11	21	1414	0.43	LFPLNQEDVPDK	Translation initiation factor eIF-2B subunit alpha	eif2b1	*Mus musculus*
						**fraction 5**			
Q6IMF3	44	10	83	1384	0.91	K.SLNDKFASFIDK.V	Keratin, type II cytoskeletal 1	krt1	*Rattus norvegicus*
			66	826		K.FASFIDK.V			
			98	1475		R.FLEQQNQVLQTK.W			
			82	1475		K.WELLQQVDTSTR.T			
			51	909		K.YEDEINK.R			
			38	1065		K.YEDEINKR.T			
			77	1265		R.TNAENEFVTIK.K			
			58	1140		R.DYQELMNTK.L			
			42	1156		R.DYQELMNTK.L+ Oxidation (M)			
Q9Z331	40	19	66	826	1.65	K.FASFIDK.V	Keratin, type II cytoskeletal 6B	krt6b	*Mus musculus*
			46	731		R.LDSELR.N			
			51	909		K.YEDEINK.R			
			38	1065		K.YEDEINKR.T			
			106	1302		R.SLDLDSIIAEVK.A			
			53	1211		R.AEAESWYQTK.Y			
			79	1165		K.YEELQVTAGR.H			
			52	1114		K.LEGLEDALQK.A			
			30	987		K.QEMARLLK.E			

			42	1152		K.EYQELMNVK.L			
			57	1263		K.LALDVEIATYR.K			
			30	1121		R.KLLEGEECR.L			
			48	993		K.LLEGEECR.L			
P02769	10	8	78	1567	0.27	K.DAFLGSFLYEYSR.R	Serum albumin	alb	*Bos taurus*
			46	1438		R.RHPEYAVSVLLR.L			
			98	1479		K.LGEYGFQNALIVR.Y			
			33	1399		K.TVMENFVAFVDK.C			
H6BDU4	4	7	82	1167	0.21	R.HVGDLGNVTAGK.D	Superoxide dismutase [Cu-Zn]	sod1	*Camelus dromedarius*
Q91XV3	2	5	67	1274	0.21	K.AEGAGTEEEGTPK.E	Brain acid soluble protein 1	basp1	*Mus musculus*
P21571	3	8	54	1061	0.28	K.FEVLDKPQS.-	ATP synthase-coupling factor 6, mitochondrial	atp5j	*Rattus norvegicus*
B2RXB2	4	8	43	772	0.43	R.IEDLQR.N	Heat shock factor-binding protein 1-like protein 1	hsbp1l1	*Mus musculus*
Q2HJ54	3	2	44	751	0.14	R.MEDETK.R	Phosphatidylinositol transfer protein alpha isoform	pitpna	*Bos taurus*
P08814	1	11	34	1358	0.30	R.AAEEEDEADPKR.Q	Parathymosin	ptms	*Bos taurus*
Q9R002	3	2	30	1172	0.09	K.KQHNINYEK.G	Interferon-activable protein 202	ifi202	*Mus musculus*
Q91VH1	1	3	27	1387	0.10	R.YGLEGGCTDDSLL.-	Adiponectin receptor protein 1	adipor1	*Mus musculus*
P14841	14	8	28	1207	0.12	GTHTLTKSSCK	Cystatin-C	cst3	*Rattus norvegicus*
Q52RG8	12	12	27	852	0.09	GWGTAHPK	Fibroblast growth factor receptor substrate 3	frs3	*Rattus norvegicus*
Q5E9S2	16	10	26	1080	0.07	GEGGRFFSPK	Nuclear transcription factor Y subunit alpha	nfya	*Bos taurus*
Q8BV79	11	16	26	712	0.07	LEQVPK	TPR and ankyrin repeat-containing protein 1	trank1	*Mus musculus*
Q921F4	15	11	26	852	0.11	SSSSSSSPK	Heterogeneous nuclear ribonucleoprotein L-like	hnrnpll	*Mus musculus*
Q8SPJ1	14	18	26	811	0.01	LVQLLVK	Junction plakoglobin	jup	*Bos taurus*
A6QNR1	16	12	25	1121	0.10	MEQQEMAQK	Ribosomal RNA processing protein 36 homolog	rrp36	*Bos taurus*
Q922J3	24	20	25	901	0.23	QQLEGAEK	CAP-Gly domain-containing linker protein 1	clip1	*Mus musculus*
Q99K01	15	21	25	738	0.10	KVDHIK	Pyridoxal-dependent decarboxylase domain-containing protein 1	pdxdc1	*Mus musculus*

Q9EQH2	16	10	25	727	0.07	EKPELL	Endoplasmic reticulum aminopeptidase 1	erap1	*Mus musculus*
Q2TBI0	8	8	25	979	0.02	HFGSVDYR	Lipopolysaccharide-binding protein	lbp	*Bos taurus*
**P12067**	**6**	**8**	**24**	**930**	**0.07**	**YWCNDGK**	**Lysozyme C-1**	**lyz1**	***Sus scrofa***
P05008	9	8	24	817	0.34	EGSSLAVR	Interferon alpha-B	ifnab	*Bos taurus*
Q811I0	20	17	23	879	0.20	KQPVGHSK	ATP synthase mitochondrial F1 complex assembly factor 1	atpaf1	*Mus musculus*
Q7TNT2	19	19	23	841	0.29	EDLAGIPK	Fatty acyl-CoA reductase 2	far2	*Mus musculus*
P26954	16	13	22	911	0.23	EKIPNPSK	Interleukin-3 receptor class 2 subunit beta	csf2rb2	*Mus musculus*
**Q8SQG8**	**7**	**4**	**22**	**980**	**0.37**	**HKMPLDPK + Oxidation (M)**	**Hyaluronidase-2[a]**	**hyal2**	***Bos taurus***
O08789	17	17	22	827	0.24	LAPAEEAK	Max-binding protein MNT	mnt	*Mus musculus*
O88855	6	10	22	752	0.33	GTWSFR	Leukotriene B4 receptor 1	ltb4r	*Mus musculus*
P01252	22	9	22	1566	0.01	AAEDDEDDDVDTKK	Prothymosin alpha	ptma	*Bos taurus*
Q9EPQ8	16	9	22	1132	0.27	ENDTVMISPK	Transcription factor 20	tcf20	*Mus musculus*
Q5I043	12	8	22	1091	0.16	TLLEQFADR	Ubiquitin carboxyl-terminal hydrolase 28	usp28	*Mus musculus*
P01637	12	11	21	841	0.32	LVDGVPSR	Ig kappa chain V-V region T1	igkv3	*Mus musculus*
Q8C7V3	12	13	21	849	0.37	YSQEPVK	U3 small nucleolar RNA-associated protein 15 homolog	utp15	*Mus musculus*
P48966	16	19	21	827	0.30	MEVPPQK	M-phase inducer phosphatase 2	cdc25b	*Rattus norvegicus*
						**fraction 31**			
gi|3318722	52	36	183	2210	2.24	R.LGEHNIDVLEGNEQFINAAK.I	Chain E, Leech-Derived Tryptase Inhibitor	ldti	*Sus scrofa*
			110	2282		K.IITHPNFNGNTLDNDIMLIK.L			
			95	1044		K.LSSPATLNSR.V			
			71	841		R.VATVSLPR.S			
			108	1515		K.SSGSSYPSLLQCLK.A			
			86	1051		K.APVLSDSSCK.S			
gi|201006	16	12	114	1167	0.77	R.HVGDLGNVTAGK.D	Cu/Zn-superoxide dismutase	sod1	*Mus musculus*
			56	842		R.TMVVHEK.Q			
gi|122649	3	8	119	1230	0.21	K.VNVDDVGGEALGR.L	Haemoglobin subunit beta	hbb	*Nycticebus coucang*
gi|7949005	2	8	83	1061	0,27	K.FEVIDKPQS.-	ATP synthase-coupling factor 6, mitochondrial precursor	atp5fp	*Mus musculus*
gi|16554572	4	12	60	880	0.49	R.WYLGGSAK.G	Sodium/potassium-transporting ATPase subunit gamma isoform b	fxyd2	*Mus musculus*

gi|432104182	23	22	48	855	0.08	LAISSLPR	F-box/WD repeat-containing protein 10	fbxw10	*Myotis davidii*
**gi|521028001**	**14**	**11**	**47**	**1152**	**0.11**	**KDIEFYIPK**	**Hyaluronidase PH-20[a]**	**hyal3**	***Myotis brandtii***
gi|655846629	13	15	47	827	0.12	VGTAEPR	C2 calcium-dependent domain-containing protein 4D-like, partial	c2cd4d	*Oryctolagus cuniculus*
gi|37675525	15	18	44	798	0.14	VPEVDIK	AHNAK, partial	ahnak	*Mus musculus*
gi|226437589	47	16	42	1803	0.01	TPEEEPLNLEGLVAHR	Tensin 1 isoform a	tns1	*Mus musculus*
gi|759101819	13	13	41	757	0.48	KSPADLK	Melanoma-associated antigen F1	magef1	*Pteropus vampyrus*
gi|528769852	21	15	41	874	0.56	ELQMPNK + Oxidation (M)	Solute carrier organic anion transporter family member 2B1	slco2b1	*Camelus ferus*
gi|14318722	26	9	38	1410	0.16	GAVDAAVPTNIIAAK	ATPase, H+ transporting, lysosomal V1 subunit H	atp6v1 g1	*Mus musculus*
gi|359322085	31	15	36	1216	0.03	IPPSIPPGVPSR	Dynamin-2 isoform X11	dnm2	*Canis lupus familiaris*
gi|528766190	6	10	34	752	0.54	MKCGLR	Proteasome-associated protein ECM29-like protein	ecpas	*Camelus ferus*
gi|1196614	59	15	33	1961	0.36	QVQLQIPGAELVKPGASVK	Immunoglobulin heavy chain, partial	igh	*Mus musculus domesticus*
gi|50054054	18	8	32	1249	0.05	KKPLVDQMFK + Oxidation (M)	Follistatin-related protein 5 precursor	fstl5	*Mus musculus*
gi|830220972	18	12	32	1000	0.83	VDTGGVQVAR	GTP-binding protein Rheb	rheb	*Condylura cristata*
gi|884944376	14	12	32	1277	0.42	LSLAQLENLCK	Exportin-7 isoform X1	xpo7	*Cavia porcellus*
gi|987936065	7	8	31	776	0.25	LEFPSGK	Protein APCDD1-like	apcdd1l	*Myotis davidii*
gi|852790143	18	18	31	849	0.54	VPTAPIPR	Collagen alpha-3(IX) chain	col9a3	*Dipodomys ordii*
gi|585682264	11	9	30	1075	0.87	MMLTNQNPK	Centrosomal protein of 162 kDa	cep162	*Elephantulus edwardii*
gi|432105468	11	8	30	1000	0.60	VPKTAENSR	Peptidyl-prolyl cis-trans isomerase A	ppia	*Myotis davidii*
gi|521022217	29	15	30	1235	0.36	SKWHIPVPSGK	Transmembrane protein 87A	tmem8 7a	*Myotis brandtii*
gi|194206109	23	17	30	942	0.89	SLIPAEGTR	RNA-binding protein 34	rbm34	*Equus caballus*
						**fraction 34**			
gi|3318722	48	36	177<br>93<br>77	2210<br>1044<br>841	2.24	R.LGEHNIDVLEGNEQFINAAK.I<br>K.LSSPATLNSR.V<br>R.VATVSLPR.S	Chain E, Leech-Derived Tryptase Inhibitor	ldti1	*Sus scrofa*

			108	1515		K.SSGSSYPSLLQCLK.A			
			98	1051		K.APVLSDSSCK.S			
gi|505855613	27	39	186	2062	3.21	R.QSSGGPVDTGPEYQQELDR.E	ATP synthase-coupling factor 6, mitochondrial	atp5fp	*Sorex araneus*
			79	1787		K.ADMNTFPNFTFEEPK.F			
			97	1803		K.ADMNTFPNFTFEEPK.F + Oxidation (M)			
			87	1061		K.FEVIDKPQS.-			
gi|201006	10	12	120	1167	0.77	R.HVGDLGNVTAGK.D	Cu/Zn-superoxide dismutase	sod1	*Mus musculus*
			53	842		R.TMVVHEK.Q			
gi|33087199	13	25	48	764	0.06	SSVVFVK	Lipoprotein lipase, partial	lpl	*Sus scrofa*
gi|548454268	48	18	48	1699	0.004	TKPADEEMLFIYSR	Acyl-CoA-binding protein	acbp	*Capra hircus*
gi|115270960	13	11	46	989	0.17	TTTWNDPR	BAG family molecular chaperone regulator 3	bag3	*Mus musculus*
gi|432104182	27	26	45	855	0.14	LAISSLPR	F-box/WD repeat-containing protein 10	fbxw10	*Myotis davidii*
gi|594059187	16	16	44	1008	0.02	ELQHWLAI	Sodium-dependent noradrenaline transporter isoform X3	slc6a2	*Bubalus bubalis*
gi|57094432	31	20	42	1061	0.30	EATAEDGELK	Transcription initiation factor TFIID subunit 11	taf11	*Canis lupus familiaris*
gi|560905974	9	10	39	788	0.09	IVDTLTK	BPI fold-containing family A member 2	bpifa2	*Camelus ferus*
gi|674052932	10	10	39	875	0.67	EDLDSLGK	Leucine-rich repeat-containing G-protein coupled receptor 5 isoform X1	lgr5	*Nannospalax galili*
gi|505853959	19	7	39	1535	0.38	LSEDSGVSTNVSVNK	Protein Mis18-alpha	mis18a	*Sorex araneus*
gi|27356782	15	14	39	868	0.09	VIPELNGK	Glyceraldehyde-3-phosphate dehydrogenase	gapdh	*Meriones unguiculatus*
gi|28175136	19	13	38	1179	0.17	LLAVIEEQHK	Slc38a10 protein, partial	slc38a10	*Mus musculus*
gi|545557338	16	20	36	714	0.73	LASLVGR	Odorant-binding protein-like	obp	*Canis lupus familiaris*
gi|852790143	30	30	36	849	0.40	VPTAPIPR	Collagen alpha-3(IX) chain	col9a3	*Dipodomys ordii*
gi|505775335	44	15	36	1262	0.34	NAEKSTGGGGIGSK	DnaJ homolog subfamily C member 2 isoform X1	dnajc2	*Sorex araneus*
gi|836714455	49	16	35	1787	0.59	EVSIEERLGALDIDTK	WD repeat-containing protein 43	wdr43	*Sorex araneus*
gi|731505870	14	16	34	812	0.14	DEVPEPK	Microtubule-associated protein 1A	map1a	*Loxodonta africana*

gi|830220972	17	11	32	1000	0.86	VDTGGVQVAR	GTP-binding protein Rheb	rheb	*Condylura cristata*
gi|27658051	27	17	30	1042	0.96	LEAAGVAEQR	MHC class I antigen	mhc	*Equus caballus*
						**fraction 39**			
Q6IMF3	61	10	98	1384	1.17	K.SLNDKFASFIDK.V	Keratin, type II cytoskeletal 1	krt1	*Rattus norvegicus*
			71	826		K.FASFIDK.V			
			97	1475		R.FLEQQNQVLQTK.W			
			86	1475		K.WELLQQVDTSTR.T			
			36	909		K.YEDEINK.R			
			65	1065		K.YEDEINKR.T			
			40	1421		K.RTNAENEFVTIK.K			
			81	1265		R.TNAENEFVTIK.K			
			96	1393		R.TNAENEFVTIKK.D			
			36	1523		R.LLRDYQELMNTK.L			
			58	1140		R.DYQELMNTK.L			
			46	1156		R.DYQELMNTK.L+ Oxidation (M)			
A1L595	42	24	63	1061	2.06	K.ATMQNLNDR.L	Keratin, type I cytoskeletal 17	krt17	*Bos taurus*
			70	808		R.LASYLDK.V			
			50	745		K.TIEDLR.N			
			71	806		R.LAADDFR.T			
			77	1028		R.VLDELTLAR.A			
			53	774		R.ILNEMR.D			
			47	1140		R.DQYEKMAEK.N			
			53	1143		K.DAEDWFFSK.T			
			99	1361		R.EVATNSELVQSGK.S			
			45	832		K.SEISELR.R			
			70	1379		K.TRLEQEIATYR.R			
			78	1121		R.LEQEIATYR.R			
			33	1516		R.LLEGEDAHLTQYK.T			
Q3T140	87	83	50	2220	78.02	R.LQSQKGVQGIIVVNTEGIPIK.S	Dynein light chain roadblock-type 1	dynlrb1	*Bos taurus*
			105	1636		K.GVQGIIVVNTEGIPIK.S			
			111	2439		K.STMDNPTTTQYANLMHNFILK.A			
			39	2455		K.STMDNPTTTQYANLMHNFILK.A + Oxidation (M)			
			77	1903		R.STVREIDPQNDLTFLR.I			
			85	1460		R.EIDPQNDLTFLR.I			
			92	1143		K.KNEIMVAPDK.D			
			77	1015		K.NEIMVAPDK.D			
			57	1031		K.NEIMVAPDK.D + Oxidation (M)			

			54	2448		K.NEIMVAPDKDYFLIVIQNPTE.-			
			47	2464		K.NEIMVAPDKDYFLIVIQNPTE.- + Oxidation (M)			
			44	1451		K.DYFLIVIQNPTE.-			
P18203	19	25	78	1314	2,52	M.GVQVETISPGDGR.T	Peptidyl-prolyl cis-trans isomerase FKBP1A	fkbp1a	*Bos taurus*
			105	1533		R.GWEEGVAQMSVGQR.A			
			71	1549		R.GWEEGVAQMSVGQR.A + Oxidation (M)			
Q3ZBZ8	18	8	86	1889	0.29	R.AMADPEVQQIMSDPAMR.L	Stress-induced-phosphoprotein 1	stip1	*Bos taurus*
			71	1001		R.LILEQMQK.D			
			80	1136		K.DPQALSEHLK.N			
			75	1100		K.LMDVGLIAIR.-			
P54149	15	10	92	1166	0.44	K.IVSPQEALPGR.K	Mitochondrial peptide methionine sulfoxide reductase	msra	*Bos taurus*
			86	1616		K.VFWENHDPTQGMR.Q			
Q9N0F1	9	5	127	1478	0.19	K.TPAFAESVTEGDVR.W	Dihydrolipoyllysine-residue succinyltransferase component of 2-oxoglutarate dehydrogenase complex, mitochondrial	dlst	*Sus scrofa*
			87	1189		K.VEGGTPLFTLR.K			
Q3YIX4	8	10	123	1949	0.16	K.GNDISSGTVLSDYVGSGPPK.G	Phosphatidylethanolamine-binding protein 1	pebp1	*Canis lupus familiaris*
Q6P7Q4	17	25	78	1264	1.44	K.DFLLQQTMLR.I	Lactoylglutathione lyase	glo1	*Rattus norvegicus*
			54	1028		K.KSLDFYTR.V			
			44	900		K.SLDFYTR.V			
			57	976		K.RFEELGVK.F			
			65	2288		K.GLAFVQDPDGYWIEILNPNK.M			
H6BDU4	6	7	106	1167	0.30	R.HVGDLGNVTAGK.D	Superoxide dismutase [Cu-Zn]	sod1	*Camelus dromedarius*
Q921H9	6	4	91	1177	0.17	R.LVDYLEGIQK.N	Cytochrome c oxidase assembly factor 7	coa7	*Mus musculus*
Q6X9Z5	5	10	76	1256	0.29	K.NIEDVIAQGIGK.L	60S acidic ribosomal protein P2	prlp2	*Equus caballus*
Q3SZ68	5	21	73	1994	0.36	R.GTLSDEHAGVISVLAQQAAK.L	Ragulator complex protein LAMTOR5	lamtor5	*Bos taurus*
Q2NKV2	5	13	76	1229	0.41	R.ILDLIDDAWR.E	Anaphase-promoting complex subunit 13	anapc13	*Bos taurus*
Q3T0E0	5	11	81	833	0.47	K.AVSYLGPK.-	Copper transport protein ATOX1	atox1	*Bos taurus*

P02049	5	15	102	1300	0.47	K.VNVDDVGGEALGR.L	Haemoglobin subunit beta	hbb	*Nycticebus coucang*
			41	1274		R.LLVVYPWTQR.F			
Q0P569	4	2	91	1272	0.08	R.DLELLIQTATR.D	Nucleobindin-1	nucb1	*Bos taurus*
Q3T0Y8	9	17	51	1103	1.05	K.NIMTQNVER.I	Vesicle-associated membrane protein 8	vamp8	*Bos taurus*
			49	952		R.GENLDHLR.N			
Q5E983	2	6	119	1603	0.18	K.SPAGLQVLNDYLADK.S	Elongation factor 1-beta	eef1b	*Bos taurus*
Q9EQX9	2	7	91	1188	0.27	K.SNEAQAIETAR.A	Ubiquitin-conjugating enzyme E2 N	ube2n	*Rattus norvegicus*
Q91XV3	3	9	55	937	0.46	K.GYNVNDEK.A	Brain acid soluble protein 1	basp1	*Mus musculus*
			79	1274		K.AEGAGTEEEGTPK.E			
P62077	3	21	65	1207	0.87	R.FIDTTLAITGR.F	Mitochondrial import inner membrane translocase subunit Tim8 B	timm8b	*Mus musculus*
			56	832		R.FAQIVQK.G			
Q0PGG4	3	4	80	975	0.22	K.AGFAGDDAPR.A	Actin, cytoplasmic 1	actb	*Bos mutus grunniensis*
			37	997		R.DLTDYLMK.I			
B3EWE1	2	6	66	998	0.32	R.TFASFPTTK.T	Haemoglobin subunit alpha	hba	*Blarina brevicauda*
B0VYY2	2	10	50	770	0.44	K.IIDAALR.A	Cytochrome c oxidase subunit 5A, mitochondrial	cox5a	*Nycticebus coucang*
			62	991		R.LNDFASAVR.I			
P11751	2	10	57	1492	0.23	K.VGGQAGDYGAEALER.M	Haemoglobin subunit alpha	hba	*Megaderma lyra*
Q8SPJ1	5	4	30	1352	0.23	R.TMQNTSDLDTAR.C	Junction plakoglobin	jup	*Bos taurus*
			37	743		R.LADGLQK.M			
			31	846		R.NLSDVATK.Q			
			51	1001		K.QEGLESVLK.I			
Q8WN94	1	9	67	1060	0.51	K.WDAWNELK.G	Acyl-CoA-binding protein	dbi	*Oryctolagus cuniculus*
Q9JLV1	2	3	60	800	0.15	K.VEAILEK.V	BAG family molecular chaperone regulator 3	bag3	*Mus musculus*
			30	1413		K.ELLALDSVDPEGR.A			
A4FUI1	2	11	37	1784	0.28	R.IVHELNTTVPTASFAGK.I	Coiled-coil domain-containing protein 58	ccdc58	*Bos taurus*
Q9ESM2	8	2	35	798	0.11	K.VEPGELR.E	Hyaluronan and proteoglycan link protein 2	hapln2	*Rattus norvegicus*
Q1LZ95	1	2	50	769	0.17	K.LLLQQR.S	Isopentenyl-diphosphate Delta-isomerase 1	idi1	*Bos taurus*
Q28895	2	4	44	887	0.29	K.TYSYLNK.L	Epididymal secretory protein E1	npc2	*Canis lupus familiaris*
Q3T087	1	3	44	830	0.23	K.AEEILEK.G	60S ribosomal protein L11	rpl11	*Bos taurus*
P21571	2	8	41	1061	0.28	K.FEVLDKPQS.-	ATP synthase-coupling factor 6, mitochondrial	atp5j	*Rattus norvegicus*

Q5M827	2	6	29<br>37	1264<br>1096	0.30	K.MVEPQYQELK.S<br>R.TPTLYLDFK.L	Pirin	pir	*Rattus norvegicus*
B5DF11	1	3	37	916	0.19	R.EDKITSPK.T	AN1-type zinc finger protein 5	zfand5	*Rattus norvegicus*
A8WCF8	9	3	33	798	0.15	R.QGAGTPLR.Q	Tumor protein p63-regulated gene 1-like protein	tprg1l	*Rattus norvegicus*
Q5EB81	4	2	35	825	0.13	K.KSPPELR.V	NADH-cytochrome b5 reductase 1	cyb5r1	*Rattus norvegicus*
Q5PPH4	1	2	36	851	0.14	R.DLAQHLR.T	Zinc finger protein 414	znf414	*Rattus norvegicus*
Q64176	1	2	33	1394	0.07	K.LDQMTAMSLLKK.S+ Oxidation (M)	Carboxylesterase 1E	ces1e	*Mus musculus*
P37089	1	2	33	1522	0.05	K.REEQGLGPEPSAPR.Q	Amiloride-sensitive sodium channel subunit alpha	scnn1a	*Rattus norvegicus*
A5D7J5	3	2	33	798	0.16	K.WVPEIR.C	Rho-related GTP-binding protein RhoU	rhou	*Bos taurus*
O77559	1	4	32	867	0.22	R.SLPEPGLR.R	ADM	adm	*Canis lupus familiaris*
O54939	1	3	31	1266	0.13	K.TADEFVKESLK.Y	Testosterone 17-beta-dehydrogenase 3	hsd17b3	*Rattus norvegicus*
Q9D9V7	5	1	30<br>29	1098<br>744	0.06	R.VDKLESSPPK.Q<br>K.EVTLQR.A	Protein DENND6B	dennd6b	*Mus musculus*
**P00592**	**1**	**4**	**31**	**819**	**0.20**	**R.ALWQFR.S**	**Phospholipase A2, major isoenzyme**	**pla2g1b**	***Sus scrofa***
Q8BGQ6	1	2	29	1201	0.08	K.LNEELLSKQK.Q	EF-hand calcium-binding domain-containing protein 14	efcab14	*Mus musculus*
P09809	1	4	28	1280	0.15	K.EGGGASLAEYQAK.A	Apolipoprotein A-I	apoa1	*Oryctolagus cuniculus*
Q00PI9	1	2	28	1750	0.05	R.RYNVLGAETVLTQMR.M	Heterogeneous nuclear ribonucleoprotein U-like protein 2	hnrnpul2	*Mus musculus*
P58875	1	3	27	1784	0.09	K.IEATTLIYDCEGLGLK.H	SEC14-like protein 2	sec14l2	*Bos taurus*
P35433	13	12	29	931	0.11	ELEESGIR	Amidophosphoribosyltransferase	ppat	*Rattus norvegicus*
Q62924	20	22	28	822	0.06	ELLFSSK	A-kinase anchor protein 11	akap11	*Rattus norvegicus*
**Q9Z0F8**	**12**	**9**	**27**	**1095**	**0.08**	**SEDIKDFSR**	**Disintegrin and metalloproteinase domain-containing protein 17**	**adam17**	***Mus musculus***
Q9QYI6	28	10	27	1796	0.07	EIAEAYETLSDANSRK	DnaJ homolog subfamily B member 9	dnajb9	*Mus musculus*

Q920B9	7	14	27	705	0.15	MIDAIK + Oxidation (M)	FACT complex subunit SPT16	supt16h	*Mus musculus*
Q148N0	12	14	27	969	0.02	SWDIFFR	2-oxoglutarate dehydrogenase, mitochondrial	ogdh	*Bos taurus*
Q6P5D4	22	12	26	1215	0.05	IANLQESLLSK	Centrosomal protein of 135 kDa	cep135	*Mus musculus*
Q9JL60	15	17	26	918	0.10	EMEELLR	Glucocorticoid modulatory element-binding protein 1	gmeb1	*Mus musculus*
Q95MM9	5	8	26	772	0.12	ILESRR	Signalling lymphocytic activation molecule	slamf1	*Canis lupus familiaris*
P52552	13	10	26	1069	0.13	LVQGFQYTD	Peroxiredoxin-2 (Fragment)	prdx2	*Sus scrofa*
P46892	11	10	25	845	0.18	DIGTPSEK	Cyclin-dependent kinase 11B	cdk11b	*Rattus norvegicus*
Q9CXF4	13	12	25	825	0.11	GGLSHSLR	TBC1 domain family member 15	tbc1d1 5	*Mus musculus*
Q9EPQ8	20	14	25	1019	0.20	EAMTGRVEK	Transcription factor 20	tcf20	*Mus musculus*
Q91WR3	13	16	25	863	0.25	RMAFLAR	Activating signal cointegrator 1 complex subunit 2	ascc2	*Mus musculus*
Q9ERA5	15	10	24	1051	0.19	KEYDAVAEK	Structural maintenance of chromosomes protein 4 (Fragment)	smc4	*Microtus arvalis*
Q148E1	12	16	24	761	0.24	GCQLAPK	Apoptogenic protein 1, mitochondrial	apopt1	*Bos taurus*
Q0VCX2	13	12	24	985	0.24	LTPEEIER	78 kDa glucose-regulated protein	hspa5	*Bos taurus*
P54279	9	10	24	931	0.23	HELSYRK	Mismatch repair endonuclease PMS2	pms2	*Mus musculus*
Q9DB41	25	13	24	1165	0.15	MSSQDLSISAK	Mitochondrial glutamate carrier 2	scl25a1 8	*Mus musculus*
Q9JLF7	11	11	24	949	0.17	MLNLAFNK	Toll-like receptor 5	tlr5	*Mus musculus*
P12263	19	7	23	1427	0.23	ISALGKSAAGPLASGK	Coagulation factor VIII	f8	*Sus scrofa*
Q6DFX2	24	11	23	1355	0.25	LANEQIQNAGGLK	Anthrax toxin receptor 2	antxr2	*Mus musculus*
O08550	26	13	23	1342	0.29	TSSPLRTSPQLR	Histone-lysine N-methyltransferase 2B	kmt2b	*Mus musculus*
Q99N92	14	14	22	785	0.34	AAAALTLR	39S ribosomal protein L27, mitochondrial	mrpl27	*Mus musculus*
Q8BJS8	32	16	22	1328	0.30	AMIDIILLPSDK	Mdm2-binding protein	mtbp	*Mus musculus*
Q9MZ03	12	13	22	895	0.36	NMHPELR	ADP-ribosyl cyclase/cyclic ADP-ribose hydrolase 1	cd38	*Oryctolagus cuniculus*

Q28730	15	13	22	897	0.34	RNGPEGLR	Intercellular adhesion molecule 5	icam5	*Oryctolagus cuniculus*
Q8CG48	12	14	22	865	0.49	YEALENK	Structural maintenance of chromosomes protein 2	smc2	*Mus musculus*
Q3UMY5	18	12	21	933	0.54	KETLSSAAK	Echinoderm microtubule-associated protein-like 4	eml4	*Mus musculus*
Q5E9I1	14	8	21	1317	0.21	ITHLPTIPETVP	Cyclin-G1	pnlip	*Bos taurus*
Q8BRB7	32	11	21	1652	0.04	RPVAGERGQLLELSK	Histone acetyltransferase KAT6B	kat6b	*Mus musculus*
Q2KIE4	14	9	21	937	0.19	MSAEDIEK + Oxidation (M)	Malignant T-cell-amplified sequence 1	mcts1	*Bos taurus*
P34943	8	10	21	855	0.35	AIEVLRR	NADH dehydrogenase [ubiquinone] 1 alpha subcomplex subunit 9, mitochondrial	ndufa9	*Bos taurus*
A5PJU9	12	11	21	939	0.16	MQEMLEK + 2 Oxidation (M)	Septin-1	sept1	*Bos taurus*
						**fraction 40**			
Q6IMF3	43	10	106	1384	0.68	K.SLNDKFASFIDK.V	Keratin, type II cytoskeletal 1	krt1	*Rattus norvegicus*
			71	826		K.FASFIDK.V			
			108	1475		R.FLEQQNQVLQTK.W			
			77	1475		K.WELLQQVDTSTR.T			
			59	909		K.YEDEINK.R			
			71	1065		K.YEDEINKR.T			
			85	1265		R.TNAENEFVTIK.K			
			73	1040		R.DYQELMNTK.L			
Q9Z2T6	8	3	62	810	0.16	K.FAAFIDK.V	Keratin, type II cuticular Hb5	krt85	*Mus musculus*
			115	1277		K.LALDIEIATYR.R			
A1L595	23	14	39	1061	0.99	K.ATMQNLNDR.L	Keratin, type I cytoskeletal 17	krt17	*Bos taurus*
			71	808		R.LASYLDK.V			
			44	745		K.TIEDLR.N			
			66	806		R.LAADDFR.T			
			72	1028		R.VLDELTLAR.A			
			46	774		R.ILNEMR.D			
			94	1361		R.EVATNSELVQSGK.S			
			83	1121		R.LEQEIATYR.R			
P49065	34	7	50	734	0.35	K.GACLTPK.L	Serum albumin	alb	*Oryctolagus cuniculus*
			70	788		K.IVTDLTK.V			
			47	1715		K.ECCHGDLLECADDR.A			
			40	976		K.FLYEYSR.R			

			80	987		K.TPVSEKVTK.C			
O89106	39	36	51	1612	3.16	R.FGQHLIKPSVVFLK.T	Bis(5'-adenosyl)-triphosphatase	fhit	*Mus musculus*
			84	1149		K.TELSFALVNR.K			
			94	2365		K.HFQGTSITFSMQDGPEAGQTVK.H			
			81	2381		K.HFQGTSITFSMQDGPEAGQTVK.H + Oxidation (M)			
			70	1093		K.HVHVHVLPR.K			
Q6P7Q4	44	33	75	1264	4.94	K.DFLLQQTMLR.I	Lactoylglutathione lyase	glo1	*Rattus norvegicus*
			77	1280		K.DFLLQQTMLR.I + Oxidation (M)			
			43	1028		K.KSLDFYTR.V			
			56	900		K.SLDFYTR.V			
			104	1395		K.FSLYFLAYEDK.N			
			73	1962		K.FSLYFLAYEDKNDIPK.D			
			43	976		K.RFEELGVK.F			
			63	820		R.FEELGVK.F			
			72	2288		K.GLAFVQDPDGYWIEILNPNK.M			
Q3YIX4	20	21	91	1439	1.81	R.EWHHFLVVNMK.G	Phosphatidylethanolamine-binding protein 1	pebp1	*Canis lupus familiaris*
			30	1455		R.EWHHFLVVNMK.G + Oxidation (M)			
			153	1949		K.GNDISSGTVLSDYVGSGPPK.G			
			53	1091		K.CDEPILSNR.S			
Q9N0F1	17	7	64	788	0.29	K.DDVITVK.T	Dihydrolipoyllysine-residue succinyltransferase component of 2-oxoglutarate dehydrogenase complex, mitochondrial	dlst	*Sus scrofa*
			124	1478		K.TPAFAESVTEGDVR.W			
			80	1189		K.VEGGTPLFTLR.K			
Q3T140	15	72	93	1636	2.92	K.GVQGIIVVNTEGIPIK.S	Dynein light chain roadblock-type 1	dynlrb1	*Bos taurus*
			66	2439		K.STMDNPTTTQYANLMHNFILK.A			
			79	1460		R.EIDPQNDLTFLR.I			
			35	2448		K.NEIMVAPDKDYFLIVIQNPTE.-			
P18203	13	40	75	1314	1.13	M.GVQVETISPGDGR.T	Peptidyl-prolyl cis-trans isomerase FKBP1A	fkbp1a	*Bos taurus*
			42	1939		K.RGQTCVVHYTGMLEDGK.K			
			106	1533		R.GWEEGVAQMSVGQR.A			
B0VYY2	14	20	92	1632	0.74	K.GMNTLVGYDLVPEPK.I	Cytochrome c oxidase subunit 5A, mitochondrial	cox5a	*Nycticebus coucang*
			74	770		K.IIDAALR.A			
			86	991		R.LNDFASAVR.I			
Q9CWM4	19	54	45	906	4.58	K.AFTELQAK.V	Prefoldin subunit 1	pfdn1	*Mus musculus*

			115	1312		K.LADIQIEQLNR.T			
			37	2731		K.HAHLTDTEIMTLVDETNMYEGV			
			52	865		GR.M			
			84	1222		R.MFILQSK.E			
			32	765		K.EVIHNQLLEK.Q			
						R.EMLMAR.R + Oxidation (M)			
P54149	13	10	92	1166	0.27	K.IVSPQEALPGR.K	Mitochondrial peptide methionine sulfoxide reductase	msra	*Bos taurus*
			62	1616		K.VFWENHDPTQGMR.Q			
Q9JLV1	18	6	54	800	0.29	K.VEAILEK.V	BAG family molecular chaperone regulator	bag3	*Mus musculus*
			65	1302		K.YLMIEEYLTK.E			
			29	1318		K.YLMIEEYLTK.E + Oxidation (M)			
			98	1413		K.ELLALDSVDPEGR.A			
			55	829		K.VQTILEK.L			
Q3ZBZ8	11	5	60	1001	0.22	R.LILEQMQK.D	Stress-induced-phosphoprotein 1	stip1	*Bos taurus*
			83	1136		K.DPQALSEHLK.N			
			83	1100		K.LMDVGLIAIR.-			
H6BDU4	9	14	94	1167	0.47	R.HVGDLGNVTAGK.D	Superoxide dismutase [Cu-Zn]	sod1	*Camelus dromedarius*
			31	989		R.LACGVIGIAQ.-			
Q9CXP8	5	19	54	728	1.20	R.LVEQLK.L	Guanine nucleotide-binding protein G(I)/G(S)/G(O) subunit gamma-10	gng10	*Mus musculus*
			74	772		K.LEAGVER.I			
P02102	9	14	60	1274	0.47	R.LLVVYPWTQR.F	Haemoglobin subunit epsilon-1	hbe1	*Capra hircus*
			29	1166		K.LVSGVATALAHK.Y			
Q66HD3	9	2	46	734	0.11	K.QDTLMK.V	Nuclear autoantigenic sperm protein	nasp	*Rattus norvegicus*
			42	750		K.QDTLMK.V+ Oxidation (M)			
			29	1234		K.RMAVLLEQMK.E+ Oxidation (M)			
Q3T087	5	3	57	830	0.23	K.AEEILEK.G	60S ribosomal protein L11	rpl11	*Bos taurus*
Q921H9	2	4	80	1177	0.17	R.LVDYLEGIQK.N	Cytochrome c oxidase assembly factor 7	coa7	*Mus musculus*
Q3T0E0	1	11	77	833	0.47	K.AVSYLGPK.-	Copper transport protein ATOX1	atox1	*Bos taurus*
P62959	4	11	57	1388	0.25	K.AQVAQPGGDTIFGK.I	Histidine triad nucleotide-binding protein 1	hint1	*Rattus norvegicus*
Q8WN94	5	9	51	1060	0.35	K.WDAWNELK.G	Acyl-CoA-binding protein	dbi	*Oryctolagus cuniculus*
A4FUI1	2	11	75	1784	0.28	R.IVHELNTTVPTASFAGK.I	Coiled-coil domain-containing protein 58	ccdc58	*Bos taurus*

Q5NRP9	2	4	63	1064	0.19	R.CSCSSLMDK.E	Endothelin-1	edn1	*Atelerix albiventris*
Q9GKK4	1	4	90	1658	0.10	R.EEFGAEPELAVSAPGR.V	Galactokinase	galk1	*Canis lupus familiaris*
P00819	2	9	59	1127	0.44	K.LEYSNFSIR.Y	Acylphosphatase-2	acyp2	*Sus scrofa*
Q6PEC1	1	9	76	1108	0.38	K.AEDGENYAIK.K	Tubulin-specific chaperone A	tbca	*Rattus norvegicus*
Q2TBK8	5	2	34	1112	0.11	R.QLLELQKLK.R	Snurportin-1	snupn	*Bos taurus*
Q28895	2	4	49	887	0.29	K.TYSYLNK.L	Epididymal secretory protein E1	npc2	*Canis lupus familiaris*
Q2NKU6	2	9	49	1027	0.44	K.VDLQSLPTR.A	Protein dpy-30 homolog	dpy30	*Bos taurus*
Q1LZ95	2	2	44	769	0.17	K.LLLQQR.S	Isopentenyl-diphosphate Delta-isomerase 1	idi1	*Bos taurus*
Q0VCG3	2	14	48	1097	0.97	K.KFFQMVGLK.K	Parvalbumin alpha	pvalb	*Bos taurus*
			34	748		K.GFSPDAR.D			
Q91ZF1	3	2	39	1338	0.10	K.IPDLQSGPRSLR.M	ATP-sensitive inward rectifier potassium channel 15	kcnj15	*Rattus norvegicus*
Q1LZH0	6	2	36	877	0.15	K.LKEVFSR.Y	U11/U12 small nuclear ribonucleoprotein 35 kDa protein	snrnp35	*Bos taurus*
Q8WMS0	3	4	34	1721	0.11	K.GFATSLSIVLSTVASIR.L	UDP-galactose translocator	slc35a2	*Canis lupus familiaris*
P11751	2	10	42	1492	0.23	K.VGGQAGDYGAEALER.M	Haemoglobin subunit alpha	hba	*Megaderma lyra*
Q2KIR1	1	6	37	1988	0.14	R.HDIAFVEFDNEVQAGAAR.D	U1 small nuclear ribonucleoprotein A	snrpa	*Bos taurus*
Q3ZCH0	1	2	37	1856	0.06	R.VEAVNMAEGIIHDTETK.M	Stress-70 protein, mitochondrial	hspa9	*Bos taurus*
P00515	1	2	36	1050	0.10	R.AASAYAVGDVK.C	cAMP-dependent protein kinase type II-alpha regulatory subunit	prkar2a	*Bos taurus*
Q91ZT7	1	4	36	2322	0.08	R.ILSDSSTGLAPDSIFDTSDPER.W	Ankyrin repeat and SOCS box protein 10	asb10	*Mus musculus*
Q9JI38	1	2	36	989	0.08	R.ENSLGSGKAK.R	tRNA pseudouridine(38/39) synthase	pus3	*Mus musculus*
Q6P9Z6	3	2	36	863	0.12	K.GESLFVGR.R	Tumor-associated calcium signal transducer 2	tacstd2	*Rattus norvegicus*
P31783	1	3	36	1092	0.17	R.MSPTQKETR.L+ Oxidation (M)	T-cell surface glycoprotein CD8 alpha chain	cd8a	*Bos taurus*
Q3MKQ1	4	6	32	1002	0.31	M.ESKVEQGVK.N	Protein BEX2	bex2	*Rattus norvegicus*
A2RUW1	1	2	34	801	0.15	R.SVLEAQR.G	Toll-interacting protein	tollip	*Rattus norvegicus*

Q5FVR0	1	2	34	760	0.11	R.KNSGSLR.F	T-cell immunoglobulin and mucin domain-containing protein 2	timd2	*Rattus norvegicus*
Q6DFX2	2	2	33	1355	0.08	K.LANEQIQNAGGLK.A	Anthrax toxin receptor 2	antxr2	*Mus musculus*
Q8BH34	1	3	32	3372	0.05	K.WNMEEVVLEELQVFKHPTAILNMELSLK.Q+ 2 Oxidation (M)	Semaphorin-3D	sema3d	*Mus musculus*
Q7YRA3	1	4	31	1295	0.17	R.QHVQEASVLLGD.-	Exosome complex component RRP41	exosc4	*Bos taurus*
Q2T9U2	1	2	31	1646	0.06	K.AEVEAIMEQLKELK.Q+ Oxidation (M)	Outer dense fiber protein 2	odf2	*Bos taurus*
Q91XV3	1	5	31	1275	0.21	K.AEGAGTEEEGTPK.E	Brain acid soluble protein 1	basp1	*Mus musculus*
Q3SYZ4	3	2	30	1279	0.08	K.QMVKFAANINK.E+ Oxidation (M)	Aspartate--tRNA ligase, cytoplasmic	dars	*Bos taurus*
P50232	1	4	30	2148	0.09	K.AGSSSDLENVTPKLFPETEK.E	Synaptotagmin-4	syt4	*Rattus norvegicus*
Q3SZ62	1	2	29	767	0.16	K.HGEAQVK.I	Phosphoglycerate mutase 1	pgam1	*Bos taurus*
Q8R151	18	19	31	720	0.06	MAEKVK + Oxidation (M)	NFX1-type zinc finger-containing protein 1	znfx1	*Mus musculus*
Q3V3V9	12	15	30	786	0.07	DRLVER	Capping protein, Arp2/3 and myosin-I linker protein 2	carmil2	*Mus musculus*
P97679	12	12	30	829	0.06	LVESAALK	DNA mismatch repair protein Mlh1	mlh1	*Rattus norvegicus*
Q8HYY4	15	11	30	861	0.08	LMRAAER + Oxidation (M)	Uveal autoantigen with coiled-coil domains and ankyrin repeats protein	uaca	*Bos taurus*
P43023	2	11	29	1120	0.32	K.VLSRSMASAAK.G	Cytochrome c oxidase subunit 6A2, mitochondrial	cox6a2	*Mus musculus*
Q5EAD4	17	18	29	861	0.09	MERATVR	Short/branched chain specific acyl-CoA dehydrogenase, mitochondrial	acadsb	*Bos taurus*
P54279	7	8	29	931	0.07	HELSYRK	Mismatch repair endonuclease PMS2	pms2	*Mus musculus*
P00586	20	13	28	1048	0.12	TVSVLNGGFR	Thiosulfate sulfurtransferase	tst	*Bos taurus*
A6QM06	10	14	28	932	0.11	MELADLNK	Sterol regulatory element-binding protein cleavage-activating protein	scap	*Bos taurus*
**P14422**	**6**	**6**	**27**	**1002**	**0.13**	**FAKFLSYK**	**Phospholipase A2**	**pla2g2a**	***Oryctolagus cuniculus***
O08550	19	9	27	1342	0.10	TSSPLRTSPQLR	Histone-lysine N-methyltransferase 2B	kmt2b	*Mus musculus*

Q9D361	12	11	27	870	0.03	IPSVTLNK	U11/U12 small nuclear ribonucleoprotein 48 kDa protein	snrnp48	*Mus musculus*
Q0VCR8	25	24	27	829	0.10	LAALSSLR	Exocyst complex component 3-like protein	exoc3l1	*Bos taurus*
Q9D799	17	15	27	887	0.19	ELEAVLSK	Methionyl-tRNA formyltransferase, mitochondrial	mtfmt	*Mus musculus*
Q9DB41	15	8	26	1165	0.10	MSSQDLSISAK	Mitochondrial glutamate carrier 2	slc25a18	*Mus musculus*
Q9JLF7	10	10	26	949	0.10	MLNLAFNK	Toll-like receptor 5	tlr5	*Mus musculus*
P02680	15	13	26	988	0.14	KMVEEILK	Fibrinogen gamma chain	fgg	*Rattus norvegicus*
Q8BJS8	26	13	26	1328	0.10	AMIDIILLPSDK	Mdm2-binding protein	mtbt	*Mus musculus*
P35479	18	16	26	969	0.10	VKPQLEEK	Leukocyte cysteine proteinase inhibitor 1	plcpi	*Sus scrofa*
A4K436	11	14	26	702	0.16	KESDPK	Regulator of telomere elongation helicase 1	rtel	*Bos taurus*
Q924Y8	20	21	25	789	0.21	QAVSEEK	Probable G-protein coupled receptor 149	gpr149	*Rattus norvegicus*
Q8VI38	9	10	25	906	0.23	RMETINK + Oxidation (M)	Globoside alpha-1,3-N-acetylgalactosaminyltransferase 1	gbgt1	*Mus musculus*
Q8BQ48	20	10	25	1296	0.03	SDATVSSDNMDR	Centrosomal protein of 295 kDa	cep295	*Mus musculus*
P46892	14	12	25	845	0.22	DIGTPSEK	Cyclin-dependent kinase 11B	cdk11b	*Rattus norvegicus*
P24643	13	17	24	951	0.14	WEVDEMK + Oxidation (M)	Calnexin	canx	*Canis lupus familiaris*
Q27975	24	18	24	946	0.26	SAVEDEGLK	Heat shock 70 kDa protein 1A	hspa1a	*Bos taurus*
Q9XTA2	12	18	24	905	0.09	AFVEAQNK	Prolyl endopeptidase	prep	*Bos taurus*
Q9JJA2	15	17	24	881	0.07	LLDRLPR	Conserved oligomeric Golgi complex subunit 8	cog8	*Mus musculus*
Q99P69	15	9	23	952	0.25	MKSDISEK + Oxidation (M)	Kinetochore protein Nuf2	nuf2	*Mus musculus*
Q3MHM6	15	14	23	829	0.24	ALLSAVTR	Catenin alpha-1	ctnna1	*Bos taurus*
Q8SPJ1	9	13	23	813	0.02	MVPLLNK	Junction plakoglobin	jup	*Bos taurus*
P11708	16	12	23	992	0.33	EVGVYEAVK	Malate dehydrogenase, cytoplasmic	mdh1	*Sus scrofa*
Q8K0Z7	20	12	23	992	0.21	NGGMMAEGAR	Translational activator of cytochrome c oxidase 1	taco1	*Mus musculus*

O02810	22	17	22	1102	0.21	LAPEREFIK	Phosphatidylinositol 4-kinase beta	pi4kb	*Bos taurus*
D3ZZL9	15	14	22	737	0.17	EMESVK + Oxidation (M)	GRIP and coiled-coil domain-containing protein 2	gcc2	*Rattus norvegicus*
P61603	21	16	21	923	0.41	MAGQAFRK + Oxidation (M)	10 kDa heat shock protein, mitochondrial	hspe1	*Bos taurus*
A1XQU3	16	17	21	717	0.06	KITTAGK	60S ribosomal protein L14	rpl14	*Sus scrofa*
P83095	15	17	21	949	0.41	HYEKDMK	Serine beta-lactamase-like protein LACTB, mitochondrial	lactb	*Bos taurus*

[a]hyaluronidase is devoid of toxic activity but acts as a toxin spreading factor commonly found in animal venoms
