# Supplementary material for: Proteins from shrews’ venom glands play a role in gland functioning and venom production
Source: Zoological Lett. 2024 Jul 15;10:12. doi: 10.1186/s40851-024-00236-x (PMC11251227; doi:10.1186/s40851-024-00236-x)
Supplement: Supplementary file 2 — Additional file 2: table A2: Categories of biological functions and number of proteins identified in the extracts from venom glands of Neomys fodiens (NF) and Sorex araneus (SA) displaying particular functions. Note that because most proteins display more than one function, the total number of proteins do not sum up to 313 and 187 in NF and SA, respectively [file 40851_2024_236_MOESM2_ESM.pdf]

**Table A2** Categories of biological functions and number of proteins identified in the extracts from venom glands of *Neomys fodiens* (NF) and *Sorex araneus* (SA) displaying particular functions. Note that because most proteins display more than one function, the total number of proteins do not sum up to 313 and 187 in NF and SA, respectively.

| **Function category** | **Biological function** | **Proteins no.** | |
|---|---|---|---|
| | | NF | SA |
| 1 – Cell division & cell cycle regulation | cell division, cell cycle cytokinesis, cell proliferation, cell cycle regulation/control/progression, cell growth control, mitotic cell cycle, mitosis regulation, DNA replication | 48 | 30 |
| 2 – Cell differentiation & tissue development | cell differentiation, angiogenesis, tissue/organ development, morphogenesis | 48 | 39 |
| 3 – Cell migration | (regulation of) cell migration | 22 | 19 |
| 4 – Cell structure maintenance | cytoskeleton organisation, microtubules structure, actin filament organisation, intermediate filament cytoskeleton organisation, mitochondrial genome maintenance, Golgi organisation, membrane structure, cell shape regulation, blood vessel diameter maintenance, extracellular matrix assembly, cell adhesion, cell junction assembly | 48 | 34 |
| 5 – Cell aging & apoptosis | aging, apoptosis, programmed cell death (regulation) | 36 | 30 |
| 6 – Signal transduction | signal transduction, cell signalling (pathway), synaptic transmission | 68 | 42 |
| 7 – Metabolism | biosynthesis, anabolism, catabolism, transcription and translation regulation, glycolysis, proteolysis, protein degradation, protein homeostasis, protein folding, protein ubiquitination, phosphorylation, intracellular pH reduction, cell motility, neurotropic and neuroprotective activity, regulation of heart rate and blood pressure, muscle contraction, wound healing, protein secretion, regulation of the blood coagulation cascade | 190 | 123 |
| 8 – Transport | intra- and extracellular transport, nuclear transport, ion and electron transport, protein transport, toxin transport, membrane fission, channel or molecule transporter, microtubule-based movement, vesicle-mediated transport, chemotaxis | 66 | 41 |
| 9 – Stress response | stress response, response to hydrogen peroxide, oxidative stress, hypoxia tolerance, response to xenobiotic stimulus, proteolytic stress response, heat stress response, response to increased oxygen levels, cellular hypotonic response, cell redox homeostasis, response to cold, starvation, toxic substances, ER stress, pain, LPS, UV and X-ray secretion, cell response to nicotine | 48 | 40 |
| 10 – Immune response | innate and adaptive immune response, allergic/inflammatory/antimicrobial response, cell response to interleukins (IL), regulation of macroautophagy | 46 | 39 |
| 11 – DNA repair | DNA repair | 10 | 9 |

| | | | |
|---|---|---|---|
| 12 – Behaviour | eating behaviour, adult feeding behaviour, adult locomotory behaviour, aggressive behaviour, behavioural fear response, behavioural response to nicotine, maternal behaviour | 4 | 4 |
| 13 – Sensory function | sensory perception of pain, bitter taste, smell, and sound, visual perception, learning or memory | 6 | 7 |
| 14 – unknown/not clear | function unknown, not clear or not characterized | 7 | 2 |
