# Supplementary material for: Proteins from shrews’ venom glands play a role in gland functioning and venom production
Source: Zoological Lett. 2024 Jul 15;10:12. doi: 10.1186/s40851-024-00236-x (PMC11251227; doi:10.1186/s40851-024-00236-x)
Supplement: Supplementary file 3 — Additional file 3: table A3: Biological functions of proteins identified in the extract from venom glands of the Eurasian water shrew Neomys fodiens based on tandem mass spectrometry analysis. Toxins are shown in bold. Function categories: 1 – Cell division & cell cycle regulation, 2 – Cell differentiation & tissue development, 3 – Cell migration, 4 – Cell structure maintenance, 5 – Cell aging & apoptosis, 6 – Signal transduction, 7 – Metabolism, 8 – Transport, 9 – Stress response, 10 – Immune response, 11 – DNA repair, 12 – Behaviour, 13 – Sensory function, 14 – unknown/not clear [file 40851_2024_236_MOESM3_ESM.pdf]

Table A3 Biological functions of proteins identified in the extract from venom glands of the Eurasian water shrew *Neomys fodiens* based on tandem mass spectrometry analysis. Toxins are shown in bold. Function categories: 1 – Cell division & cell cycle regulation, 2 – Cell differentiation & tissue development, 3 – Cell migration, 4 – Cell structure maintenance, 5 – Cell aging & apoptosis, 6 – Signal transduction, 7 – Metabolism, 8 – Transport, 9 – Stress response, 10 – Immune response, 11 – DNA repair, 12 – Behaviour, 13 – Sensory function, 14 – unknown/not clear.

Accession code	Protein name	Biological function	Function category
		**whole extract**	
Q9D6P8	Calmodulin-like protein 3	translation	7
P49064	Serum albumin	cell response to starvation, maintenance of mitochondrion location, apoptosis regulation	4,5,9
Q6IMF3	Keratin, type II cytoskeletal 1	keratinization, negative regulation of inflammatory response	4,10
Q3T140	Dynein light chain roadblock-type 1	microtubule-based movement	8
Q6P7Q4	Lactoylglutathione lyase	apoptosis, transcription regulation, carbohydrate metabolism, osteoblast differentiation	2,5,7
Q2KJG2	Ubiquitin-fold modifier 1	brain development, regulation of intracellular signalling pathway, response to ER stress	2,6,9
P31044	Phosphatidylethanolamine-binding protein 1	aging, eating behaviour, signalling regulation, regulation of peptidase activity, regulation of the force of heart contraction, response to wounding, toxic substances, heat and oxidative stress	5-7,9,12
A4Z6H1	Adenylosuccinate synthetase isozyme 2	AMP biosynthesis, IMP metabolic process	7
Q3SZ62	Phosphoglycerate mutase 1	glycolysis	7
O35586	Isopentenyl-diphosphate Delta-isomerase 1	cholesterol biosynthesis	7
Q9N0F1	Dihydrolipoyllysine-residue succinyltransferase component of 2-oxoglutarate dehydrogenase complex, mitochondrial	metabolic processes	7
Q1ZZU7	Macrophage migration inhibitory factor	signalling pathway, DNA damage response, inflammatory response, apoptosis regulation, cellular senescence, prostaglandin biosynthesis	5-7,9,10
Q45FY6	Hypoxanthine-guanine phosphoribosyltransferase	GMP catabolism	7
O89106	Bis(5'-adenosyl)-triphosphatase	DNA replication, apoptosis, catabolism	1,5,7
Q3T0F4	40S ribosomal protein S10	translation	7
P07107	Acyl-CoA-binding protein	fatty acid metabolism	7
Q8WNN6	Superoxide dismutase [Cu-Zn]	stress response, removal of superoxide radicals	9
O97680	Thioredoxin	stress response, response to nitric oxide and radiation, transport, cell death regulation	5,8,9
Q8K0C9	GDP-mannose 4,6 dehydratase	biosynthesis, Notch signalling pathway	6,7
P10462	Protein S100-A2	endothelial cell migration	3
Q9CR86	Calcium-regulated heat stable protein 1	regulation of mRNA stability	7

Q6B345	Protein S100-A11	regulation of smooth muscle cell migration and cell population proliferation	1,3
B3EWE1	Haemoglobin subunit alpha	oxygen transport	8
P27213	6-pyruvoyl tetrahydrobiopterin synthase	biosynthesis	7
Q5EA61	Creatine kinase B-type	brain development, homeostasis, phosphorylation, biosynthesis	2,7
Q9JJP9	Ubiquilin-1	regulation of protein ubiquitination, response to endoplasmic reticulum stress, regulation of oxidative stress-induced intrinsic apoptotic signalling pathway, autophagosome assembly and maturation, cellular response to hypoxia, macroautophagy	5-7,9
Q2KIV2	Mitochondrial import inner membrane translocase subunit Tim9	protein transport	8
Q5S3G4	Cytochrome c oxidase subunit 5B, mitochondrial	proton transmembrane transport	8
Q5E983	Elongation factor 1-beta	translational elongation	7
Q60550	Glutathione S-transferase P	biosynthesis, metabolism	7
Q9QZM0	Ubiquilin-2	autophagosome assembly, macroautophagy, negative regulation of clathrin-dependent endocytosis	7
P12815	Programmed cell death protein 6	apoptosis, angiogenesis, cell response to heat, intracellular protein transport, neural crest cell development, endothelial cell proliferation and migration	1-3,5,8,9
Q9D6Y7	Mitochondrial peptide methionine sulfoxide reductase	aging, cell response to oxidative stress	5,9
Q3T054	GTP-binding nuclear protein Ran	actin cytoskeleton organisation, cell division, metabolism, protein transport	1,4,7,8
Q8CGV7	Thiamine-triphosphatase	dephosphorylation, metabolic processes	7
Q3T035	Actin-related protein 2/3 complex subunit 3	regulation of actin filament polymerization	4
Q2EN75	Protein S100-A6	cell response to virus	10
Q8R481	Lactoperoxidase	defence response to bacterium, response to oxidative stress	9,10
P17563	Selenium-binding protein 1	brown fat cell differentiation, protein transport	2,8
P01139	Beta-nerve growth factor	apoptosis, cell growth, signalling pathway, circadian rhythm, nerve development, axon extension, regulation of peptidase activity, sensory perception of pain, cell population differentiation, transcription regulation, protein phosphorylation, memory, adult locomotory behaviour	2,4-7,12,13
Q9D0J8	Parathymosin	apoptosis regulation, immune system process, transcription regulation	5,7,10
P00819	Acylphosphatase-2	unknown/not clear	14
P02049	Haemoglobin subunit beta	oxygen transport	8
B0VYY2	Cytochrome c oxidase subunit 5A, mitochondrial	mitochondrial electron transport	8
Q9JLV1	BAG family molecular chaperone regulator 3	autophagosome assembly, brain development, cell response to heat, mechanical stimulus, unfolded protein, muscle cell cellular homeostasis, apoptosis regulation, positive regulation of protein export from nucleus, positive regulation of protein import into nucleus, protein stabilization	2,5,7-9
P20456	Inositol monophosphatase 1	biosynthesis and metabolism, signal transduction	6,7

Q5XLD3	Creatine kinase M-type	biosynthesis, phosphorylation, response to heat	7,9
P01867	Ig gamma-2B chain C region	humoral immune response, phagocytosis	10
A4FUI1	Coiled-coil domain-containing protein 58	unknown/not clear	14
Q3SYV4	Adenylyl cyclase-associated protein 1	actin filament organization, cAMP-mediated signalling, cell morphogenesis	2,4,6
Q2TBK8	Snurportin-1	RNA binding, protein import into nucleus	7,8
Q3MHL6	TSC22 domain family protein 1	transcription regulation	7
Q5SUR0	Phosphoribosylformylglycinamidine synthase	biosynthesis and metabolism, response to xenobiotic stimulus	7,9
P01289	Protachykinin-1	regulation of cytosolic calcium ion concentration, chemical synaptic transmission, inflammatory response, response to pain, tachykinin receptors signalling pathway	6,7,9,10
Q2HJ98	Acylpyruvase FAHD1, mitochondrial	pyruvate metabolic process	7
Q9CZ44	NSFL1 cofactor p47	autophagosome assembly, Golgi organisation, membrane fusion, negative regulation of protein localization to centrosome, positive regulation of mitotic centrosome separation	1,4,7,8
Q3ZBN5	Asporin	bone and tooth mineralization, negative regulation of transforming growth factor beta receptor signalling pathway	6,7
**P01211**	**Proenkephalin-A**	**sensory perception of pain, neuropeptide signalling pathway, defence response to bacterium, locomotory behaviour, aggressive behaviour, behavioural fear response, chemical synaptic transmission**	**6,10,12,13**
Q8R1Q8	Cytoplasmic dynein 1 light intermediate chain 1	cell cycle, cell division, cellular response to nerve growth factor stimulus, microtubule cytoskeleton organization, microtubule-based movement	1,4,8
Q0P569	Nucleobindin-1	signal transduction	6
Q2KJ39	Reticulocalbin-3	biosynthesis, phospholipid homeostasis, positive regulation of peptidase activity, protein secretion and transport, regulation of protein kinase B signalling	6-8
Q4PL64	Neuron-specific calcium-binding protein hippocalcin	cellular response to calcium ion, regulation of voltage-gated calcium channel activity	7
A2VE52	Oligoribonuclease, mitochondrial	transcription	7
P21571	ATP synthase-coupling factor 6, mitochondrial	ATP metabolism, regulation of blood pressure and heart rate, ion transport, response to muscle activity	7,8
Q3B7M5	LIM and SH3 domain protein 1	ion transport	8
Q6P0K8	Junction plakoglobin	cell communication, cell migration and adhesion, Wnt signalling pathway, cell response to indole-3-methanol	3,4,6
Q148C4	Purkinje cell protein 4	calmodulin dependent kinase signalling pathway, positive regulation of neuron differentiation	2,6
Q80Y14	Glutaredoxin-related protein 5, mitochondrial	protein maturation	7

A2RUW1	Toll-interacting protein	autophagy, epithelial cell differentiation, inflammatory response, innate immune response, interleukin-1-mediated signalling pathway, phosphorylation, protein localization to endosome, signal transduction	2,6-8,10
O97797	FXYD domain-containing ion transport regulator 3	potassium and sodium ion transport	8
Q6B4U9	Peroxiredoxin-1	erythrocyte homeostasis, fibroblast proliferation, hydrogen peroxide catabolism, natural killer cell activation, natural killer cell mediated cytotoxicity, signalling pathway, removal of superoxide radicals	1,6,7,9,10
D0VX08	Haemoglobin subunit beta	oxygen transport	8
G3X9C2	F-box only protein 50	glycoprotein catabolism, cell population proliferation	1,7
P62077	Mitochondrial import inner membrane translocase subunit Tim8 B	protein transport	8
Q3SZF0	Acyl-CoA-binding domain-containing protein 7	fatty acid metabolism	7
Q3T0I5	Mitochondrial fission 1 protein	apoptosis, mitochondrial fission	5
Q3SZ68	Ragulator complex protein LAMTOR5	cell size regulation, apoptosis, signalling pathway regulation, protein localization to lysosome	4-6,8
Q9CXP8	Guanine nucleotide-binding protein G(I)/G(S)/G(O) subunit gamma-10	G protein-coupled receptor signalling pathway	6
Q3SZE2	Prefoldin subunit 1	protein folding, negative regulation of amyloid fibril formation	7
**P00592**	**Phospholipase A2, major isoenzyme**	intracellular signal transduction, biosynthesis, lipid catabolism, neutrophil chemotaxis, neutrophil mediated immunity, regulation of IL-8 production, transcription regulation, podocyte apoptosis regulation, regulation of glucose import, regulation of cell population proliferation	**1,3,5-8,10**
Q9JI38	tRNA pseudouridine(38/39) synthase	biosynthesis	7
Q2HJ54	Phosphatidylinositol transfer protein alpha isoform	lipid transport	8
A5PK65	D-dopachrome decarboxylase	melanin biosynthesis	7
Q07258	Transforming growth factor beta-3	aging, animal organ morphogenesis, signalling pathway, cell division, cell population proliferation, cell-cell junction organization, cell response to hypoxia, lung development, apoptosis regulation, regulation of macrophage cytokine production, regulation of bone mineralisation, transcription regulation, regulation of protein secretion, regulation of cell differentiation, salivary gland morphogenesis, wound healing	1,2,4-6,9,10
P07934	Phosphorylase b kinase gamma catalytic chain, skeletal muscle/heart isoform	glycogen metabolism, protein phosphorylation	7
O46409	Apolipoprotein A-IV	lipid transport and metabolism	7,8

Q2MKA5	Neuronal acetylcholine receptor subunit alpha-5	behavioural response to nicotine, cell response to nicotine, chemical synaptic transmission, detection of mechanical stimulus involved in sensory perception of pain, ion transmembrane transport, signal transduction	6,8,12,13
P43023	Cytochrome c oxidase subunit 6A2, mitochondrial	mitochondrial electron transport	8
Q9ERH6	Modulator of apoptosis 1	apoptosis, autophagy, signalling pathway	5,6
P05689	Cathepsin Z	proteolysis	7
Q99LP6	GrpE protein homolog 1, mitochondrial	protein folding	7
D4A7N1	MICOS complex subunit Mic25	DNA damage response	9
Q5SSZ7	E3 ubiquitin-protein ligase ZNRF3	limb development, protein ubiquitination, Wnt signalling pathway, catabolism, stem cell proliferation	1,2,6,7
Q28103	Microfibril-associated glycoprotein 3 (Fragment)	microfibril component	4
A6QLT2	Myotubularin-related protein 2	lipid metabolism, neuron development	2,7
Q99PW8	Kinesin-like protein KIF17	microtubule-based process, protein transport, vesicle-mediated transport	8
Q5I0K5	Mycophenolic acid acyl-glucuronide esterase, mitochondrial	catabolism	7
Q6PIP5	NudC domain-containing protein 1	unknown/not clear	14
Q8R1S0	Ubiquinone biosynthesis monooxygenase COQ6, mitochondrial	ubiquinone biosynthesis	7
Q58DC0	Serine/threonine-protein phosphatase CPPED1	cell apoptosis	5
Q9JKF0	Taste receptor type 2 member 123	detection of chemical stimulus involved in sensory perception of bitter taste	13
Q2V057	Probable proline dehydrogenase 2	proline metabolism	7
P01637	Ig kappa chain V-V region T1	adaptive immune response	10
Q8VI38	Globoside alpha-1,3-N-acetylgalactosaminyltransferase 1	carbohydrate metabolism, protein and lipid glycosylation	7
Q6DFX2	Anthrax toxin receptor 2	**toxin transport**, reproductive process	7,8
Q9CX56	26S proteasome non-ATPase regulatory subunit 8	protein catabolism	7
P21981	Protein-glutamine gamma-glutamyltransferase 2	apoptotic cell clearance, blood vessel remodelling, bone development, salivary gland morphogenesis, cell response to cocaine, dopamine and serotonin, D protein-coupled receptor signalling pathway, apoptosis regulation, cell adhesion, signal transduction, immune response regulation, positive regulation of smooth muscle cell proliferation, proteolysis	1,2,4-7,9,10
Q8BV79	TPR and ankyrin repeat-containing protein 1	unknown/not clear	14

Q3SZP5	Peroxisomal acyl-coenzyme A oxidase 1	lipid metabolism, hydrogen peroxide biosynthesis	7
P46633	Heat shock protein HSP 90-alpha	innate immune response, cell response to virus, heat, cold, apoptosis regulation, positive regulation of nitric oxide biosynthesis	5,7,9,10
Q17R09	Pre-mRNA-splicing factor ATP-dependent RNA helicase PRP16	mRNA splicing	7
Q62924	A-kinase anchor protein 11	cytoskeleton organization, protein phosphorylation, protein localization to endosome	4,7,8
Q6PFY1	F-BAR and double SH3 domains protein 1	membrane organisation, actin filament polymerisation, neuromuscular synaptic transmission	4,6
Q8MKF1	Thiamine-triphosphatase	dephosphorylation, metabolism	7
Q794H2	Nucleosome assembly protein 1-like 3	nucleosome assembly	7
**Q8SQG8**	**Hyaluronidase-2[a]**	**carbohydrate metabolism, cartilage development, cell response to IL-1, UV-B and virus, monocyte activation, aging, fibroblast migration, cell growth regulation, regulation of IL-6 and IL-8 production, signalling pathway, transcription regulation, response to reactive oxygen species**	**2-7,9,10**
Q68FY1	Nucleoporin NUP53	mRNA transport, nuclear pore organisation, protein import to nucleus, cell response to leukaemia inhibitory factor	4,7,8
Q28141	ATP-dependent RNA helicase A	DNA replication, mRNA splicing and transport, translation, transcription, fibroblast proliferation, inflammatory response, innate immune response, regulation of interferon α and β and IL-6 production, DNA repair regulation	1,7,8,10, 11
Q8BKF1	DNA-directed RNA polymerase, mitochondrial	mitochondrial transcription	7
A1A5Q5	Lysine-specific demethylase 4D	chromatin remodelling, transcription regulation, DNA damage response	1,7,9
Q5FVR0	T-cell immunoglobulin and mucin domain-containing protein 2	endocytosis, iron ion transport, phagocytosis, positive regulation of mast cell activation	7,8,10
P35831	Tyrosine-protein phosphatase non-receptor type 12	tissue regeneration, protein dephosphorylation, regulation of epidermal growth factor receptor signalling pathway	4,6,7
Q8MIT6	Rho-associated protein kinase 1 (Fragment)	actin cytoskeleton organisation, signal transduction, cell migration and maturation, cell junction assembly, protein phosphorylation	3,4,6,7
P21752	Thymosin β-10	actin filament organisation, regulation of cell migration	3,4
Q3ZBM5	Sorting nexin-5	intracellular protein transport, pinocytosis	8
Q2KIE4	Malignant T-cell-amplified sequence 1	cell cycle, DNA damage response, translation	1,7,9
Q9R103	Interleukin-12 subunit alpha	cell migration, cell population proliferation, cell response to LPS, virus, protozoan, Gram positive bacterium and UV-B, immune response, extrinsic apoptotic signalling pathway, angiogenesis, negative regulation of IL-7 production, regulation of protein secretion, cell adhesion, regulation of lymphocyte proliferation, natural killer cell activation, regulation of natural killer cell mediated cytotoxicity, T-cell activation and proliferation	1-7,9,10

O08789	Max-binding protein MNT	cellular senescence, cell cycle regulation, transcription regulation, negative regulation of apoptotic signalling pathway	1,5-7
Q29466	V-type proton ATPase 116 kDa subunit a isoform 1	pH reduction, proton transmembrane transport, vacuolar acidification	7,8
Q1LZH0	U11/U12 small nuclear ribonucleoprotein 35 kDa protein	mRNA splicing	7
Q9JLI6	Selenocysteine lyase	lipid metabolism, cell response to oxidative stress	7,9
P97807	Fumarate hydratase, mitochondrial	DNA damage response, DNA repair, metabolism, homeostasis	7,9,11
Q3SZ22	39S ribosomal protein L46, mitochondrial	structural constituent of ribosome, translation	4,7
Q3UHX0	Nucleolar protein 8	rRNA processing, protein localization to nucleus, cell response to leukaemia inhibitory factor	7,8
Q2KIF8	Cysteine--tRNA ligase, mitochondrial	translation	7
Q99LC8	Translation initiation factor eIF-2B subunit alpha	translation, oligodendrocyte development, T cell receptor signalling pathway, response to glucose, heat, peptide hormone and amino acid starvation	2,6,7,9
		**fraction 5**	
Q6IMF3	Keratin, type II cytoskeletal 1	as above	4,10
Q9Z331	Keratin, type II cytoskeletal 6B	intermediate filament organization, keratinization, epithelium morphogenesis	2,4
P02769	Serum albumin	as above	4,5,9
H6BDU4	Superoxide dismutase [Cu-Zn]	removal of superoxide radicals, stress response	9
Q91XV3	Brain acid soluble protein 1	transcription regulation, podocyte differentiation	2,7
P21571	ATP synthase-coupling factor 6, mitochondrial	as above	7,8
B2RXB2	Heat shock factor-binding protein 1-like protein 1	cellular heat acclimation	9
Q2HJ54	Phosphatidylinositol transfer protein alpha isoform	lipid transport	8
P08814	Parathymosin	immune response, regulation of apoptosis and transcription	5,7,10
Q9R002	Interferon-activable protein 202	innate immune response, inflammatory response, cellular response to interferon-beta, response to bacterium, positive regulation of interleukin-1 beta production, apoptosis regulation	5,10
Q91VH1	Adiponectin receptor protein 1	fatty acid metabolism, signalling pathway, regulation of cell growth and migration, positive regulation of cold-induced thermogenesis, glucose metabolism	3,4,6,7
P14841	Cystatin-C	apoptosis, brain and eye development, cell response hydrogen peroxide, oxidative stress, circadian sleep/wake cycle	2,5,7,9
Q52RG8	Fibroblast growth factor receptor substrate 3	fibroblast growth factor receptor signalling pathway	6
Q5E9S2	Nuclear transcription factor Y subunit alpha	transcription regulation, rhythmic process	7

Q8BV79	TPR and ankyrin repeat-containing protein 1	unknown/not clear	14
Q921F4	Heterogeneous nuclear ribonucleoprotein L-like	mRNA processing and splicing	7
Q8SPJ1	Junction plakoglobin	cell communication, cell migration and adhesion, Wnt signalling pathway, cell response to indole-3-methanol	3,4,6,7
A6QNR1	Ribosomal RNA processing protein 36 homolog	rRNA processing, ribosome biogenesis	7
Q922J3	CAP-Gly domain-containing linker protein 1	cytoplasmic microtubule organisation, protein transport into plasma membrane raft, dendrite development regulation	2,4,8
Q99K01	Pyridoxal-dependent decarboxylase domain-containing protein 1	carboxylic acid metabolism	7
Q9EQH2	Endoplasmic reticulum aminopeptidase 1	peptide catabolism, proteolysis, adaptive immune response	7,10
Q2TBI0	Lipopolysaccharide-binding protein	acute-phase response, cell response to LPS, Gram positive bacterium, innate immune response, lipid transport, LPS-mediated signalling pathway, macrophage activation	6,9,10
**P12067**	**Lysozyme C-1**	**digestion, metabolism, defence response to Gram positive bacterium, killing of cell of another organism**	**7,10**
P05008	Interferon alpha-B	adaptive immune response, B-cell proliferation and differentiation, cytokine-mediated signalling pathway, defence response to virus	2,6,10
Q811I0	ATP synthase mitochondrial F1 complex assembly factor 1	proton transport	8
Q7TNT2	Fatty acyl-CoA reductase 2	lipid biosynthesis and metabolism	7
P26954	Interleukin-3 receptor class 2 subunit beta	cytokine-mediated signalling pathway, immune response, regulation of leukocyte proliferation, protein phosphorylation	6,7,10
**Q8SQG8**	**Hyaluronidase-2[a]**	**carbohydrate metabolism, cartilage development, cell response to IL-1, UV-B and virus, hematopoietic progenitor cell differentiation**	**2,7,9,10**
O08789	Max-binding protein MNT	cellular senescence, cell cycle regulation, transcription regulation, regulation of apoptotic signalling pathway	1,5-7
O88855	Leukotriene B4 receptor 1	inflammatory response, signal transduction, neuropeptide signalling pathway	6,10
P01252	Prothymosin alpha	apoptosis, transcription	5,7
Q9EPQ8	Transcription factor 20	transcription	7
Q5I043	Ubiquitin carboxyl-terminal hydrolase 28	cell population proliferation, cell response to UV, DNA damage response, DNA repair, protein deubiquitination, response to ionizing radiation	1,7,9,11
P01637	Ig kappa chain V-V region T1	adaptive immune response	10
Q8C7V3	U3 small nucleolar RNA-associated protein 15 homolog	rRNA processing, transcription, ribosome biogenesis	7
P48966	M-phase inducer phosphatase 2	cell division, mitotic cell cycle, protein phosphorylation and dephosphorylation	1,7
		**fraction 31**	

gi|3318722	Chain E, Leech-Derived Tryptase Inhibitor	negative regulation of peptidase activity	7
gi|201006	Cu/Zn-superoxide dismutase	stress response, removal of superoxide radicals	9
gi|122649	Haemoglobin subunit beta	oxygen transport	8
gi|7949005	ATP synthase-coupling factor 6, mitochondrial precursor	ATP metabolism, ion transport, regulation of secretion, regulation of blood pressure and heart rate, response to muscle activity	7,8
gi|16554572	Sodium/potassium-transporting ATPase subunit gamma isoform b	transmembrane transport, ion homeostasis, regulation of cell population proliferation	1,7,8
gi|432104182	F-box/WD repeat-containing protein 10	protein ubiquitination	7
**gi|521028001**	**Hyaluronidase PH-20[a]**	**carbohydrate metabolism, cell adhesion**	**4,7**
gi|655846629	C2 calcium-dependent domain-containing protein 4D-like, partial	unknown/not clear	14
gi|37675525	AHNAK, partial	RNA splicing	7
gi|226437589	Tensin 1 isoform a	fibroblast migration	3
gi|759101819	Melanoma-associated antigen F1	transcription regulation, protein ubiquitination	7
gi|528769852	Solute carrier organic anion transporter family member 2B1	ion transport, transmembrane transport, liver development	2,8
gi|14318722	ATPase, H+ transporting, lysosomal V1 subunit H	response to increased oxygen levels, intracellular iron ion homeostasis, vacuolar acidification	7,9
gi|359322085	Dynamin-2 isoform X11	synaptic vesicle endocytosis, aorta development, coronary vasculature development	2,7
gi|528766190	Proteasome-associated protein ECM29-like protein	proteasome assembly	7
gi|1196614	Immunoglobulin heavy chain, partial	adaptive immune response	10
gi|50054054	Follistatin-related protein 5 precursor	cell differentiation	2
gi|830220972	GTP-binding protein Rheb	thermogenesis regulation, oligodendrocyte differentiation, TOR signalling regulation, signal transduction, pancreatic cell development	2,6,7
gi|884944376	Exportin-7 isoform X1	intracellular protein transport	8
gi|987936065	Protein APCDD1-like	regulation of Wnt signalling pathway	6
gi|852790143	Collagen alpha-3(IX) chain	bone morphogenesis, cartilage development, chondrocyte proliferation, tissue homeostasis, extracellular matrix organisation	1,2,4
gi|585682264	Centrosomal protein of 162 kDa	cilium assembly	4
gi|432105468	Peptidyl-prolyl cis-trans isomerase A	apoptosis, cell adhesion, endothelial cell activation, protein phosphorylation, leukocyte chemotaxis, response to oxidative stress, platelet activation and aggregation	3-5,7,9,10
gi|521022217	Transmembrane protein 87A	retrograde transport, endosome to Golgi	8
gi|194206109	RNA-binding protein 34	transcription	7

fraction 34

gi|3318722	Chain E, Leech-Derived Tryptase Inhibitor	as above	7
gi|505855613	ATP synthase-coupling factor 6, mitochondrial	as above	7,8
gi|201006	Cu/Zn-superoxide dismutase	as above	9
gi|33087199	Lipoprotein lipase, partial	lipid catabolism, response to glucose	7
gi|548454268	Acyl-CoA-binding protein	fatty acid metabolism	7
gi|115270960	BAG family molecular chaperone regulator 3	brain development, autophagosome assembly, cell response to heat and mechanical stimulus, extrinsic apoptotic signalling pathway, regulation of apoptosis and transcription, muscle cell cellular homeostasis, protein stabilization, regulation of protein targeting to mitochondrion	2,5-9
gi|432104182	F-box/WD repeat-containing protein 10	as above	7
gi|594059187	Sodium-dependent noradrenaline transporter isoform X3	signal transduction	6
gi|57094432	Transcription initiation factor TFIID subunit 11	transcription regulation, protein phosphorylation	7
gi|560905974	BPI fold-containing family A member 2	defence response to bacterium	10
gi|674052932	Leucine-rich repeat-containing G-protein coupled receptor 5 isoform X1	regulation of cell population proliferation and Wnt signalling pathway, hair follicle development	1,2,6
gi|505853959	Protein Mis18-alpha	cell division	1
gi|27356782	Glyceraldehyde-3-phosphate dehydrogenase	glucose metabolism, glycolysis	7
gi|28175136	Slc38a10 protein, partial	bone development, transmembrane transport, sodium ion transport	2,8
gi|545557338	Odorant-binding protein-like	sensory perception of smell	13
gi|852790143	Collagen alpha-3(IX) chain	as above	1,2,4
gi|505775335	DnaJ homolog subfamily C member 2 isoform X1	protein folding, cell apoptosis, cellular senescence, DNA replication, regulation of cell population proliferation, signal transduction, response to heat	1,5-7,9
gi|836714455	WD repeat-containing protein 43	transcription, ribosome biogenesis	7
gi|731505870	Microtubule-associated protein 1A	microtubule cytoskeleton organisation, photoreceptor cell maintenance, axonogenesis, protein transport, memory	2,4,7
gi|830220972	GTP-binding protein Rheb	as above	2,6,7
gi|27658051	MHC class I antigen	innate immune response, defence response to Gram positive bacterium	10
		**fraction 39**	
Q6IMF3	Keratin, type II cytoskeletal 1	as above	4,10
A1L595	Keratin, type I cytoskeletal 17	epithelial cell differentiation, hair follicle morphogenesis, intermediate filament organization	2,4
Q3T140	Dynein light chain roadblock-type 1	microtubule-based movement, transport	8

P18203	Peptidyl-prolyl cis-trans isomerase FKBP1A	regulation of protein ubiquitination and phosphorylation, regulation of immune response, amyloid fibril formation, cytokine-mediated signalling pathway, T-cell activation and proliferation, response to caffeine, heart morphogenesis, muscle contraction	1,2,6,9,10
Q3ZBZ8	Stress-induced-phosphoprotein 1	protein folding	7
P54149	Mitochondrial peptide methionine sulfoxide reductase	cell response to oxidative stress	9
Q9N0F1	Dihydrolipoyllysine-residue succinyltransferase component of 2-oxoglutarate dehydrogenase complex, mitochondrial	metabolic processes	7
Q3YIX4	Phosphatidylethanolamine-binding protein 1	negative regulation of peptidase activity	7
Q6P7Q4	Lactoylglutathione lyase	carbohydrate metabolism, regulation of apoptosis and transcription, osteoblast differentiation	2,5,7
H6BDU4	Superoxide dismutase [Cu-Zn]	as above	9
Q921H9	Cytochrome c oxidase assembly factor 7	mitochondrial respiratory chain	7
Q6X9Z5	60S acidic ribosomal protein P2	cytoplasmic translational elongation	7
Q3SZ68	Ragulator complex protein LAMTOR5	apoptosis regulation, cell size regulation, positive regulation of TOR signalling, protein localisation to lysosome	4-6,8
Q2NKV2	Anaphase-promoting complex subunit 13	cell cycle, cell division, protein ubiquitination	1,7
Q3T0E0	Copper transport protein ATOX1	copper ion transport	8
P02049	Haemoglobin subunit beta	oxygen transport	8
Q0P569	Nucleobindin-1	signal transduction	6
Q3T0Y8	Vesicle-associated membrane protein 8	autophagosome maturation, defence response to virus, mucus secretion	7,10
Q5E983	Elongation factor 1-beta	translational elongation	7
Q9EQX9	Ubiquitin-conjugating enzyme E2 N	histone ubiquitination, DNA repair regulation, regulation of intracellular signal transduction, transcription regulation, protein ubiquitination	6,7,11
Q91XV3	Brain acid soluble protein 1	as above	2,7
P62077	Mitochondrial import inner membrane translocase subunit Tim8 B	protein transport	8
Q0PGG4	Actin, cytoplasmic 1	cell motility and contraction, transcription regulation, DNA damage response, DNA repair	3,7,9,11
B3EWE1	Haemoglobin subunit alpha	oxygen transport	8
B0VYY2	Cytochrome c oxidase subunit 5A, mitochondrial	mitochondrial electron transport	8
P11751	Haemoglobin subunit alpha	oxygen transport	8
Q8SPJ1	Junction plakoglobin	as above	3,4,6,7

Q8WN94	Acyl-CoA-binding protein	regulation of protein lipidation and phospholipid transport	7,8
Q9JLV1	BAG family molecular chaperone regulator 3	as above	2,5-9
A4FUI1	Coiled-coil domain-containing protein 58	unknown/not clear	14
Q9ESM2	Hyaluronan and proteoglycan link protein 2	cell adhesion, establishment of blood-nerve barrier, extracellular matrix assembly, glial cell differentiation, positive regulation of neuroblast proliferation	1,2,4
Q1LZ95	Isopentenyl-diphosphate Delta-isomerase 1	cholesterol biosynthesis	7
Q28895	Epididymal secretory protein E1	cholesterol transport and metabolism	7,8
Q3T087	60S ribosomal protein L11	translation, protein catabolism, signal transduction	6,7
P21571	ATP synthase-coupling factor 6, mitochondrial	as above	7,8
Q5M827	Pirin	monocyte and myeloid cell differentiation	2
B5DF11	AN1-type zinc finger protein 5	face development, fibroblast migration, platelet-derived growth factor receptor signalling pathway, respiratory system process, skeletal system morphogenesis, smooth muscle tissue development, vasculature development	2,3,6,7
A8WCF8	Tumor protein p63-regulated gene 1-like protein	calmodulin dependent kinase signalling pathway, negative regulation of synaptic transmission	6
Q5EB81	NADH-cytochrome b5 reductase 1	sterol biosynthesis	7
Q5PPH4	Zinc finger protein 414	transcription	7
Q64176	Carboxylesterase 1E	lipid metabolism, cholesterol transport, epithelial cell differentiation	2,7,8
P37089	Amiloride-sensitive sodium channel subunit alpha	ion transport, (taste) sensory transduction, regulation of blood pressure, cell response to acidic pH	7,8,13
A5D7J5	Rho-related GTP-binding protein RhoU	actin cytoskeleton organization, signal transduction, endocytosis, cell shape regulation, establishment or maintenance of cell polarity	4,6,7
O77559	ADM	signalling pathway, positive regulation of heart rate, regulation of systemic arterial blood pressure	6,7
O54939	Testosterone 17-beta-dehydrogenase 3	lipid metabolism	7
Q9D9V7	Protein DENND6B	unknown/not clear	14
**P00592**	**Phospholipase A2, major isoenzyme**	**fatty acid biosynthesis, lipid catabolism, intracellular signal transduction, neutrophil mediated immunity, regulation of immune response and IL-8 production, regulation of NAP kinase activity, regulation of transcription and podocyte apoptosis, regulation of cell population proliferation, regulation of calcium ion transport into cytosol**	**1,5-8,10**
Q8BGQ6	EF-hand calcium-binding domain-containing protein 14	metal ion binding	7
P09809	Apolipoprotein A-I	lipid transport and metabolism, regulation of phagocytosis, protein oxidation and stabilisation	7,8,10
Q00PI9	Heterogeneous nuclear ribonucleoprotein U-like protein 2	unknown/not clear	14

P58875	SEC14-like protein 2	transcription regulation, transport	7,8
P35433	Amidophosphoribosyltransferase	purine biosynthesis, cell cycle, cell response to xenobiotic stimulus, glutamine catabolism, lactation, kidney development	1,2,7,9
Q62924	A-kinase anchor protein 11	as above	4,7,8
**Q9Z0F8**	**Disintegrin and metalloproteinase domain-containing protein 17**	**apoptosis regulation, cell adhesion and motility, B cell differentiation, protein catabolism, inflammatory response, defence response to Gram positive bacterium, cell response to hypoxia, LPS, xenobiotic stimulus, regulation of cell growth and migration, and cell population proliferation, regulation of cold-induced thermogenesis, epidermal growth factor receptor signalling pathway**	**1-7,9,10**
Q9QYI6	DnaJ homolog subfamily B member 9	B cell differentiation, regulation of IL production, response to ER stress and unfolded protein	2,9,10
Q920B9	FACT complex subunit SPT16	nucleosome assembly, transcription regulation, DNA repair and replication	1,7,11
Q148N0	2-oxoglutarate dehydrogenase, mitochondrial	glycolysis, metabolism	7
Q6P5D4	Centrosomal protein of 135 kDa	centriole replication, centriole-centriole cohesion, regulation of protein localization establishment	1,4
Q9JL60	Glucocorticoid modulatory element-binding protein 1	transcription regulation	7
Q95MM9	Signalling lymphocytic activation molecule	adaptive and innate immune response, inflammatory response, natural killer cell proliferation and differentiation, signalling pathway, regulation of IL-6 and IL-12 production, regulation of catalytic activity, regulation of vesicle fusion	2,6,7,10
P52552	Peroxiredoxin-2 (Fragment)	cell redox homeostasis, response to oxidative stress, removal of superoxide radicals	7,9
P46892	Cyclin-dependent kinase 11B	cell cycle, cell growth regulation, regulation of apoptotic signalling pathway, mRNA processing, protein phosphorylation	1,4-7
Q9CXF4	TBC1 domain family member 15	activation and regulation of GTPase activity	7
Q9EPQ8	Transcription factor 20	transcription regulation	7
Q91WR3	Activating signal cointegrator 1 complex subunit 2	DNA repair, transcription regulation	7,11
Q9ERA5	Structural maintenance of chromosomes protein 4 (Fragment)	cell division	1
Q148E1	Apoptogenic protein 1, mitochondrial	intrinsic apoptotic signalling pathway, protein stabilisation, response to reactive oxygen species, regulation of reactive oxygen species biosynthesis	5-7,9
Q0VCX2	78 kDa glucose-regulated protein	cell response to glucose starvation and IL-4, apoptosis regulation, protein refolding, maintenance of protein localisation in ER, signalling pathway, cell migration regulation, **toxin transport**, cerebellum structural organisation	3-9
P54279	Mismatch repair endonuclease PMS2	DNA damage, DNA repair, mitotic cell cycle	1,9,11
Q9DB41	Mitochondrial glutamate carrier 2	transmembrane transport	8
Q9JLF7	Toll-like receptor 5	innate immune response, inflammatory response, regulation of IL-8 production, toll-like receptor signalling pathway, regulation of nitric oxide biosynthesis	6,7,10

P12263	Coagulation factor VIII	acute-phase response, blood coagulation	10
Q6DFX2	Anthrax toxin receptor 2	**toxin transport**, reproductive process	7,8
O08550	Histone-lysine N-methyltransferase 2B	transcription	7
Q99N92	39S ribosomal protein L27, mitochondrial	translation	7
Q8BJS8	Mdm2-binding protein	cell cycle, cell population proliferation, protein ubiquitination	1,7
Q9MZ03	ADP-ribosyl cyclase/cyclic ADP-ribose hydrolase 1	intracellular signal transduction	6
Q28730	Intercellular adhesion molecule 5	cell-cell adhesion	4
Q8CG48	Structural maintenance of chromosomes protein 2	cell division, cell cycle	1
Q3UMY5	Echinoderm microtubule-associated protein-like 4	cell cycle, cell division, microtubule cytoskeleton organisation	1,4
Q5E9I1	Cyclin-G1	cell cycle, cell division	1
Q8BRB7	Histone acetyltransferase KAT6B	transcription	7
Q2KIE4	Malignant T-cell-amplified sequence 1	DNA damage, cell cycle, cell growth regulation, protein biosynthesis	1,4,7,9
P34943	NADH dehydrogenase [ubiquinone] 1 alpha subcomplex subunit 9, mitochondrial	circadian rhythym, electron transport, respiratory chain	7,8
A5PJU9	Septin-1	cytokinesis, cell cycle, cell division, exocytosis regulation	1,7
		**fraction 40**	
Q6IMF3	Keratin, type II cytoskeletal 1	as above	4,10
Q9Z2T6	Keratin, type II cuticular Hb5	keratinisation	4
A1L595	Keratin, type I cytoskeletal 17	as above	2,4
P49065	Serum albumin	as above	4,5,9
O89106	Bis(5'-adenosyl)-triphosphatase	as above	1,5,7
Q6P7Q4	Lactoylglutathione lyase	as above	2,5,7
Q3YIX4	Phosphatidylethanolamine-binding protein 1	as above	5-7,9,12
Q9N0F1	Dihydrolipoyllysine-residue succinyltransferase component of 2-oxoglutarate dehydrogenase complex, mitochondrial	as above	7
Q3T140	Dynein light chain roadblock-type 1	as above	8
P18203	Peptidyl-prolyl cis-trans isomerase FKBP1A	as above	1,2,6,9,10

B0VYY2	Cytochrome c oxidase subunit 5A, mitochondrial	as above	8
Q9CWM4	Prefoldin subunit 1	actin cytoskeleton organisation, B cell activation, protein folding, cerebellum development	2,4,7,10
P54149	Mitochondrial peptide methionine sulfoxide reductase	cell response to oxidative stress	9
Q9JLV1	BAG family molecular chaperone regulator	autophagosome assembly, brain development, transcription regulation, cell response to heat, mechanical stimulus, apoptosis, regulation of protein transport to nucleus, protein stabilisation, muscle cell cellular homeostasis	2,5,7-9
Q3ZBZ8	Stress-induced-phosphoprotein 1	protein folding	7
H6BDU4	Superoxide dismutase [Cu-Zn]	as above	9
Q9CXP8	Guanine nucleotide-binding protein G(I)/G(S)/G(O) subunit gamma-10	signalling pathway	6
P02102	Haemoglobin subunit epsilon-1	oxygen transport	8
Q66HD3	Nuclear autoantigenic sperm protein	blastocyst development, DNA replication, mitotic cell cycle, protein transport	1,2,8
Q3T087	60S ribosomal protein L11	as above	6,7
Q921H9	Cytochrome c oxidase assembly factor 7	as above	7
Q3T0E0	Copper transport protein ATOX1	as above	8
P62959	Histidine triad nucleotide-binding protein 1	transcription regulation, catabolism, apoptosis, signalling pathway	5-7
Q8WN94	Acyl-CoA-binding protein	as above	7,8
A4FUI1	Coiled-coil domain-containing protein 58	unknown/not clear	14
Q5NRP9	Endothelin-1	endothelin receptor signalling pathway, regulation of vasoconstriction	6,7
Q9GKK4	Galactokinase	galactose metabolism	7
Q6PEC1	Tubulin-specific chaperone A	protein folding	7
Q2TBK8	Snurportin-1	as above	7,8
Q28895	Epididymal secretory protein E1	as above	7,8
Q2NKU6	Protein dpy-30 homolog	endosomal transport, transcription regulation	7,8
Q1LZ95	Isopentenyl-diphosphate Delta-isomerase 1	cholesterol biosynthesis	7
Q0VCG3	Parvalbumin alpha	inhibitory chemical synaptic transmission	6
Q91ZF1	ATP-sensitive inward rectifier potassium channel 15	ion transport	8
Q1LZH0	U11/U12 small nuclear ribonucleoprotein 35 kDa protein	mRNA splicing	7
Q8WMS0	UDP-galactose translocator	carbohydrate transport	8
P11751	Haemoglobin subunit alpha	oxygen transport	8

Q2KIR1	U1 small nuclear ribonucleoprotein A	mRNA splicing	7
Q3ZCH0	Stress-70 protein, mitochondrial	erythrocyte differentiation, protein export from nucleus, protein refolding	2,7,8
P00515	cAMP-dependent protein kinase type II-alpha regulatory subunit	negative regulation of cAMP-dependent protein kinase activity	7
Q91ZT7	Ankyrin repeat and SOCS box protein 10	intracellular signal transduction, protein ubiquitination	6,7
Q9JI38	tRNA pseudouridine(38/39) synthase	biosynthesis	7
Q6P9Z6	Tumor-associated calcium signal transducer 2	regulation of cell motility, regulation of epithelial cell proliferation, morphogenesis	1-3
P31783	T-cell surface glycoprotein CD8 alpha chain	cell surface receptor signalling pathway, cytotoxic T cell differentiation, T cell mediated immunity	2,6,10
Q3MKQ1	Protein BEX2	apoptosis, cell cycle, signal transduction, transcription regulation	1,6,7
A2RUW1	Toll-interacting protein	as above	2,6-8,10
Q5FVR0	T-cell immunoglobulin and mucin domain-containing protein 2	as above	7,8,10
Q6DFX2	Anthrax toxin receptor 2	as above	7,8
Q8BH34	Semaphorin-3D	negative chemotaxis, nervous system development, regulation of cell migration, signalling pathway	2,3,6
Q7YRA3	Exosome complex component RRP41	defence response to virus, rRNA processing, cell growth regulation	4,7,10
Q2T9U2	Outer dense fiber protein 2	cell differentiation	2
Q91XV3	Brain acid soluble protein 1	as above	2,7
Q3SYZ4	Aspartate--tRNA ligase, cytoplasmic	protein biosynthesis	7
P50232	Synaptotagmin-4	brain development, memory, cell differentiation, exocytosis and endocytosis, regulation of protein secretion, negative regulation of vesicle fusion, neurotransmitter secretion, signal transduction	2,6,7
Q3SZ62	Phosphoglycerate mutase 1	glycolysis	7
Q8R151	NFX1-type zinc finger-containing protein 1	DNA replication, innate immune response, defence response to bacterium and virus	1,10
Q3V3V9	Capping protein, Arp2/3 and myosin-I linker protein 2	regulation of actin cytoskeleton organisation, cell migration, establishment of protein localisation, maintenance of cell polarity, regulation of IL-2 production, regulation of T-cell differentiation and proliferation, T cell receptor signalling pathway, thymus development	1-4,6,8,10
P97679	DNA mismatch repair protein Mlh1	DNA damage, DNA repair, cell cycle, cell division, immune response, response to bacterium, hypoxia, xenobiotic stimulus and **toxic substances**	1,9-11
Q8HYY4	Uveal autoantigen with coiled-coil domains and ankyrin repeats protein	apoptosis, regulation of cell growth and motility	3-5

P43023	Cytochrome c oxidase subunit 6A2, mitochondrial	mitochondrial electron transport	8
Q5EAD4	Short/branched chain specific acyl-CoA dehydrogenase, mitochondrial	lipid metabolism	7
P54279	Mismatch repair endonuclease PMS2	DNA damage, DNA repair, cell cycle, immune response	1,7,9,11
P00586	Thiosulfate sulfurtransferase	rRNA transport	8
A6QM06	Sterol regulatory element-binding protein cleavage-activating protein	cholesterol metabolism	7
**P14422**	**Phospholipase A2**	**secretion, lipid catabolism, inflammatory response, defence response to Gram positive bacterium**	**7,10**
O08550	Histone-lysine N-methyltransferase 2B	as above	7
Q9D361	U11/U12 small nuclear ribonucleoprotein 48 kDa protein	mRNA processing, RNA splicing	7
Q0VCR8	Exocyst complex component 3-like protein	exocytosis, peptide hormone secretion	7
Q9D799	Methionyl-tRNA formyltransferase, mitochondrial	translation	7
Q9DB41	Mitochondrial glutamate carrier 2	as above	8
Q9JLF7	Toll-like receptor 5	as above	6,7,10
P02680	Fibrinogen gamma chain	regulation of exocytosis and peptide hormone secretion, protein polymerization, protein secretion, regulation of cell-cell adhesion, blood coagulation, cell-matrix adhesion, cell response to IL-1 and IL-6, plasminogen activation, regulation of platelet aggregation, apoptosis, response to calcium ion	4,5,7,10
Q8BJS8	Mdm2-binding protein	as above	1,7
P35479	Leukocyte cysteine proteinase inhibitor 1	regulation of peptidase activity	7
A4K436	Regulator of telomere elongation helicase 1	DNA repair and replication	1,11
Q924Y8	Probable G-protein coupled receptor 149	neuropeptide signalling pathway	6
Q8VI38	Globoside alpha-1,3-N-acetylgalactosaminyltransferase 1	carbohydrate metabolism, lipid and protein glycosylation	7
Q8BQ48	Centrosomal protein of 295 kDa	cell cycle, cell division	1
P46892	Cyclin-dependent kinase 11B	cell cycle, apoptosis, cell growth regulation, protein phosphorylation, mRNA processing	1,4,5,7
P24643	Calnexin	endocytosis	7
Q27975	Heat shock 70 kDa protein 1A	stress response, lysosomal transport, protein refolding, transcription regulation	7-9
Q9XTA2	Prolyl endopeptidase	proteolysis	7

Q9JJA2	Conserved oligomeric Golgi complex subunit 8	glycosylation, protein transport, Golgi organisation	4,7,8
Q99P69	Kinetochore protein Nuf2	cell cycle, cell division	1
Q3MHM6	Catenin alpha-1	cell migration and adhesion, regulation of protein localisation to nucleus	3,4,8
Q8SPJ1	Junction plakoglobin	as above	3,4,6,7
P11708	Malate dehydrogenase, cytoplasmic	metabolism, oxidoreductase	7
Q8K0Z7	Translational activator of cytochrome c oxidase 1	translation regulation	7
O02810	Phosphatidylinositol 4-kinase beta	lipid metabolism	7
D3ZZL9	GRIP and coiled-coil domain-containing protein 2	protein transport, Golgi formation, microtubule anchoring	4,8
P61603	10 kDa heat shock protein, mitochondrial	stress response	9
A1XQU3	60S ribosomal protein L14	translation, ribosomal biogenesis	7
P83095	Serine beta-lactamase-like protein LACTB, mitochondrial	lipid metabolism, proteolysis	7

[a]hyaluronidase is devoid of toxic activity but acts as a toxin spreading factor commonly found in animal venoms
