# Supplementary material for: Proteins from shrews’ venom glands play a role in gland functioning and venom production
Source: Zoological Lett. 2024 Jul 15;10:12. doi: 10.1186/s40851-024-00236-x (PMC11251227; doi:10.1186/s40851-024-00236-x)
Supplement: Supplementary file 4 — Additional file 4: table A4: Protein identification in the extract from venom glands of the common shrew Sorex araneus based on tandem mass spectrometry analysis. Toxins are shown in bold. Peptide sequences unique to a specific protein are marked in red [file 40851_2024_236_MOESM4_ESM.pdf]

Table A4 Protein identification in the extract from venom glands of the common shrew *Sorex araneus* based on tandem mass spectrometry analysis. Toxins are shown in bold. Peptide sequences unique to a specific protein are marked in red.

Accession code	Matched peptides	Protein sequence coverage [%]	Ion score	m/z	emPAI	Identified peptides	Protein name	Protein label	Species
						**whole extract**			
P51434	45	16	46	1097	5.96	K.KFFQMVGLK.K	Parvalbumin alpha (Fragment)	pvalb	*Cavia porcellus*
			37	1113		K.KFFQMVGLK.K+ Oxidation (M)			
			59	1113		K.FFQMVGLK.K			
			62	984		K.FFQMVGLK.K+ Oxidation (M)			
			51	722		K.GFSADAR.D			
P63312	7	34	56	862	8.03	K.KTETQEK.N	Thymosin beta-10	tmsb10	*Rattus norvegicus*
			37	734		K.TETQEK.N			
			48	875		K.ETIEQEK.R			
			77	1031		K.ETIEQEKR.S			
P07107	76	39	53	1987	7.06	K.TKPADEEMLFIYSHYK.Q + Oxidation (M)	Acyl-CoA-binding protein	dbi	*Bos taurus*
			123	1991		K.QATVGDINTERPGMLDFK.G			
			73	2007		K.QATVGDINTERPGMLDFK.G + Oxidation (M)			
P18203	23	25	75	1314	1.74	M.GVQVETISPGDGR.T	Peptidyl-prolyl cis-trans isomerase FKBP1A	fkbp1a	*Bos taurus*
			113	1533		R.GWEEGVAQMSVGQR.A			
			118	1549		R.GWEEGVAQMSVGQR.A + Oxidation (M)			
Q2KJ32	33	19	104	2413	0.72	K.GTWEQPGGAAPMGYDFWYQPR.H	Selenium-binding protein 1	selenbp1	*Bos taurus*
			82	1837		R.HNVMISTEWAAPNVLR.D			
			78	1104		K.NQGGTWSVEK.V			
			78	1411		K.GGPVQVLEDQELK.C			
			43	1050		K.QFYPDLIR.E			
			84	1263		K.LNPNFLVDFGK.E			
			60	2546		K.LNPNFLVDFGKEPLGPALAHELR.Y			
			70	1302		K.EPLGPALAHELR.Y			
Q2KJG2	27	68	53	901	5.86	K.ITLTSDPR.L	Ubiquitin-fold modifier 1	ufm1	*Bos taurus*
			100	1587		K.VLSVPESTPFTAVLK.F			

			66	840		K.FAAEEFK.V			
			80	2752		K.VPAATSAIITNDGIGINPAQTAGNVFLK.H			
P04906	20	13	93	1351	0.49	M.PPYTIVYFPVR.G	Glutathione S-transferase P	gstp1	*Rattus norvegicus*
			82	1276		R.MLLADQGQSWK.E			
			65	736		R.SLGLYGK.D			
Q9DBJ1	22	40	76	1311	1.98	R.HGESAWNLENR.F	Phosphoglycerate mutase 1	pgam1	*Mus musculus*
			61	1058		R.HYGGLTGLNK.A			
			102	1683		R.ALPFWNEEIVPQIK.E			
			61	1150		R.VLIAAHGNSLR.G			
			34	3038		K.HLEGLSEEAIMELNLPTGIPIVYELDK.N + Oxidation (M)			
			36	2114		K.NLKPIKPMQFLGDEETVR.K			
			54	2130		K.NLKPIKPMQFLGDEETVR.K + Oxidation (M)			
			118	1102		R.KAMEAVAAQGK.V			
			56	1118		R.KAMEAVAAQGK.V + Oxidation (M)			
B3EWE1	27	43	67	998	2.41	R.TFASFPTTK.T	Haemoglobin subunit alpha	hba	*Blarina brevicauda*
			88	1793		K.TYFPHFDLSPGSAQVK.G			
			75	844		K.KVADALTK.A			
			64	2186		K.AVGSLDDLPGALSALSDLHAHK.L			
			60	817		R.VDPVNFK.L			
P21571	25	21	62	827	1.08	K.ELDPVQK.L	ATP synthase-coupling factor 6, mitochondrial	atp5j	*Rattus norvegicus*
			54	882		K.LKQMYGK.G + Oxidation (M)			
			91	1061		K.FEVLDKPQS.-			
Q5E956	12	16	89	1602	0.60	K.VVLAYEPVWAIGTGK.T	Triosephosphate isomerase	tpi1	*Bos taurus*
			100	1466		K.TATPQQAQEVHEK.L			
			74	1203		K.SNVSDAVAQSAR.I			
Q3MHL6	18	18	60	975	2.91	K.SHLMYAVR.E	TSC22 domain family protein 1	tsc22d1	*Bos taurus*
			35	991		K.SHLMYAVR.E + Oxidation (M)			
			70	844		R.EEVEVLK.E			
			92	1429		K.NSQLEQENNLLK.T			
Q3SZR3	36	11	52	1145	0.49	K.WFYIGSAFR.N	Alpha-1-acid glycoprotein	orm1	*Bos taurus*
			64	1686		K.DACGPLEKQHEEER.K			
Q3T140	24	16	53	1636	0.31	K.GVQGIIVVNTEGIPIK.S	Dynein light chain roadblock-type 1	dynlrb1	*Bos taurus*

Q2KIV2	12	34	52	970	2.11	K.EFLGTYNK.L	Mitochondrial import inner membrane translocase subunit Tim9	timm9	*Bos taurus*
			130	1789		R.FQEYHIQQNEALAAK.A			
			77	810		K.AGLLGQPR.-			
Q5FZP5	9	7	97	2952	0.20	R.TNEIVEEQYTPQSLATLESVFQELGK.L	Secretogranin-2	scg2	*Sus scrofa*
			94	1267		R.QYLDEDLLMK.V			
			65	1134		K.VLEYLNQEK.A			
Q2EN75	16	15	62	730	1.42	K.ELTIGAK.L	Protein S100-A6	s100a6	*Sus scrofa*
			58	876		K.LMDDLDR.N			
			58	892		K.LMDDLDR.N + Oxidation (M)			
Q9D0S9	6	12	125	2219	0.20	R.ISQAEEDDQQLLGHLLLVAK.K	Histidine triad nucleotide-binding protein 2, mitochondrial	hint2	*Mus musculus*
Q8R317	6	2	113	1909	0.07	R.FQQQLEQLSAMGFLNR.E	Ubiquilin-1	ubqln1	*Mus musculus*
Q2HJ98	14	16	30	851	1.33	R.FWEWGK.N	Acylpyruvase FAHD1, mitochondrial	fahd1	*Bos taurus*
			50	873		R.NYADHVR.E			
			34	1013		K.KGLPWTLAK.S			
			59	884		K.GLPWTLAK.S			
			74	1486		K.SFTASCPVSAFVPK.E			
Q3ZC22	7	14	86	1295	0.41	K.FQTMSDQIIGR.I	Heat shock factor-binding protein 1	hsbp1	*Bos taurus*
P47727	10	7	61	1582	0.31	R.FHQLDIDNPQSIR.A	Carbonyl reductase [NADPH] 1	cbr1	*Rattus norvegicus*
			69	843		K.IGVTVLSR.I			
Q8WNN6	10	18	113	1167	0.77	R.HVGDLGNVTAGK.D	Superoxide dismutase [Cu-Zn]	sod1	*Canis lupus familiaris*
			53	842		R.TMVVHEK.R			
			58	989		R.LACGVIGIAQ.-			
P58044	13	14	44	769	0.61	K.LLLQQR.S	Isopentenyl-diphosphate Delta-isomerase 1	idi1	*Mus musculus*
			61	1353		K.NVTLNPDPNEIK.S			
			34	790		K.LTPWFK.I			
			46	1067		K.IIADTFLFK.W			
Q3ZBS8	7	22	65	1353	0.71	R.FIDTSQFILNR.L	Mitochondrial import inner membrane translocase subunit Tim8 A	timm8a	*Bos taurus*
			74	1194		K.SKPVFSESLSD.-			
Q5S3G4	4	8	82	1200	0.24	K.GLDPYNILAPK.A	Cytochrome c oxidase subunit 5B, mitochondrial	cox5b	*Sus scrofa*
Q8K2C6	6	3	78	1001	0.13	R.SPICPALAGK.G	NAD-dependent protein deacylase sirtuin-5, mitochondrial	sirt5	*Mus musculus*

P01283	4	11	69	1353	0.55	R.HADGVFTSDYSR.L	VIP peptides	vip	*Rattus norvegicus*
			77	828		R.LLGQISAK.K			
Q1ZZU7	4	9	76	1273	0.62	M.PMFVVNTNVPR.A	Macrophage migration inhibitory factor	mif	*Ovis aries*
			66	1289		M.PMFVVNTNVPR.A + Oxidation (M)			
Q2NKU6	3	16	74	1886	0.31	K.ERPPNPIEFLASYLLK.N	Protein dpy-30 homolog	dpy30	*Bos taurus*
P00435	5	9	45	1137	0.44	R.NDVSWNFEK.F	Glutathione peroxidase 1	gpx1	*Bos taurus*
			68	1155		K.FLVGPDGVPVR.R			
Q5E983	2	6	100	1603	0.18	K.SPAGLQVLNDYLADK.S	Elongation factor 1-beta	eef1b	*Bos taurus*
P00921	2	3	78	972	0.15	K.VLDALDSIK.T	Carbonic anhydrase 2	ca2	*Bos taurus*
Q6P7Q4	3	20	51	1264	0.56	K.DFLLQQTMLR.I	Lactoylglutathione lyase	glo1	*Rattus norvegicus*
			42	976		K.RFEELGVK.F			
			74	2288		K.GLAFVQDPDGYWIEILNPNK.M			
Q0IIJ2	9	4	39	986	0.22	K.RLVTTGVLK.Q	Histone H1.0	h1f0	*Bos taurus*
Q9BDP9	2	16	75	1284	0.69	K.ELIEALQEVLK.K	Cocaine- and amphetamine-regulated transcript protein (Fragment)	cartpt	*Sus scrofa*
A2RUW1	2	5	76	1603	0.15	R.GPVYIGELPQDFLR.I	Toll-interacting protein	tollip	*Rattus norvegicus*
Q4LAL9	3	2	57	1172	0.10	K.NIFSFYLNR.D	Cathepsin D	ctsd	*Canis lupus familiaris*
Q04447	3	5	63	1302	0.22	K.VLTPELYAELR.A	Creatine kinase B-type	ckb	*Mus musculus*
			28	1253		R.HGGYQPSDEHK.T			
Q3YIX4	2	4	69	884	0.22	K.VLTPTQVK.N	Phosphatidylethanolamine-binding protein 1	pebp1	*Canis lupus familiaris*
Q9QZM0	1	2	97	1936	0.06	R.FQQQLEQLNAMGFLNR.E	Ubiquilin-2	ubqln2	*Mus musculus*
Q3SZJ9	4	2	48	868	0.16	R.GTFIEFR.N	Phosphomannomutase 2	pmm2	*Bos taurus*
Q0PGG4	2	4	69	975	0.22	K.AGFAGDDAPR.A	Actin, cytoplasmic 1	actb	*Bos mutus grunniensis*
			52	794		K.IIAPPER.K			
Q9N0F1	1	3	91	1478	0.09	K.TPAFAESVTEGDVR.W	Dihydrolipoyllysine-residue succinyltransferase component of 2-oxoglutarate dehydrogenase complex, mitochondrial	dlst	*Sus scrofa*
P62958	5	5	35	844	0.35	R.QMNWPPG.-+ Oxidation (M)	Histidine triad nucleotide-binding protein 1	hint1	*Bos taurus*

Q8BH83	3	3	49	1496		R.DQLPVWLLEDIR.A	Ankyrin repeat domain-containing protein 9	ankrd9	*Mus musculus*
Q56K04	2	11	54	932	0.96	R.GGAESHTFK.-	Cysteine-rich protein 1	crip1	*Bos taurus*
Q3SZ18	2	10	33	1178	0.29	K.SIPMTVDFIR.L	Hypoxanthine-guanine phosphoribosyltransferase	hprt1	*Bos taurus*
			67	1277		K.VIGGDDLSTLTGK.N			
Q6B4U9	2	10	43	979	0.45	K.IGHPAPNFK.A	Peroxiredoxin-1	prdx1	*Myotis lucifugus*
			53	1211		R.QITVNDLPVGR.S			
Q3ZBD3	2	9	48	1129	0.41	R.AVGWNELEGR.D	Pterin-4-alpha-carbinolamine dehydratase	pcbd1	*Bos taurus*
Q9D6Y7	2	9	48	867	0.27	K.TGHAEVVR.V	Mitochondrial peptide methionine sulfoxide reductase	msra	*Mus musculus*
			36	1632		K.VFWENHDPTQGMR.Q + Oxidation (M)			
P04444	3	7	30	1114	0.28	K.KVLTSLGLGVK.N	Haemoglobin subunit beta-H1	hbb-bh1	*Mus musculus*
Q9JLS0	2	12	53	877	0.78	K.GLPDHPSR.G	Hypoxia-inducible lipid droplet-associated protein	hilpda	*Mus musculus*
Q8BHX3	2	2	51	858	0.14	K.QALEEAAK.A	Borealin	cdca8	*Mus musculus*
Q3UDR8	5	2	35	1021	0.12	R.DIPAVLPAAR.L	Protein YIPF3	yipf3	*Mus musculus*
Q2YDH6	2	4	46	900	0.21	K.VPNLPSFK.-	AP-3 complex subunit sigma-1	ap3s1	*Bos taurus*
Q3C2I0	2	3	38	1124	0.23	R.TIFNQVMEK.E+ Oxidation (M)	Bcl-2-related protein A1	bcl2a1	*Bos taurus*
Q8BIQ3	10	2	38	928	0.09	-.MAAVSPPTR.C	Zinc finger protein 2	znf2	*Mus musculus*
Q0Z8U2	1	6	50	1573	0.17	K.GGKPEPPAMPQPVPTA.-	40S ribosomal protein S3	rps3	*Sus scrofa*
B1ARW8	1	10	46	1270	0.44	R.AQQLLDAVEQR.Q	Uncharacterized protein C1orf122 homolog	c1orf122	*Mus musculus*
Q99LJ8	6	2	29	782	0.13	R.KPRAVGR.N	Dehydrodolichyl diphosphate synthase complex subunit Nus1	nus1	*Mus musculus*
O77512	3	3	32	996	0.13	K.LLPSLPETK.N	Glycine N-phenylacetyltransferase	-	*Bos taurus*
Q5HZE0	2	2	35	829	0.14	R.GLASTLLR.A	Mitochondrial basic amino acids transporter	slc25a29	*Rattus norvegicus*
Q9D483	1	3	36	1429	0.07	R.GPPPPAPTLVINEK.D	DNA-directed RNA polymerase III subunit RPC3	polr3c	*Mus musculus*
Q3UHX2	1	3	34	931	0.22	R.EREEIEK.Q	28 kDa heat- and acid-stable phosphoprotein	pdap1	*Mus musculus*

Q5GAL7	2	5	32	1040	0.26	R.LYLLCSRK.H	Probable inactive ribonuclease-like protein 13	rnase13	*Rattus norvegicus*
O35507	1	2	34	1289	0.08	K.TLMAKVVSDGIR.D	Peroxisome proliferator-activated receptor alpha	ppara	*Cavia porcellus*
A2AIW0	1	2	34	1031	0.09	R.SGQGASLSVVK.Q	Serologically defined colon cancer antigen 3 homolog	sdccag3	*Mus musculus*
Q9R0M6	2	4	31	983	0.20	-.MAGKSSLFK.I+ Oxidation (M)	Ras-related protein Rab-9A	rab9a	*Mus musculus*
Q9D8Z2	1	17	32	1356	0.39	K.GDGSGDPCTDLFK.R	TP53-regulated inhibitor of apoptosis 1	triap1	*Mus musculus*
Q5BIP7	1	2	32	1326	0.10	R.VFGRYVFSPVR.E	Lipoyl synthase, mitochondrial	lias	*Bos taurus*
Q3SZI5	3	2	31	800	0.13	K.GTSPNVAR.N	Mitochondrial uncoupling protein 2	ucp2	*Bos taurus*
Q8BYH0	1	3	31	1062	0.16	R.TASVAALRMK.A+ Oxidation (M)	Dorsal root ganglia homeobox protein	drgx	*Mus musculus*
Q80W32	1	2	31	1005	0.09	K.STSLLTKEK.S	IQ domain-containing protein G	iqcg	*Mus musculus*
Q8BGQ6	2	2	30	1201	0.08	K.LNEELLSKQK.Q	EF-hand calcium-binding domain-containing protein 14	efcab14	*Mus musculus*
Q9CZ69	1	3	29	799	0.23	R.KPENNAK.V	CKLF-like MARVEL transmembrane domain-containing protein 6	cmtm6	*Mus musculus*
Q7TMI3	1	2	28	1873	0.05	K.QNDAQVKPSSHNPPKVK.K	E3 ubiquitin-protein ligase UHRF2	uhrf2	*Mus musculus*
Q8BKV1	1	2	27	1785	0.07	R.GLVEDSGSFLIHTLAAR.H	Glypican-2	gpc2	*Mus musculus*
P26260	18	13	30	1086	0.10	EKEATTRPR	Syndecan-1	sdc1	*Rattus norvegicus*
Q08DK5	18	33	30	760	0.10	LDLDASK	Endophilin-B2	sh3glb2	*Bos taurus*
Q865B6	24	20	29	800	0.08	QVSPGSAR	Peroxisome proliferator-activated receptor gamma coactivator 1-alpha	ppargc1a	*Sus scrofa*
Q3SZE2	5	10	29	865	0.07	MFILQSK	Prefoldin subunit 1	pfdn1	*Bos taurus*
Q8VC56	17	10	28	1106	0.09	MVDNLSSDVK	E3 ubiquitin-protein ligase RNF8	rnf8	*Mus musculus*

Q0P565	13	35	28	780	0.08	TGWVYR	HD domain-containing protein 2	hddc2	*Bos taurus*
Q9ERA5	10	10	28	748	0.10	LDMINK + Oxidation (M)	Structural maintenance of chromosomes protein 4 (Fragment)	smc4	*Microtus arvalis*
Q11126	11	9	27	1138	0.07	MYPPGCAKVK	Galactoside 3(4)-L-fucosyltransferase	fut3	*Bos taurus*
Q5H8C4	15	25	27	847	0.17	KDMVDIK	Vacuolar protein sorting-associated protein 13A	vps13a	*Mus musculus*
P19803	28	9	27	1917	0.05	SIQEIQELDKDDESLR	Rho GDP-dissociation inhibitor 1	arhgdia	*Bos taurus*
O97594	21	11	27	1035	0.10	ELEKMTNR + Oxidation (M)	Structural maintenance of chromosomes protein 3	smc3	*Bos taurus*
Q5EAD3	15	16	27	773	0.17	ARGEADR	Transcription factor NF-E2 45 kDa subunit	nfe2	*Bos taurus*
P15690	11	14	26	883	0.06	MLRIPVR	NADH-ubiquinone oxidoreductase 75 kDa subunit, mitochondrial	ndufs1	*Bos taurus*
Q91YS8	30	21	26	1095	0.08	PGAVEGPRWK	Calcium/calmodulin-dependent protein kinase type 1	camk1	*Mus musculus*
Q8CE90	23	10	26	1735	0.11	HYEILEVDVASWFK	Dual specificity mitogen-activated protein kinase 7	map2k7	*Mus musculus*
P97313	10	11	25	733	0.13	VMLSLR + Oxidation (M)	DNA-dependent protein kinase catalytic subunit	prkdc	*Mus musculus*
P33705	21	8	25	1240	0.07	LSMKEAHAPLK + Oxidation (M)	T-cell surface glycoprotein CD4	cd4	*Canis lupus familiaris*
O47558	15	30	25	809	0.02	DVLGFLM + Oxidation (M)	Cytochrome b (Fragment)	mt-cyb	*Lepus alleni*
Q9D483	24	10	25	1429	0.01	GPPPPAPTLVINEK	DNA-directed RNA polymerase III subunit RPC3	polr3c	*Mus musculus*
P62157	23	10	25	1265	0.05	DGNGYISAAELR	Calmodulin	calm	*Bos taurus*
Q9BEG2	12	13	24	821	0.23	AKIDVCK	Interleukin-12 receptor subunit beta-2	il12rb2	*Bos taurus*
Q8BLK9	15	14	24	847	0.12	EASAMDPK	Ribosomal protein S6 kinase delta-1	rps6kc	*Mus musculus*
G3MWR8	18	12	24	931	0.23	EKDLDGAGK	Protein-methionine sulfoxide oxidase MICAL3	mical3	*Bos taurus*

P05126	11	8	24	1142	0.04	IYIQAHIER	Protein kinase C beta type	prkcb	*Bos taurus*
Q3MHE4	14	10	24	974	0.19	TLESVVLSK	DNA mismatch repair protein Msh2	msh2	*Bos taurus*
O35314	9	11	24	792	0.16	APHLDLK	Secretogranin-1	chgb	*Rattus norvegicus*
E9Q6J5	35	12	24	1651	0.22	GVGPLMAVGTRGEHDR	Biorientation of chromosomes in cell division protein 1-like 1	bod11	*Mus musculus*
P26954	14	11	23	911	0.18	EKIPNPSK	Interleukin-3 receptor class 2 subunit beta	csf2rb2	*Mus musculus*
A3KFM7	25	16	23	1015	0.38	AEILGEAADK	Chromodomain-helicase-DNA-binding protein 6	chd6	*Mus musculus*
Q330H0	30	14	23	1368	0.19	QYSPRSMEAATK	NADH-ubiquinone oxidoreductase chain 2	mt-nd2	*Rhynchonycteris naso*
Q9JLM2	29	9	23	1960	0.20	QSQSIAKESLTIHEYVK	Natural killer cell receptor 2B4	cd244	*Rattus norvegicus*
Q3SZK4	16	19	23	939	0.24	MVVAMMMK	Protein TBRG4	tbrg4	*Bos taurus*
P57784	26	10	23	1228	0.17	KGGPSAGDVEAIK	U2 small nuclear ribonucleoprotein A'	snrpa1	*Mus musculus*
Q8K202	14	38	23	703	0.34	MIEIAK	DNA-directed RNA polymerase I subunit RPA49	polr1e	*Mus musculus*
**Q10741**	**9**	**11**	**23**	**809**	**0.17**	**LYSDGKK**	**Disintegrin and metalloproteinase domain-containing protein 10**	**adam10**	***Bos taurus***
P23726	8	7	23	1020	0.34	YQQDQIVK	Phosphatidylinositol 3-kinase regulatory subunit beta	pik3r2	*Bos taurus*
Q6NZP1	29	17	23	1175	0.22	LQATEDDKEK	DNA annealing helicase and endonuclease ZRANB3	zranb3	*Mus musculus*
Q3SX42	15	12	23	916	0.38	KTVDDVIK	Charged multivesicular body protein 2b	chmp2b	*Bos taurus*
Q9N0J6	31	22	22	1030	0.21	EAIITAKER	Fructose-1,6-bisphosphatase isozyme 2	fbp2	*Oryctolagus cuniculu*
Q8BW10	20	12	22	1185	0.34	ELQELLIDGR	RNA-binding protein NOB1	nob1	*Mus musculus*

Q8VD75	24	15	22	1230	0.39	WLTQIAELEK	Huntingtin-interacting protein 1	hip1	*Mus musculus*
Q3SYS1	11	7	22	1020	0.28	LAIYGMLPK + Oxidation (M)	39S ribosomal protein L13, mitochondrial	mrpl13	*Bos taurus*
Q60560	19	16	22	884	0.27	DRALDPAK	DNA-binding protein SMUBP-2	ighmbp2	*Mesocricetus auratus*
P50310	16	23	22	831	0.65	DASGNKIK	Phosphoglycerate kinase 1	pgk1	*Cricetulus griseus*
Q58DC5	17	7	22	1122	0.33	QTATILSMDK + Oxidation (M)	GTP-binding protein 1	gtpbp1	*Bos taurus*
A5PK65	18	11	22	911	0.02	KGTVMTFL + Oxidation (M)	D-dopachrome decarboxylase	ddt	*Bos taurus*
Q8CFD4	17	22	22	960	0.20	EENDVVEK	Sorting nexin-8	snx8	*Mus musculus*
P28800	29	12	22	1366	0.22	LDNQEPGGQIAPK	Alpha-2-antiplasmin	serpinf2	*Bos taurus*
Q91Y47	10	11	21	846	0.03	MICAGYK + Oxidation (M)	Coagulation factor XI	f11	*Mus musculus*
Q63880	14	18	21	902	0.59	EGKDLITK	Carboxylesterase 3A	ces3a	*Mus musculus*
Q9Z2E1	22	10	21	1497	0.20	QPVTKFTNHPSNK	Methyl-CpG-binding domain protein 2	mbd2	*Mus musculus*
O55036	23	14	21	1086	0.73	ATESRIPVSK	Telomeric repeat-binding factor 1 (Fragment)	terf1	*Cricetulus griseus*
P19973	15	14	21	841	0.31	AEAAIDPR	Lymphocyte-specific protein 1	lsp1	*Mus musculus*
Q80WC3	16	10	21	1222	0.16	SCIIDKEELK	Trinucleotide repeat-containing gene 18 protein	tnrc18	*Mus musculus*
						**fraction 23**			
Q6IMF3	46	10	79	1384	0.79	K.SLNDKFASFIDK.V	Keratin, type II cytoskeletal 1	krt1	*Rattus norvegicus*
			71	826		K.FASFIDK.V			
			97	1475		R.FLEQQNQVLQTK.W			
			84	1475		K.WELLQQVDTSTR.T			
			52	909		K.YEDEINK.R			
			71	1065		K.YEDEINKR.T			
			89	1265		R.TNAENEFVTIK.K			
			63	1140		R.DYQELMNTK.L			
			48	1156		R.DYQELMNTK.L+ Oxidation (M)			
Q5XLE4	4	2	98	1609	0.06	K.DVFLGTFLYEYSR.R	Serum albumin	alb	*Equus asinus*
P34032	69	88	46	1245	244.04	M.ADKPDMAEIEK.F	Thymosin beta-4	tmsb4	*Oryctolagus cuniculus*
			47	1652		M.ADKPDMAEIEKFDK.S + Oxidation (M)			
			34	862		K.KTETQEK.N			
			38	734		K.TETQEK.N			

			83	1371		K.TETQEKNPLPSK.E			
			89	1512		K.NPLPSKETIEQEK.Q			
			43	875		K.ETIEQEK.Q			
			72	1348		K.ETIEQEKQAGES.-			
B3EWE1	5	6	67	998	0.23	R.TFASFPTTK.T	Haemoglobin subunit alpha	hba	*Blarina brevicauda*
P18174	3	5	50	1003	0.28	K.QQESQEQK.L	Involucrin	ivl	*Canis lupus familiaris*
			31	902		K.EQLEQEK.K			
Q07DX1	3	7	38	1644	0.22	K.YVDSEGHLYTVPVR.E	Caveolin-1	cav1	*Muntiacus reevesi*
Q3T0F4	2	8	48	1441	0.18	K.AEAGAGSATEFQFR.G	40S ribosomal protein S10	rps10	*Bos taurus*
Q3V1I0	1	4	47	1055	0.19	R.ILIKEVGQR.H	Lysozyme g-like protein 2	lyg2	*Mus musculus*
P83127	2	58	38	813	3.39	-.LENGLLR.K	Alpha-N-acetylgalactosaminidase (Fragment)	naga	*Bos indicus*
Q3MHR7	1	3	41	974	0.13	K.DDDDVVIGK.V	Actin-related protein 2/3 complex subunit 2	arpc2	*Bos taurus*
P97592	1	2	37	731	0.17	R.DIMLLK.L	Mast cell protease 4	mcpt4	*Rattus norvegicus*
Q5Y5T3	3	2	32	1189	0.09	R.ITDTISDRLR.I	Palmitoyltransferase ZDHHC23	zdhhc23	*Mus musculus*
D4A8G3	2	2	31	814	0.17	K.VIPSEDR.A	Leucine rich adaptor protein 1	lurap1	*Rattus norvegicus*
Q60875	11	7	29	920	0.08	MTRSAVLK + Oxidation (M)	Rho guanine nucleotide exchange factor 2	arhgef2	*Mus musculus*
Q8BHL3	20	20	28	916	0.12	EQQKQEK	TBC1 domain family member 10B	tbc1d10b	*Mus musculus*
P14841	14	8	27	1207	0.13	GTHTLTKSSCK	Cystatin-C	cst3	*Rattus norvegicus*
Q5NUA6	8	11	27	772	0.17	QEQLQK	Nuclear factor erythroid 2-related factor 2	nfe2l2	*Bos taurus*
P21752	9	9	27	862	0.18	KTETQEK	Thymosin beta-10	tmsb10	*Bos taurus*
O46606	7	12	24	876	0.23	NPNFEEK	Phospholipase DDHD1	ddhd1	*Bos taurus*
Q9XS63	19	11	23	1113	0.26	GLGAERGQQAK	Chromogranin-A	chga	*Equus caballus*
O88807	17	12	22	1113	0.21	FSDNEDFLK	Protein-arginine deiminase type-4	padi4	*Rattus norvegicus*
Q6AYU1	17	16	22	949	0.33	YVDANLQK	Mortality factor 4-like protein 1	morf4l1	*Rattus norvegicus*
Q58DL1	10	8	21	973	0.71	SAGLMKR + Oxidation (M)	Arginase-2, mitochondrial	arg2	*Bos taurus*

P53620	22	10	21	1352	0.22	SVPLATAPLAEQR	Coatomer subunit gamma-1	copg1	*Bos taurus*
P59328	21	7	21	867	0.12	LLAVPVEK	WD repeat and HMG-box DNA-binding protein 1	wdhd1	*Mus musculus*
Q9EQJ9	13	8	21	1116	0.37	DSTEQLPDGR	Membrane-associated guanylate kinase, WW and PDZ domain-containing protein 3	magi3	*Mus musculus*
P26954	10	8	21	911	0.34	EKIPNPSK	Interleukin-3 receptor class 2 subunit beta	csf2rb2	*Mus musculus*
						**fraction 28**			
Q6IMF3	48	10	89	1384	0.79	K.SLNDKFASFIDK.V	Keratin, type II cytoskeletal 1	krt1	*Rattus norvegicus*
			71	826		K.FASFIDK.V			
			94	1475		R.FLEQQNQVLQTK.W			
			59	909		K.YEDEINK.R			
			52	1065		K.YEDEINKR.T			
			73	1265		R.TNAENEFVTIK.K			
			90	1393		R.TNAENEFVTIKK.D			
			66	1140		R.DYQELMNTK.L			
B3EWE1	11	17	64	998	0.51	R.TFASFPTTK.T	Haemoglobin subunit alpha	hba	*Blarina brevicauda*
			75	1793		K.TYFPHFDLSPGSAQVK.G			
P21571	6	14	53	827	0.63	K.ELDPVQK.L	ATP synthase-coupling factor 6, mitochondrial	atp5j	*Rattus norvegicus*
			59	1061		K.FEVLDKPQS.-			
Q6P0K8	3	2	40	846	0.11	R.NLSDVATK.Q	Junction plakoglobin	jup	*Rattus norvegicus*
			48	811		R.LVQLLVK.A			
Q6J3Q7	3	11	34	837	0.78	R.DACIIEK.G	Cytochrome c oxidase copper chaperone	cox17	*Canis lupus familiaris*
P14841	2	7	43	1207	0.22	K.GTHTLTKSSCK.N	Cystatin-C	cst3	*Rattus norvegicus*
D4A1J4	5	4	34	1307	0.17	R.QIDQFASEIEK.I	3-hydroxybutyrate dehydrogenase type 2	bdh2	*Rattus norvegicus*
P02754	1	4	36	915	0.23	K.IDALNENK.V	Beta-lactoglobulin	lgb	*Bos taurus*
P97592	4	2	35	731	0.17	R.DIMLLK.L	Mast cell protease 4	mcpt4	*Rattus norvegicus*
Q3ZC12	1	2	35	937	0.12	K.CVTSELLK.G	Eukaryotic translation initiation factor 3 subunit G	eif3g	*Bos taurus*
Q5Y5T3	1	2	32	1189	0.09	R.ITDTISDRLR.I	Palmitoyltransferase ZDHHC23	zdhhc23	*Mus musculus*

Q8VHN7	18	17	30	845	0.05	SLSLSLAR	G-protein coupled receptor 98	gpr98	*Mus musculus*
A0JNB0	1	2	29	1272	0.07	K.LDNGGYYITTR.A	Tyrosine-protein kinase Fyn	fyn	*Bos taurus*
Q3MHE8	9	8	27	982	0.08	MRDHLIAK	Signal recognition particle receptor subunit alpha	srpra	*Bos taurus*
Q9CSP9	13	9	27	1115	0.14	QEVEKLLEK	Tetratricopeptide repeat protein 14	ttc14	*Mus musculus*
O88351	23	10	26	1421	0.14	MKNAMASTAQQLK	Inhibitor of nuclear factor kappa-B kinase subunit beta	ikbkb	*Mus musculus*
**P01211**	**6**	**8**	**26**	**717**	**0.05**	**YGGFMK + Oxidation (M)**	**Proenkephalin-A**	**penk**	***Bos taurus***
Q9R002	10	8	26	1172	0.05	KQHNINYEK	Interferon-activable protein 202	ifi202	*Mus musculus*
D3ZAF6	14	9	25	1115	0.19	MASIVPLKEK	ATP synthase subunit f, mitochondrial	atp5j2	*Rattus norvegicus*
**Q61754**	**11**	**7**	**25**	**1204**	**0.16**	**DKSNDLMLLR**	**Kallikrein 1-related peptidase b24**	**klk1b24**	***Mus musculus***
Q5BIP7	14	8	24	1326	0.03	VFGRYVFSPVR	Lipoyl synthase, mitochondrial	lias	*Bos taurus*
**Q91V70**	**9**	**13**	**23**	**760**	**0.20**	**FQIPEK**	**Beta-defensin 7**	**defb7**	***Mus musculus***
Q2KJ64	13	7	22	1112	0.01	GGVEEGPTVLR	Arginase-1	arg1	*Bos taurus*
Q9R0T3	16	10	22	1058	0.23	MVAPGSVRSR	DnaJ homolog subfamily C member 3	dnajc3	*Rattus norvegicus*
**P12067**	**10**	**19**	**22**	**787**	**0.65**	**AWVAWR**	**Lysozyme C-1**	**lyz1**	***Sus scrofa***
P00687	4	5	22	780	0.43	DYVRTK	Alpha-amylase 1	amy1	*Mus musculus*
Q3T0L2	6	8	22	834	0.05	LAPSEYR	Endoplasmic reticulum resident protein 44	erp44	*Bos taurus*
P59328	7	7	21	867	0.17	LLAVPVEK	WD repeat and HMG-box DNA-binding protein 1	wdhd1	*Mus musculus*
Q6AYU1	13	12	21	949	0.52	YVDANLQK	Mortality factor 4-like protein 1	morf4l1	*Rattus norvegicus*
						**fraction 29**			
Q6IMF3	64	10	91	1384	0.91	K.SLNDKFASFIDK.V	Keratin, type II cytoskeletal 1	krt1	*Rattus norvegicus*
			71	826		K.FASFIDK.V			
			86	1475		R.FLEQQNQVLQTK.W			
			94	1475		K.WELLQQVDTSTR.T			
			58	909		K.YEDEINK.R			
			71	1065		K.YEDEINKR.T			

			85	1265		R.TNAENEFVTIK.K			
			54	1394		R.TNAENEFVTIKK.D			
			73	1140		R.DYQELMNTK.L			
			51	1156		R.DYQELMNTK.L+ Oxidation (M)			
Q8SPJ1	30	15	30	997	0.73	R.LAEPSQLLK.S	Junction plakoglobin	jup	*Bos taurus*
			40	770		R.ALPELTK.L			
			68	1341		K.LLNDEDPVVVTK.A			
			65	1352		R.TMQNTSDLDTAR.C			
			33	743		R.LADGLQK.M			
			31	813		K.MVPLLNK.N			
			33	875		K.LLWTTSR.V			
			69	846		R.NLSDVATK.Q			
			56	1001		K.QEGLESVLK.I			
			29	1368		R.HPEAEMAQNSVR.L			
			57	811		R.LVQLLVK.A			
			44	1235		R.ISEDKNPDYR.K			
			55	1236		R.VSVELTNSLFK.H			
Q0PGG4	6	5	87	975	0.22	K.AGFAGDDAPR.A	Actin, cytoplasmic 1	actb	*Bos mutus grunniensis*
			33	1131		R.GYSFTTTAER.E			
P21571	6	14	57	827	0.63	K.ELDPVQK.L	ATP synthase-coupling factor 6	atp5j	*Rattus norvegicus*
			59	1061		K.FEVLDKPQS.-			
Q2KJ64	7	8	45	1112	0.31	R.GGVEEGPTVLR.K	Arginase-1	arg1	*Bos taurus*
			31	1121		K.LKELECDVK.D			
			42	830		K.TPEEVTR.T			
C0HJG9	1	5	56	1244	0.20	R.TNQELQEINR.T	Annexin A2 (Fragments)	anxa2	*Mesocricetus auratus*
Q3C2I0	2	5	39	1169	0.23	K.ICETLCRLK.Q	Bcl-2-related protein A1	bcl2a1	*Bos taurus*
A0JPM9	1	4	35	1242	0.15	K.VLTPEEQLADK.L	Eukaryotic translation initiation factor 3 subunit J	eif3j	*Rattus norvegicus*
**Q61754**	**1**	**3**	**33**	**1204**	**0.11**	**K.DKSNDLMLLR.L**	**Kallikrein 1-related peptidase b24**	**klk1b24**	***Mus musculus***
P14841	14	8	27	1207	0.14	GTHTLTKSSCK	Cystatin-C	cst3	*Rattus norvegicus*
Q2TA68	5	13	26	794	0.07	WIYWK	Dynamin-like 120 kDa protein, mitochondrial	opa1	*Rattus norvegicus*
Q3T040	2	2	32	747	0.16	R.MLMPEK.L	28S ribosomal protein S7, mitochondrial	mrps7	*Bos taurus*
Q5Y5T3	2	2	32	1189	0.09	R.ITDTISDRLR.I	Palmitoyltransferase ZDHHC23	zdhhc23	*Mus musculus*

Q80TB8	1	2	29	1110	0.10	K.EPAEGGDGSHR.L	Synaptic vesicle membrane protein VAT-1 homolog-like	vat1l	*Mus musculus*
P00687	8	11	25	780	0.10	DYVRTK	α-amylase 1	amy1	*Mus musculus*
Q0VCX4	11	13	25	770	0.07	AIPELTK	Catenin beta-1	ctnnb1	*Bos taurus*
**Q91V70**	**8**	**11**	**24**	**760**	**0.46**	**FQIPEK**	**β-defensin 7**	**defb7**	***Mus musculus***
Q9R002	13	10	24	1172	0.16	KQHNINYEK	Interferon-activable protein 202	ifi202	*Mus musculus*
O08550	21	10	23	1342	0.21	TSSPLRTSPQLR	Histone-lysine N-methyltransferase 2B	kmt2b	*Mus musculus*
Q9R0T3	12	8	22	1058	0.42	MVAPGSVRSR	DnaJ homolog subfamily C member 3	dnajc3	*Rattus norvegicus*
Q62924	12	13	21	822	0.27	ELLFSSK	A-kinase anchor protein 11	akap11	*Rattus norvegicus*
Q75NR7	7	8	21	849	0.12	VPEPRPR	ATP-dependent DNA helicase Q4	recq14	*Mus musculus*
P59328	7	7	21	867	0.17	LLAVPVEK	WD repeat and HMG-box DNA-binding protein 1	wdhd1	*Mus musculus*
