# Supplementary material for: Proteins from shrews’ venom glands play a role in gland functioning and venom production
Source: Zoological Lett. 2024 Jul 15;10:12. doi: 10.1186/s40851-024-00236-x (PMC11251227; doi:10.1186/s40851-024-00236-x)
Supplement: Supplementary file 5 — Additional file 5: table A5: Biological functions of proteins identified in the extract from venom glands of the common shrew Sorex araneus based on tandem mass spectrometry analysis. Toxins are shown in bold. Function categories: 1 – Cell division & cell cycle regulation, 2 – Cell differentiation & tissue development, 3 – Cell migration, 4 – Cell structure maintenance, 5 – Cell aging & apoptosis, 6 – Signal transduction, 7 – Metabolism, 8 – Transport, 9 – Stress response, 10 – Immune response, 11 – DNA repair, 12 – Behaviour, 13 – Sensory function, 14 – unknown/not clear [file 40851_2024_236_MOESM5_ESM.pdf]

Table A5 Biological functions of proteins identified in the extract from venom glands of the common shrew *Sorex araneus* based on tandem mass spectrometry analysis. Toxins are shown in bold. Function categories: 1 – Cell division & cell cycle regulation, 2 – Cell differentiation & tissue development, 3 – Cell migration, 4 – Cell structure maintenance, 5 – Cell aging & apoptosis, 6 – Signal transduction, 7 – Metabolism, 8 – Transport, 9 – Stress response, 10 – Immune response, 11 – DNA repair, 12 – Behaviour, 13 – Sensory function, 14 – unknown/not clear.

**Accession code**	**Protein name**	**Biological function**	**Function category**
		**whole extract**	
P51434	Parvalbumin alpha (Fragment)	relaxation after muscle contraction	7
P63312	Thymosin beta-10	actin cytoskeleton organisation, cell migration regulation	3,4
P07107	Acyl-CoA-binding protein	lipid metabolism	7
P18203	Peptidyl-prolyl cis-trans isomerase FKBP1A	regulation of protein ubiquitination and phosphorylation, regulation of immune response, amyloid fibril formation, cytokine-mediated signalling pathway, T-cell activation and proliferation, response to caffeine, heart morphogenesis, muscle contraction	2,6,7,10
Q2KJ32	Selenium-binding protein 1	protein transport, oxidoreductase activity	8
Q2KJG2	Ubiquitin-fold modifier 1	brain development, regulation of intracellular signalling pathway, response to ER stress	2,6,9
P04906	Glutathione S-transferase P	animal organ regeneration, cell response to cell-matrix adhesion, glucocorticoid stimulus, insulin stimulus and LPS, cell proliferation, lipid metabolism, oligodendrocyte development, regulation of peroxide activity, regulation of fibroblast proliferation and IL-1 beta production, regulation of extrinsic apoptotic signalling pathway, response to amino acid, estradiol, ethanol, nutrient levels, reactive oxygen species and toxic substances	1,2,4-7,9,10
Q9DBJ1	Phosphoglycerate mutase 1	glycolysis	7
B3EWE1	Haemoglobin subunit alpha	oxygen transport	8
P21571	ATP synthase-coupling factor 6, mitochondrial	ATP metabolism, regulation of blood pressure and heart rate, ion transport, response to muscle activity	7,8
Q5E956	Triosephosphate isomerase	glycolysis	7
Q3MHL6	TSC22 domain family protein 1	transcription	7
Q3SZR3	Alpha-1-acid glycoprotein	transport, acute-phase response, regulation of immune system process	8,10
Q3T140	Dynein light chain roadblock-type 1	microtubule-based movement, transport	8
Q2KIV2	Mitochondrial import inner membrane translocase subunit Tim9	protein transport, translation	7,8
Q5FZP5	Secretogranin-2	angiogenesis, eosinophil chemotaxis	2,3
Q2EN75	Protein S100-A6	cell response to virus	10
Q9D0S9	Histidine triad nucleotide-binding protein 2, mitochondrial	apoptosis, lipid metabolism, steroid biosynthesis	5,7
Q8R317	Ubiquilin-1	regulation of protein ubiquitination, response to ER stress, regulation of oxidative stress-induced intrinsic apoptotic signalling pathway, autophagosome assembly and maturation, cellular response to hypoxia, macroautophagy	5-7,9

Q2HJ98	Acylpyruvase FAHD1, mitochondrial	pyruvate metabolic process	7
Q3ZC22	Heat shock factor-binding protein 1	stress response, cellular heat acclimation, axonal transport of mitochondrion	8,9
P47727	Carbonyl reductase [NADPH] 1	epithelial cell differentiation, lipid metabolism, response to hormones and LPS	2,7,9
Q8WNN6	Superoxide dismutase [Cu-Zn]	stress response, removal of superoxide radicals	9
P58044	Isopentenyl-diphosphate Delta-isomerase 1	cholesterol biosynthesis	7
Q3ZBS8	Mitochondrial import inner membrane translocase subunit Tim8 A	protein transport	8
Q5S3G4	Cytochrome c oxidase subunit 5B, mitochondrial	proton transmembrane transport, mitochondrial electron transport	8
Q8K2C6	NAD-dependent protein deacylase sirtuin-5, mitochondrial	cell apoptosis regulation, negative regulation of reactive oxygen species metabolism, protein metabolism, response to ischemia	5,7,9
P01283	VIP peptides	signalling pathway, epinephrine secretion, learning or memory, mRNA stabilisation, apoptosis regulation, positive regulation of endothelial cell proliferation, regulation of protein localization, regulation of sensory perception of pain	2,5-7,13
Q1ZZU7	Macrophage migration inhibitory factor	signalling pathway, DNA damage response, inflammatory response, apoptosis regulation, cellular senescence, prostaglandin biosynthesis	5-7,9,10
Q2NKU6	Protein dpy-30 homolog	transcription regulation, endosomal transport	7,8
P00435	Glutathione peroxidase 1	wound healing, cell migration and differentiation, cell redox homeostasis, fibroblast proliferation, heart contraction, apoptosis regulation, response to hydroperoxide, symbiotic bacterium, xenobiotic stimulus, sensory perception of sound, skeletal muscle tissue regeneration, temperature homeostasis, UV protection, vasodilation	2,3,5,7,9, 10
Q5E983	Elongation factor 1-beta	translational elongation	7
P00921	Carbonic anhydrase 2	signalling pathway, transport, positive regulation of dipeptide transmembrane transport, regulation of intracellular pH	6-8
Q6P7Q4	Lactoylglutathione lyase	carbohydrate metabolism, regulation of apoptosis and transcription, osteoblast differentiation	2,5,7
Q0IIJ2	Histone H1.0	DNA replication	1
Q9BDP9	Cocaine- and amphetamine-regulated transcript protein (Fragment)	adult feeding behaviour, cellular response to starvation, chemical synaptic transmission, circadian regulation of gene expression, G protein-coupled receptor signalling pathway, intracellular glucose homeostasis, negative regulation of appetite, neuropeptide signalling pathway, positive regulation of blood pressure, positive regulation of epinephrine secretion, positive regulation of transmission of nerve impulse	6,7,9,12
A2RUW1	Toll-interacting protein	autophagy, epithelial cell differentiation, inflammatory response, innate immune response, interleukin-1-mediated signalling pathway, phosphorylation, protein localization to endosome, signal transduction	2,6,7,10
Q4LAL9	Cathepsin D	autophagosome assembly, insulin catabolism, proteolysis	7
Q04447	Creatine kinase B-type	brain development, homeostasis, phosphorylation, biosynthesis	2,7
Q3YIX4	Phosphatidylethanolamine-binding protein 1	negative regulation of peptidase activity	7

Q9QZM0	Ubiquilin-2	autophagosome assembly, macroautophagy, negative regulation of clathrin-dependent endocytosis	7
Q3SZJ9	Phosphomannomutase 2	mannose metabolism, glycosylation	7
Q0PGG4	Actin, cytoplasmic 1	cell motility and contraction, transcription regulation, DNA damage response, DNA repair	3,7,11
Q9N0F1	Dihydrolipoyllysine-residue succinyltransferase component of 2-oxoglutarate dehydrogenase complex, mitochondrial	metabolic processes	7
P62958	Histidine triad nucleotide-binding protein 1	transcription regulation, catabolism, apoptosis, signalling pathway	5-7
Q8BH83	Ankyrin repeat domain-containing protein 9	intracellular copper ion homeostasis, protein ubiquitination	7
Q56K04	Cysteine-rich protein 1	regulation of gene expression, intrinsic apoptotic signalling pathway, response to DNA damage, cell response to antibiotic, UV-B and zinc ion	6,9
Q3SZ18	Hypoxanthine-guanine phosphoribosyltransferase	GMP catabolism, biosynthesis	7
Q6B4U9	Peroxiredoxin-1	erythrocyte homeostasis, fibroblast proliferation, hydrogen peroxide catabolism, natural killer cell activation, natural killer cell mediated cytotoxicity, signalling pathway, removal of superoxide radicals	1,2,6,9,10
Q3ZBD3	Pterin-4-alpha-carbinolamine dehydratase	biosynthesis, transcription regulation	7
Q9D6Y7	Mitochondrial peptide methionine sulfoxide reductase	aging, cell response to oxidative stress	5,9
P04444	Haemoglobin subunit beta-H1	oxygen and carbon dioxide transport, cellular oxidant detoxification, hydrogen peroxide catabolic process, regulation of transcription and apoptosis	5,7-9
Q9JLS0	Hypoxia-inducible lipid droplet-associated protein	autocrine signalling, cellular response to hypoxia, regulation of cell population proliferation and cytokine production, regulation of lipid storage	1,6,7,9,10
Q8BHX3	Borealin	mitotic cell cycle, cell division, protein phosphorylation	1,7
Q3UDR8	Protein YIPF3	cell differentiation	2
Q2YDH6	AP-3 complex subunit sigma-1	intracellular protein transport, vesicle-mediated transport, Golgi to vacuole transport	8
Q3C2I0	Bcl-2-related protein A1	apoptosis regulation, intrinsic apoptotic signalling pathway in response to DNA damage, mitochondrial fusion, release of cytochrome c from mitochondria	5-7,9
Q8BIQ3	Zinc finger protein 2	transcription regulation	7
Q0Z8U2	40S ribosomal protein S3	apoptosis, cell division, DNA repair, cell response to hydrogen peroxide, translation regulation, regulation of protein ubiquitination, positive regulation of activated T cell proliferation, signalling pathway, positive regulation of interleukin-2 production	1,5-7,9-11
B1ARW8	Uncharacterized protein C1orf122 homolog	unknown/not clear	14
Q99LJ8	Dehydrodolichyl diphosphate synthase complex subunit Nus1	angiogenesis, cell differentiation and migration, lipid metabolism and transport	2,3,7,7
O77512	Glycine N-phenylacetyltransferase	unknown/not clear	14

Q5HZE0	Mitochondrial basic amino acids transporter	transport	8
Q9D483	DNA-directed RNA polymerase III subunit RPC3	defence to virus, innate immune response, transcription regulation	7,10
Q3UHX2	28 kDa heat- and acid-stable phosphoprotein	platelet-derived growth factor receptor signalling pathway	6
Q5GAL7	Probable inactive ribonuclease-like protein 13	defence response to Gram-positive bacterium	10
O35507	Peroxisome proliferator-activated receptor alpha	cell response to starvation, epidermis development, circadian regulation of gene expression, lipid metabolism, negative regulation of appetite, negative regulation of cholesterol storage, glycolysis regulation, regulation of inflammatory response, leukocyte cell-cell adhesion, cell differentiation, transcription regulation, biosynthesis, signalling pathway, wound healing	2,4,6,7,9,10
A2AIW0	Serologically defined colon cancer antigen 3 homolog	cell cycle, cell division, protein transport	1,8
Q9R0M6	Ras-related protein Rab-9A	exocytosis regulation, protein transport	7,8
Q9D8Z2	TP53-regulated inhibitor of apoptosis 1	apoptosis, cell response to UV, DNA damage response, phospholipid transport, transcription regulation	5,7,9
Q5BIP7	Lipoyl synthase, mitochondrial	inflammatory response, response to LPS and oxidative stress, biosynthesis	7,9,10
Q3SZI5	Mitochondrial uncoupling protein 2	adaptive thermogenesis, glycolysis, macrophage differentiation, mitochondrial fission, mitochondrial transmembrane transport, response to cold, hypoxia	1,2,7-9
Q8BYH0	Dorsal root ganglia homeobox protein	neuron differentiation and migration, transcription regulation, sensory perception of mechanical stimulus, detection of chemical and temperature stimulus	2,3,7,9,13
Q80W32	IQ domain-containing protein G	cell differentiation	2
Q8BGQ6	EF-hand calcium-binding domain-containing protein 14	metal ion binding	7
Q9CZ69	CKLF-like MARVEL transmembrane domain-containing protein 6	protein transport	8
Q7TMI3	E3 ubiquitin-protein ligase UHRF2	cell cycle, cell differentiation, protein ubiquitination	1,2,7
Q8BKV1	Glypican-2	cell migration, neuron differentiation, regulation of protein localization to membrane, signal transduction	2,3,6,8
P26260	Syndecan-1	Wnt signalling pathway, cell adhesion and migration, cell-cell signalling	3,4,6
Q08DK5	Endophilin-B2	membrane organisation	4
Q865B6	Peroxisome proliferator-activated receptor gamma coactivator 1-alpha	adipose tissue development, cell response to oxidative stress, energy homeostasis, gluconeogenesis, mitochondrion organisation, circadian regulation of gene expression, regulation of circadian rhythym, response to dietary excess and muscle activity, neuron apoptosis, regulation of ATP biosynthesis, regulation of smooth muscle cell proliferation, regulation of cellular respiration, transcription regulation, regulation of muscle tissue development	1,2,4,5,7,9
Q3SZE2	Prefoldin subunit 1	protein folding, fibril formation	7

Q8VC56	E3 ubiquitin-protein ligase RNF8	cell cycle, cell division, DNA damage response, DNA repair, transcription regulation, protein ubiquitination, signal transduction in response to DNA damage	1,6,7,9,11
Q0P565	HD domain-containing protein 2	metal ion binding	7
Q9ERA5	Structural maintenance of chromosomes protein 4 (Fragment)	cell cycle, cell division	1
Q11126	Galactoside 3(4)-L-fucosyltransferase	lipid metabolism, cell-cell adhesion, regulation of cell migration and cell population proliferation	1,3,4,7
Q5H8C4	Vacuolar protein sorting-associated protein 13A	autophagy, lipid transport, locomotory behaviour, lysosomal protein catabolism, nervous system development, protein targeting to vacuole, protein retention in Golgi apparatus	2,7,8,12
P19803	Rho GDP-dissociation inhibitor 1	Rho protein signal transduction	6
O97594	Structural maintenance of chromosomes protein 3	cell cycle, cell division, DNA damage, DNA repair	1,9,11
Q5EAD3	Transcription factor NF-E2 45 kDa subunit	transcription regulation	7
P15690	NADH-ubiquinone oxidoreductase 75 kDa subunit, mitochondrial	apoptosis, cellular respiration, mitochondrial electron transport, mitochondrial respiratory chain, reactive oxygen species metabolism	5,7,8
Q91YS8	Calcium/calmodulin-dependent protein kinase type 1	cell cycle, cell differentiation, neurogenesis, regulation of synapse organisation, transcription regulation, regulation of protein localization, protein binding and phosphorylation, regulation of muscle cell differentiation, signal transduction, nucleocytoplasmic transport	1,2,4,4-8
Q8CE90	Dual specificity mitogen-activated protein kinase 7	apoptosis, transcription regulation, cell response to IL-1, LPS, heat, UV, osmotic stress and wounding, stress response, protein phosphorylation	5,7,9
P97313	DNA-dependent protein kinase catalytic subunit	DNA damage response, protein phosphorylation, apoptosis regulation, brain, heart and spleen development, leukocyte differentiation, innate immune response, regulation of erythrocyte differentiation and fibroblast proliferation, translation regulation, regulation of platelet formation	1,2,5,7,9,10
P33705	T-cell surface glycoprotein CD4	adaptive immune response, cell adhesion	4,10
O47558	Cytochrome b (Fragment)	respiratory electron transport chain	8
Q9D483	DNA-directed RNA polymerase III subunit RPC3	innate immune response, defence response to virus, transcription regulation	7,10
P62157	Calmodulin	positive and negative regulation of ryanodine-sensitive calcium-release channel activity, regulation of release of sequestered calcium ion into cytosol by sarcoplasmic reticulum	7
Q9BEG2	Interleukin-12 receptor subunit beta-2	signalling pathway, immunity	6,10
Q8BLK9	Ribosomal protein S6 kinase delta-1	protein phosphorylation	7
G3MWR8	Protein-methionine sulfoxide oxidase MICAL3	cytoskeleton organisation, exocytosis	4,7
P05126	Protein kinase C beta type	apoptosis, calcium ion transport, adaptive immune response, chromatin organisation, intracellular signal transduction, transmembrane transport, B-cell activation, angiogenesis, insulin secretion, transcription, regulation of synaptic vesicle exocytosis	2,4,5-8,10
Q3MHE4	DNA mismatch repair protein Msh2	DNA damage, DNA repair, cell cycle, B-cell differentiation, apoptosis, response to UV and X-ray	1,2,5,9,11
O35314	Secretogranin-1	secretion	7

E9Q6J5	Biorientation of chromosomes in cell division protein 1-like 1	DNA damage, DNA repair and replication	1,9,11
P26954	Interleukin-3 receptor class 2 subunit beta	cytokine-mediated signalling pathway, immune response, leukocyte proliferation, protein phosphorylation	2,6,7,10
A3KFM7	Chromodomain-helicase-DNA-binding protein 6	cell redox homeostasis, transcription regulation	7
Q330H0	NADH-ubiquinone oxidoreductase chain 2	mitochondrial electron transport	8
Q9JLM2	Natural killer cell receptor 2B4	myeloid dendritic cell activation, immune response, positive regulation of interleukin-8 production, positive regulation of natural killer cell proliferation and type II interferon production	10
Q3SZK4	Protein TBRG4	mitochondrial mRNA processing, mRNA metabolism	7
P57784	U2 small nuclear ribonucleoprotein A'	mRNA splicing	7
Q8K202	DNA-directed RNA polymerase I subunit RPA49	transcription	7
**Q10741**	**Disintegrin and metalloproteinase domain-containing protein 10**	**protein catabolism, proteolysis, monocyte activation, regulation of cell adhesion, cell growth and cell population proliferation, protein phosphorylation, regulation of Notch signalling pathway, regulation of vasculature development, toxin transport**	**1,2,4,6-8,10**
P23726	Phosphatidylinositol 3-kinase regulatory subunit beta	protein transport, signalling pathway, transcription regulation, autophagy, cell adhesion regulation, stress response, actin biosynthesis, filament polymerisation	4,6-9
Q6NZP1	DNA annealing helicase and endonuclease ZRANB3	DNA damage response, DNA repair	9,11
Q3SX42	Charged multivesicular body protein 2b	protein transport and catabolism, apoptosis regulation, neuron cellular homeostasis, autophagosome maturation, cognition, endosome organisation, plasma membrane repair	4,5,7,8
Q9N0J6	Fructose-1,6-bisphosphatase isozyme 2	gluconeogenesis	7
Q8BW10	RNA-binding protein NOB1	rRNA processing, visual perception	7,13
Q8VD75	Huntingtin-interacting protein 1	actin filament organisation, apoptosis, signalling pathway, cell differentiation, endocytosis, protein stabilisation	2,4-7
Q3SYS1	39S ribosomal protein L13, mitochondrial	translation regulation	7
Q60560	DNA-binding protein SMUBP-2	transcription regulation	7
P50310	Phosphoglycerate kinase 1	glycolysis, phosphorylation	7
Q58DC5	GTP-binding protein 1	GTP metabolism, mRNA catabolism, translational elongation	7
A5PK65	D-dopachrome decarboxylase	melanin biosynthesis	7
Q8CFD4	Sorting nexin-8	intracellular protein transport	8
P28800	Alpha-2-antiplasmin	regulation of stress fibre assembly, regulation of smooth muscle cell proliferation, transcription regulation, acute-phase response, blood vessel morphogenesis, collagen fibril organisation, regulation of plasminogen activation, cell differentiation, collagen biosynthesis	1,2,4,7,10

Q91Y47	Coagulation factor XI	plasminogen activation, blood coagulation	10
Q63880	Carboxylesterase 3A	detoxification of xenobiotics	9
Q9Z2E1	Methyl-CpG-binding domain protein 2	Wnt signalling pathway, regulation of cell population proliferation, transcription regulation, response to mechanical stimulus and nutrient levels, aging, heart development, maternal behaviour	1,2,6,7,9, 12
O55036	Telomeric repeat-binding factor 1 (Fragment)	cell cycle, cell division	1
P19973	Lymphocyte-specific protein 1	apoptosis, cytoskeleton organisation, chemotaxis, defence response, signal transduction	3-6,10
Q80WC3	Trinucleotide repeat-containing gene 18 protein	chromatin binding	1
		**fraction 23**	
Q6IMF3	Keratin, type II cytoskeletal 1	keratinization, negative regulation of inflammatory response	4,10
Q5XLE4	Serum albumin	cell response to starvation, maintenance of mitochondrion location, apoptosis regulation	4,5,9
P34032	Thymosin beta-4	actin filament organisation	4
B3EWE1	Haemoglobin subunit alpha	oxygen transport	8
P18174	Involucrin	keratinization	4
Q07DX1	Caveolin-1	signalling pathway, T cell co-stimulation	6,10
Q3T0F4	40S ribosomal protein S10	translation	7
Q3V1I0	Lysozyme g-like protein 2	defence response to bacterium, catabolism	7,10
P83127	Alpha-N-acetylgalactosaminidase (Fragment)	carbohydrate catabolism	7
Q3MHR7	Actin-related protein 2/3 complex subunit 2	actin filament polymerization	4
P97592	Mast cell protease 4	proteolysis	7
Q5Y5T3	Palmitoyltransferase ZDHHC23	protein targeting to membrane	8
D4A8G3	Leucine rich adaptor protein 1	actomyosin structure organization, cell migration, positive regulation of cytokine production, signalling pathway	3,4,6,10
Q60875	Rho guanine nucleotide exchange factor 2	actin filament organisation, cell morphogenesis, transcription regulation, cell cycle, cell division, neurogenesis regulation, signal transduction, neuron differentiation and migration, regulation of IL-6 production, innate immune response	1-4,6,7,10
Q8BHL3	TBC1 domain family member 10B	activation and regulation of GTPase activity, retrograde transport	7,8
P14841	Cystatin-C	apoptosis, brain and eye development, cell response hydrogen peroxide, oxidative stress, circadian sleep/wake cycle	2,5,7,9
Q5NUA6	Nuclear factor erythroid 2-related factor 2	cell redox homeostasis, transcription regulation, cell response to oxidative stress, protein catabolism, protein ubiquitination	7,9
P21752	Thymosin beta-10	actin filament organisation, regulation of cell migration	3,4
O46606	Phospholipase DDHD1	lipid metabolism	7
Q9XS63	Chromogranin-A	defence response to bacterium, endocrine process, regulation of catecholamine and insulin secretion, mast cell activation, chemotaxis and degradation	3,7,10
O88807	Protein-arginine deiminase type-4	transcription, innate immune response, post-translational protein modification	7,10

Q6AYU1	Mortality factor 4-like protein 1	DNA damage, DNA repair, cell population proliferation, transcription, cell cycle regulation, chromatin organisation	1,7,9,11
Q58DL1	Arginase-2, mitochondrial	arginine catabolism, adaptive and innate immune response, urea cycle	7,10
P53620	Coatomer subunit gamma-1	protein secretion, intracellular protein transport, organelle transport along microtubule, ER-Golgi transport	7,8
P59328	WD repeat and HMG-box DNA-binding protein 1	mitotic cell cycle, DNA replication, DNA repair, RNA processing	1,7,11
Q9EQJ9	Membrane-associated guanylate kinase, WW and PDZ domain-containing protein 3	signal transduction	6
P26954	Interleukin-3 receptor class 2 subunit beta	cytokine receptor activity, Ig mediated immune response, cytokine-mediated signalling pathway, regulation of leukocyte proliferation, protein phosphorylation	1,6,7,10
		**fraction 28**	
Q6IMF3	Keratin, type II cytoskeletal 1	as above	4,10
B3EWE1	Haemoglobin subunit alpha	oxygen transport	8
P21571	ATP synthase-coupling factor 6, mitochondrial	as above	7,8
Q6P0K8	Junction plakoglobin	cell communication, cell migration and adhesion, Wnt signalling pathway, cell response to indole-3-methanol	3,4,6,7
Q6J3Q7	Cytochrome c oxidase copper chaperone	mitochondrial cytochrome c oxidase assembly, positive regulation of cell population proliferation, positive regulation of cytochrome-c oxidase activity	1,7
P14841	Cystatin-C	as above	2,5,7,9
D4A1J4	3-hydroxybutyrate dehydrogenase type 2	epithelial cell differentiation, lipid metabolism	2,7
P02754	Beta-lactoglobulin	transport	8
P97592	Mast cell protease 4	proteolysis	7
Q3ZC12	Eukaryotic translation initiation factor 3 subunit G	protein biosynthesis	7
Q5Y5T3	Palmitoyltransferase ZDHHC23	protein targeting to membrane	8
Q8VHN7	G-protein coupled receptor 98	cell-cell adhesion, establishment of protein localisation, cell response to calcium ion, regulation of bone mineralisation, nervous system development, cell surface receptor signalling pathway, regulation of protein stability, sensory perception of sound, visual perception, self-proteolysis	2,4,6,7,13
A0JNB0	Tyrosine-protein kinase Fyn	adaptive immune response, activated T cell proliferation, signalling pathway, cell shape regulation, protein ubiquitination and catabolism, protein transport, neuron migration, dendrite morphogenesis, cellular response to platelet-derived growth factor stimulus, cellular response to transforming growth factor beta stimulus, heart processes	2-4,6-8,10
Q3MHE8	Signal recognition particle receptor subunit alpha	protein targeting to ER	8
Q9CSP9	Tetratricopeptide repeat protein 14	nucleic acid binding	7

O88351	Inhibitor of nuclear factor kappa-B kinase subunit beta	neuron apoptosis regulation, neuron projection development, regulation of sodium ion transport, actin cytoskeleton organisation, signalling pathway, regulation of cell population proliferation, protein phosphorylation	1,2,4-8
**P01211**	**Proenkephalin-A**	**sensory perception of pain, neuropeptide signalling pathway, defence response to bacterium, locomotory behaviour, aggressive behaviour, behavioural fear response, chemical synaptic transmission**	**6,10,12,13**
Q9R002	Interferon-activable protein 202	innate immune response, inflammatory response, response to bacterium, regulation of IL-1 beta production, apoptosis	5,10
D3ZAF6	ATP synthase subunit f, mitochondrial	ATP metabolism, proton transmembrane transport, ion transport	7,8
**Q61754**	**Kallikrein 1-related peptidase b24**	**proteolysis, zymogen activation, regulation of systemic arterial blood pressure**	**7**
Q5BIP7	Lipoyl synthase, mitochondrial	inflammatory response, response to LPS and oxidative stress, biosynthesis	7,9,10
**Q91V70**	**Beta-defensin 7**	**cell chemotaxis, defence response to bacterium**	**3,10**
Q2KJ64	Arginase-1	arginine catabolism, defence response to protozoan, regulation of activated T-cell proliferation, regulation of neutrophil-mediated killing of fungus, urea cycle	7,10
Q9R0T3	DnaJ homolog subfamily C member 3	apoptosis regulation, cell response to cold, unfolded protein and ER stress, protein catabolism	5,7,9
**P12067**	**Lysozyme C-1**	**digestion, metabolism, defence response to Gram positive bacterium, killing of cell of another organism**	**7,10**
P00687	Alpha-amylase 1	carbohydrate metabolism, response to bacterium	7,10
Q3T0L2	Endoplasmic reticulum resident protein 44	protein folding	7
P59328	WD repeat and HMG-box DNA-binding protein 1	mitotic cell cycle, DNA replication, DNA repair, RNA processing	1,7,11
Q6AYU1	Mortality factor 4-like protein 1	cell population proliferation, cell cycle regulation, transcription regulation, DNA damage, DNA repair, DNA recombination, growth regulation	1,4,7,9,11
		**fraction 29**	
Q6IMF3	Keratin, type II cytoskeletal 1	as above	4,10
Q8SPJ1	Junction plakoglobin	cell communication, cell migration and adhesion, Wnt signalling pathway, cell response to indole-3-methanol	3,4,6,7
Q0PGG4	Actin, cytoplasmic 1	as above	3,7,11
P21571	ATP synthase-coupling factor 6	as above	7,8
Q2KJ64	Arginase-1	as above	7,10
C0HJG9	Annexin A2 (Fragments)	cytoskeletal protein binding, phospholipase inhibitor activity	4,7
Q3C2I0	Bcl-2-related protein A1	as above	5-7,9
A0JPM9	Eukaryotic translation initiation factor 3 subunit J	protein biosynthesis	7
**Q61754**	**Kallikrein 1-related peptidase b24**	**as above**	**7**
P14841	Cystatin-C	as above	2,5,7,9

Q2TA68	Dynamin-like 120 kDa protein, mitochondrial	aging, apoptosis, calcium import into mitochondrion, GTP metabolism, cell response to hypoxia, inner mitochondrial membrane organisation, visual perception, sensory transduction, response to electrical stimulus and nutrient levels, signalling pathway, regulation of dendrite development	2,4-9,13
Q3T040	28S ribosomal protein S7, mitochondrial	mitochondrial translation	7
Q5Y5T3	Palmitoyltransferase ZDHHC23	protein targeting to membrane	8
Q80TB8	Synaptic vesicle membrane protein VAT-1 homolog-like	oxidoreductase activity	7
P00687	α-amylase 1	as above	7,10
Q0VCX4	Catenin beta-1	neuron migration, cell adhesion and differentiation, bone resorption, Wnt signalling pathway, blood vessel morphogenesis, osteoblast differentiation, cell maturation, cell-matrix adhesion, cell response to growth factor stimulus, chemical synaptic transmission, ectoderm development, chondrocyte differentiation, regulation of apoptosis and transcription, regulation of mitotic cell cycle, regulation of smooth muscle cell and T-cell proliferation, protein ubiquitination, response to estradiol and xenobiotic stimulus, thymus development, synaptic vesicle transport	1-9
**Q91V70**	**β-defensin 7**	**as above**	**3,10**
Q9R002	Interferon-activable protein 202	as above	5,10
O08550	Histone-lysine N-methyltransferase 2B	transcription regulation	7
Q9R0T3	DnaJ homolog subfamily C member 3	as above	5,7,9
Q62924	A-kinase anchor protein 11	actin cytoskeleton organisation, protein phosphorylation, protein localisation to endosome	4,7,8
Q75NR7	ATP-dependent DNA helicase Q4	DNA replication, cell population proliferation, cell division, skeletal system development	1,2
P59328	WD repeat and HMG-box DNA-binding protein 1	as above	1,7,11
